# H_2_S-activatable near-infrared afterglow luminescent probes for sensitive molecular imaging in vivo

**DOI:** 10.1038/s41467-020-14307-y

**Published:** 2020-01-23

**Authors:** Luyan Wu, Yusuke Ishigaki, Yuxuan Hu, Keisuke Sugimoto, Wenhui Zeng, Takashi Harimoto, Yidan Sun, Jian He, Takanori Suzuki, Xiqun Jiang, Hong-Yuan Chen, Deju Ye

**Affiliations:** 1 State Key Laboratory of Analytical Chemistry for Life Science, School of Chemistry and Chemical Engineering, Nanjing University, Nanjing, 210023 China; 2 Department of Chemistry, Faculty of Science, Hokkaido University, N10 W8, North-ward, Sapporo, 060-0810 Japan; 3 Department of Radiology, Nanjing Drum Tower Hospital, The Affiliated Hospital of Nanjing University Medical School, Nanjing, 210008 China; 4 MOE Key Laboratory of High Performance Polymer Materials and Technology, School of Chemistry and Chemical Engineering, Nanjing University, Nanjing, 210023 China; 5 Chemistry and Biomedicine Innovation Center (ChemBIC), Nanjing University, Nanjing, 210023 China

**Keywords:** Bioanalytical chemistry, Nanoparticles, Nanoparticles

## Abstract

Afterglow luminescent probes with high signal-to-background ratio show promise for in vivo imaging; however, such probes that can be selectively delivered into target sites and switch on afterglow luminescence remain limited. We optimize an organic electrochromic material and integrate it into near-infrared (NIR) photosensitizer (silicon 2,3-naphthalocyanine bis(trihexylsilyloxide) and (poly[2-methoxy-5-(2-ethylhexyloxy)-1,4-phenylenevinylene]) containing nanoparticles, developing an H_2_S-activatable NIR afterglow probe (**F1**^2+^-ANP). **F1**^2+^-ANP displays a fast reaction rate (1563 ± 141 M^−1^ s^−1^) and large afterglow turn-on ratio (~122-fold) toward H_2_S, enabling high-sensitivity and -specificity measurement of H_2_S concentration in bloods from healthy persons, hepatic or colorectal cancer patients. We further construct a hepatic-tumor-targeting and H_2_S-activatable afterglow probe (**F1**^2+^-ANP-Gal) for noninvasive, real-time imaging of tiny subcutaneous HepG2 tumors (<3 mm in diameter) and orthotopic liver tumors in mice. Strikingly, **F1**^2+^-ANP-Gal accurately delineates tumor margins in excised hepatic cancer specimens, which may facilitate intraoperative guidance of hepatic cancer surgery.

## Abstract (web summary)

Afterglow imaging probes are of interest for in vivo imaging due to high signal-to-background ratios. Here, the authors report on an afterglow nanoparticle which is activated by hydrogen sulphide and demonstrate the measurement of hydrogen sulphide levels and the labelling of hepatic cancer specimens in vivo.

## Introduction

Fluorescence imaging probes are indispensable in biomedical research and clinical diagnosis owing to their low cost, simple operation, and noninvasiveness; these characteristics enable real-time detection of physiological and pathological processes at the molecular and cellular levels^1–4^. However, because fluorescence imaging requires real-time light excitation, the inevitable light absorption, scattering, and autofluorescence of biological tissues can limit penetration depth and elicit a low signal-to-background ratio (SBR), compromising the sensitivity for in vivo imaging^5–7^. Afterglow luminescent probes (also called persistent luminescent probes), which can trap excitation energy in defects and slowly release photons after cessation of light excitation, have recently emerged as promising tools for overcoming the limitations of fluorescence probes in biosensing and molecular imaging^8–11^. Afterglow imaging with these luminescent probes does not require real-time excitation with an external light, causing no autofluorescence interference from biological tissues and offering greatly improved SBR and sensitivity to visualize disease sites in vivo^12–15^.

During the past few years, several prominent afterglow probes have been developed using rare-earth heavy metal ion (e.g., europium or praseodymium)-doping inorganic nanoparticles^9,16–20^ or organic and polymeric afterglow materials^4,21–24^. Richard and co-workers^25^ optimized chromium-doped zinc gallate (ZnGa_1.995_Cr_0.005_O_4_) to develop inorganic nanoparticles that can emit intense long-lasting near-infrared (NIR) afterglow luminescence for noninvasive tracking of labeled cells and imaging of vascularization and grafted tumor cells. Pu and co-workers^26^ proposed poly[2-methoxy-5-(2-ethylhexyloxy)-1,4-phenylenevinylene] (MEH-PPV)-based semiconducting polymer nanoparticles (SPNs) to develop biocompatible organic afterglow probes for sensitive detection of tumor metastasis and monitoring of hepatotoxicity in vivo^26^. Despite encouraging results, these afterglow probes encounter limitations when applied for in vivo diagnosis. Inorganic nanoparticle-based afterglow probes may be hampered by systemic toxicity concerns related to potential leakage of heavy metal ions^27,28^. Current MEH-PPV-based organic afterglow probes could be compromised by poor targeting ability to facilitate efficient delivery and accumulation in disease sites (e.g., tumors) after systemic injection^29–31^. In addition, most afterglow probes are designed as “always-on” luminescent probes; a continuous signal may produce a high background signal regardless of proximity or interaction with the molecular target of interest, thereby interfering with sensitivity and real-time imaging capacity^13,25,27,29^. By contrast, activatable afterglow luminescent probes with “off–on” signals in response to a biological target of interest can substantially reduce the background signal, improving SBR for real-time imaging. However, given a lack of effective activation approaches, activatable afterglow probes have been rarely developed for in vivo imaging^26,32^.

Hydrogen sulfide (H_2_S) is a highly reactive endogenous signaling molecule, playing a critical role in diverse physiological functions^33–35^. Aberrant concentration and tissue distribution of H_2_S are associated with many diseases (e.g., liver inflammation, hypertension, diabetes, and cancers)^36–39^. Therefore, precise spatiotemporal detection of H_2_S in living subjects is essential to the study of the biological functions and accurate diagnosis of H_2_S-related diseases. Although numerous H_2_S-activatable fluorescent probes have been developed, the insufficient reaction kinetics, poor in vivo targeting ability, and limited tissue penetration of fluorescence have restricted the capacity to detect H_2_S’ concentrations and locations in living animals^40–46^. Recently, we reported the engineering of an organic π-electron structure-based electrochromic material (EM **1**^2+^) as an H_2_S-responsive chromophore, which was amenable to build H_2_S-activatable NIR fluorescent probes for noninvasive imaging of H_2_S in living mice^47^.

In this paper, we optimize the structure of EM **1**^2+^ into a bis(pentafluorophenyl)-substituted EM **F1**^2+^ that exhibits longer absorption wavelengths and significantly improved reaction kinetics toward H_2_S under physiological conditions. We showcase its feasibility to control afterglow luminescence from MEH-PPV (580 nm) and doping NIR photosensitizer (silicon 2,3-naphthalocyanine bis(trihexylsilyloxide), NIR775; 780 nm) when integrated into an MEH-PPV- and NIR775-containing afterglow nanoparticle (**F1**^2+^-ANP). Significant activation of afterglow luminescence by a factor of ~122-fold can be achieved by incubating **F1**^2+^-ANP with H_2_S within 1 min, followed by irradiation with an 808-nm laser, allowing for rapid quantification of the H_2_S concentration in blood samples of healthy persons and patients diagnosed with hepatic cancers (HCCs) or colorectal cancers (CRCs). Moreover, through incorporation of β-galactose (β-Gal) ligands on the surface, we build a hepatic tumor-targeting and H_2_S-activatable afterglow probe (**F1**^2+^-ANP-Gal) that offers sensitive detection of subcutaneous and orthotopic liver tumors in living mice and delineation of liver tumor lesions in clinically excised HCC specimens. The demonstration of H_2_S-activatable afterglow luminescent probes feasible for sensitive and reliable detection of endogenous H_2_S underscores the potential of smart activatable afterglow luminescent probes for in vivo imaging.

## Results

### Design of H_2_S-activatable NIR afterglow probe

**Fig. 1:**
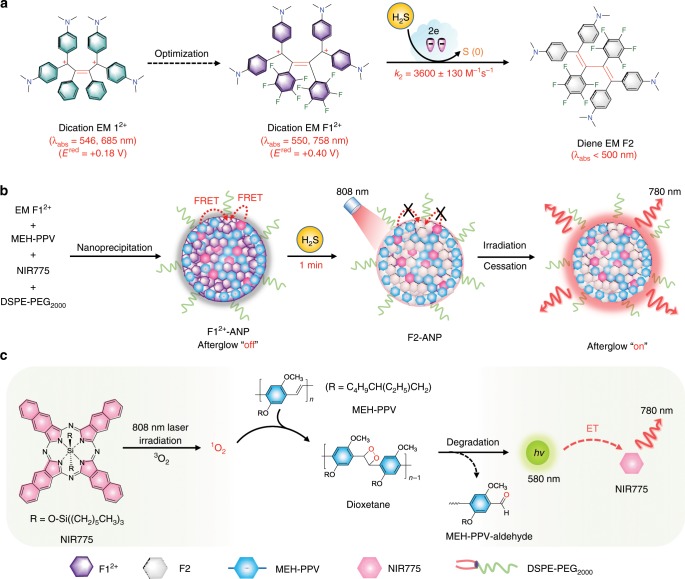
Schematic illustration of the design of **F1**^2+^-ANP. **a** Optimization of dication EM **1**^2+^ into **F1**^2+^ by introducing two electron-withdrawing pentafluorophenyl groups, and proposed chemical conversion of **F1**^2+^ into diene **F2** upon reduction by H_2_S. **b** Preparation of **F1**^2+^-ANP and proposed mechanism of H_2_S-mediated fast activation of NIR afterglow luminescence at 780 nm following pre-irradiation with an 808-nm laser. **c** The detailed photoreaction processes to produce NIR afterglow luminescence within activated **F1**^2+^-ANP (i.e., **F2**-ANP). **F1**^2+^-ANP was designed to contain hydrophobic EM **F1**^2+^, MEH-PPV, and NIR775 via DSPE-PEG_2000_-assisted nanoprecipitation. **F1**^2+^-ANP is initially afterglow “off” owing to the presence of **F1**^2+^ that can quench both the fluorescence and ^1^O_2_ production of MEH-PPV and NIR775 via two efficient FRET processes. Upon reaction with H_2_S, **F1**^2+^ was reduced into **F2**, and the FRET-based quenching processes were eliminated within **F2**-ANP, resulting in the recovery of fluorescence and ^1^O_2_ production capacity. Upon irradiation with the 808-nm laser, NIR775 can sensitize ^3^O_2_ to generate ^1^O_2_, which then oxidizes the vinylene bond of MEH-PPV to form unstable dioxetane. After cessation of irradiation, the subsequent slow degradation into MEH-PPV-aldehyde and releases photons at 580 nm, followed by efficient intraparticle ET to NIR775, ultimately switching “on” NIR afterglow luminescence at 780 nm.

Figure 1 shows the design of an H_2_S-activatable NIR afterglow probe (**F1**^2+^-ANP). NIR775 was chosen as an NIR-excitable photosensitizer due to its stronger ability to generate singlet oxygen (^1^O_2_) compared to MEH-PPV, amplifying the afterglow of MEH-PPV upon pre-irradiation by 808-nm light^26,47^. Furthermore, efficient intraparticle energy transfer (ET) from MEH-PPV to NIR775 can promote a red shift of afterglow wavelength into the NIR window, permitting improved penetration depth in biological tissues. We employed EM **F1**^2+^ as a H_2_S-responsive chromophore because it is designed to have longer ultraviolet–visible–NIR (UV–visible–NIR) absorptions (550 and 758 nm) than EM **1**^2+^ (546 and 685 nm), ensuring extensive spectral overlaps with MEH-PPV and NIR775 emissions (Fig. 1a). Additionally, EM **F1**^2+^ possesses a more positive reduction potential (*E*^red^ = + 0.40 V vs. SCE) than EM **1**^2+^ (+0.25 V vs. SCE), allowing for faster reaction to H_2_S via two-electron reduction. Within the compact nanoprobe **F1**^2+^-ANP, the fluorescence and ^1^O_2_ generation ability of MEH-PPV and NIR775 are each quenched by EM **F1**^2+^ due to efficient fluorescence resonance ET (FRET) from MEH-PPV and NIR775 to EM **F1**^2+^; the afterglow of **F1**^2+^-ANP is substantially quenched at the “off” state (Fig. 1b). In the presence of H_2_S, the EM **F1**^2+^ can be rapidly reduced into diene EM **F2** with distinct blue-shift absorption (*λ*_abs_ < 500 nm); the FRET process is hence eliminated, leading to recovery of ^1^O_2_ production in **F2**-ANP under 808-nm light irradiation. Therefore, NIR afterglow is activated at “on” state through ^1^O_2_-dependent oxidation of MEH-PPV, followed by an efficient intraparticle ET process. Ultralow background noise and deep-tissue penetration are achieved given advantages of long-lasting afterglow luminescence and rapid reaction kinetics. The probe thus presents a high SBR for rapid and sensitive detection of H_2_S levels in human blood samples, clinical liver tumor resections, and living mice.

### Optimization of EM 1^2+^ into EM F1^2+^

We first optimized EM **1**^2+^ into EM **F1**^2+^ by replacing the two phenyl rings with electron-withdrawing pentafluorophenyl rings (Fig. 1a). Density functional theory (DFT) calculation predicted that the introduction of two pentafluorophenyl groups would lead to an energetically lower-lying lowest unoccupied molecular orbital of **F1**^2+^ (−8.1 eV) than **1**^2+^ (−7.8 eV), which was validated by cyclic voltammetry (CV) analysis (Supplementary Fig. 1c). The *E*^red^ of **F1**^2+^ (+0.40 V vs. SCE) was higher than that of **1**^2+^ (+0.25 V vs. SCE), suggesting that **F1**^2+^ could act as a stronger oxidant toward H_2_S. High-performance liquid chromatography (HPLC) analysis showed that purple **F1**^2+^ could be efficiently reduced into light yellow diene **F2** by NaHS (H_2_S donor) in phosphate-buffered saline (PBS) buffer (pH 7.4), resulting in a noticeable blue shift of UV–visible–NIR absorption from 550 and 758 nm to 430 nm (Supplementary Fig. 2b, c). The second-order reaction rate (*k*_2_) between **F1**^2+^ and NaHS in PBS buffer (pH 7.4) was found to be 3600 ± 130 M^−1^ s^−1^, ~11.8-fold faster than that of **1**^2+^ (304 ± 8 M^−1^ s^−1^; *k*_2_ is defined as mean ± standard deviation from three independent measurements) (Supplementary Fig. 2d–i). Moreover, the introduction of electron-withdrawing groups contributed to a red shift of the NIR absorption band from 685 nm in **1**^2+^ to 758 nm in **F1**^2+^, causing extensive spectral overlap between **F1**^2+^ absorption and NIR775 emission (Supplementary Fig. 3a). Therefore, **F1**^2+^ quenched NIR775 fluorescence more efficiently (~1428-fold) than **1**^2+^ (~740-fold) (Supplementary Fig. 3). **F1**^2+^ was also highly stable following incubation in the PBS buffer (pH 7.4) for 7 days, and little photobleaching occurred upon irradiation with an 808-nm laser (1 W cm^−2^) for 3 min (Supplementary Fig. 4). These results indicate that **F1**^2+^ was a more efficient H_2_S-responsive chromophore than **1**^2+^.

### Preparation of F1^2+^-ANP

**Fig. 2:**
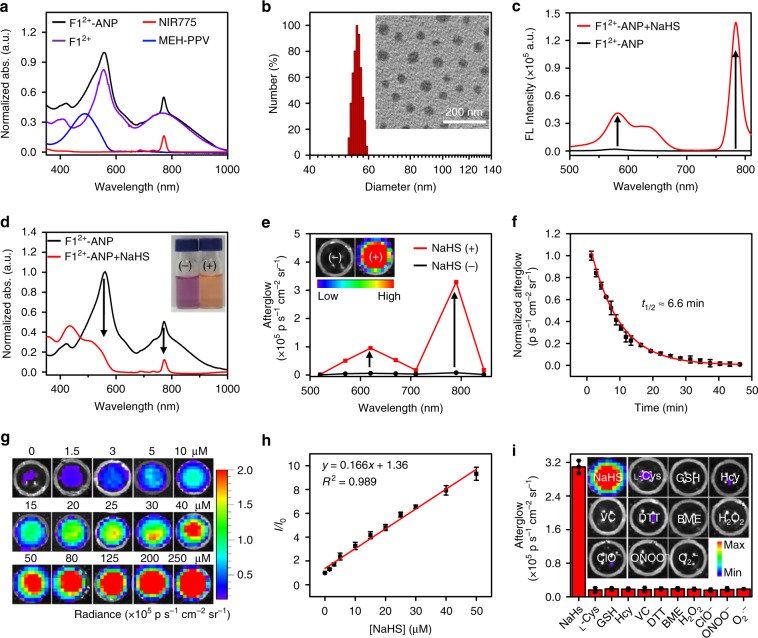
Characterization of **F1**^2+^-ANP in vitro. **a** Comparison of the UV–visible-NIR absorption spectra of **F1**^2+^-ANP, **F1**^2+^, NIR775, and MEH-PPV. **b** DLS and transmission electron microscopy (TEM) image (inset) of **F1**^2+^-ANP. **c** Fluorescence and **d** absorption spectra of **F1**^2+^-ANP (58/28/2.2 μg mL^−1^ **F1**^2+^(BF_4_^−^)_2_/MEH-PPV/NIR775) in the absence or presence of NaHS (200 μM, 1 min). Inset: photographs of **F1**^2+^-ANP before (−) and after (+) incubation with NaHS in PBS buffer. Fluorescence spectra was acquired by synchronous fluorescence scanning (*λ*_ex_ = 400‒800 nm, offset = 100 nm). **e** Afterglow luminescence spectra and images (inset) of **F1**^2+^-ANP (58/28/2.2 μg mL^−1^ **F1**^2+^(BF_4_^−^)_2_/MEH-PPV/NIR775) with and without incubation with NaHS (200 μM) in PBS buffer (pH 7.4) at 37 °C for 1 min, followed by irradiation with 808-nm laser (1 W cm^−2^, 1 min). After cessation of laser, the afterglow images were acquired under an open filter, with an acquisition time of 60 s. **f** Decay of afterglow luminescence of H_2_S-activated **F1**^2+^-ANP in PBS buffer at 37 °C. **g** Afterglow luminescence images of **F1**^2+^-ANP (58/28/2.2 μg mL^−1^ **F1**^2+^(BF_4_^−^)_2_/MEH-PPV/NIR775) upon incubation with varying concentrations of NaHS (0, 1.5, 3, 5, 10, 15, 20, 25, 30, 40, 50, 80, 125, 200, and 250 μM) at 37 °C for 1 min. **h** Plot of the afterglow luminescence intensity of **F1**^2+^-ANP and the concentration of NaHS from 0 to 50 μM. **i** Afterglow luminescence intensities and images (inset) of **F1**^2+^-ANP upon incubation with different reductants or ROS (200 µM NaHS, 1.25 mM l-cysteine (l-Cys), 10 mM glutathione (GSH), 1 mM homocysteine (Hcy), 1.25 mM ascorbic acid (VC), 1.25 mM dithiothreitol (DTT), 100 μM β-mercaptoethanol (BME), 1 mM H_2_O_2_, 1 mM ClO^−^, ONOO^−^ (1 mM NaNO_2_ + 1 mM H_2_O_2_), O_2_^.**−**^ (100 μM xanthine + 22 mU xanthine oxidase)) for 10 min. The solutions were then irradiated with the 808-nm laser (1 W cm^−2^, 1 min), and the NIR afterglow images were collected for 60 s with a 790 nm filter after the end of irradiation. Data denote mean ± standard deviation (s.d.) (*n* = 3). Source data are provided as a Source Data file.

We then employed **F1**^2+^ to prepare an H_2_S-activatable NIR afterglow luminescent probe (**F1**^2+^-ANP) by doping it into NIR775 and MEH-PPV-based afterglow SPNs via DSPE-PEG-assisted encapsulation (Fig. 1b). The encapsulating ratio of MEH-PPV, NIR775, and **F1**^2+^(BF_4_^−^)_2_ within **F1**^2+^-ANP was optimized at ~28/2.2/58 (by mass) (Supplementary Fig. 5), unveiling characteristic absorption bands of MEH-PPV, NIR775, and **F1**^2+^ (Fig. 2a). Dynamic light scattering (DLS) and transmission electron microscopy analysis revealed that **F1**^2+^-ANP appeared as mono-disperse nanoparticles, with a mean hydrodynamic size of ~55 nm (Fig. 2b). As expected, the fluorescence of MEH-PPV (580 nm) and NIR775 (780 nm) within **F1**^2+^-ANP was efficiently quenched by **F1**^2+^ owing to extensive spectral overlaps between their fluorescence emissions and **F1**^2+^ absorption (Fig. 2c). Fluorescence lifetime measurement revealed that the lifetimes of MEH-PPV and NIR775 within **F1**^2+^-ANP were dramatically shortened as compared with that in the absence of **F1**^2+^ (Supplementary Fig. 6), suggesting that efficient FRET processes occurred between MEH-PPV and **F1**^2+^, and NIR775 and **F1**^2+^, with ET efficiency estimated to be 97.1% and 99.9%, respectively (Supplementary Fig. 7 and Supplementary Note. 1). Upon incubation with NaHS (90 μM) in PBS buffer (pH 7.4) for 1 min, **F1**^2+^ absorption above 500 nm within **F1**^2+^-ANP nearly disappeared, and the solution color changed from violet to light orange (Fig. 2d). Accordingly, the initially quenched fluorescence at 580 and 780 nm were activated by H_2_S (Fig. 2c). The *k*_2_ between **F1**^2+^-ANP and H_2_S in PBS buffer (pH 7.4) was 1563 ± 141 M^−1^ s^−1^ (Supplementary Fig. 8), much faster than other reported optical probes amenable for in vivo imaging of H_2_S (Supplementary Dataset 1).

As ^1^O_2_ is indispensable in oxidizing MEH-PPV into PPV dioxetanes, which subsequently decompose into MEH-PPV-aldehyde and emit afterglow luminescence (Fig. 1c), the ^1^O_2_ generation ability of **F1**^2+^-ANP before and after reaction with NaHS was measured. Irradiation of **F1**^2+^-ANP with the 808-nm laser (1 W cm^−2^, 1 min) induced little enhancement in singlet oxygen sensor green (SOSG) fluorescence, indicating low ability to produce ^1^O_2_ (Supplementary Fig. 9). However, irradiation of **F1**^2+^-ANP after incubation with NaHS for 1 min produced remarkably enhanced SOSG fluorescence. Generated ^1^O_2_ could evoke MEH-PPV oxidation as evidenced by the gradually declining MEH-PPV absorption within activated **F1**^2+^-ANP (i.e., **F2**-ANP) upon irradiation with the 808-nm laser (1 W cm^−2^) for 0–60 min (Supplementary Fig. 10).

We next investigated the afterglow luminescence of **F1**^2+^-ANP in the absence or presence of NaHS in PBS buffer (pH 7.4) at 37 °C. After optimizing irradiation conditions (Supplementary Fig. 11), the solutions were pre-irradiated by the 808-nm laser at a power density of 1 W cm^−2^ for 1 min. **F1**^2+^-ANP exhibited little afterglow in the absence of NaHS, whereas NaHS-treated **F1**^2+^-ANP displayed obvious afterglow signals in the visible and NIR regions. Signal quantification from the images (Fig. 2e, inset) showed that afterglow intensities increased by ~122-fold. Notably, the afterglow signals in the NIR region were much higher than those in the visible region, presumably attributable to the ET process from MEH-PPV to NIR775 within activated **F1**^2+^-ANP as demonstrated by the spectral overlaps between them (Supplementary Fig. 12a, b). The ET process between MEH-PPV and NIR775 was further confirmed by the shorter fluorescence lifetime of MEH-PPV in activated **F1**^2+^-ANP (~49 ps) than that in the absence of NIR775 (~106 ps), with the ET efficiency of ~53.8% (Supplementary Fig. 12c and Supplementary Note. 2). These results suggest that H_2_S can effectively turn on the NIR afterglow luminescence of **F1**^2+^-ANP. Through continuous acquisition of afterglow images, the afterglow could persist for over 40 min after laser cessation, with a half-life of ~6.6 min (Fig. 2f and Supplementary Fig. 13). Moreover, the afterglow signal could be recharged by multiple irradiation with the 808-nm laser; the afterglow signal of activated **F1**^2+^-ANP was still ~50-fold higher than that of unactivated **F1**^2+^-ANP after irradiation for 15 cycles over 3 days, while their size and morphology were negligibly changed after irradiation, reflecting the potential for long-term imaging (Supplementary Figs. 14 and 15).

We subsequently examined the sensitivity and specificity of **F1**^2+^-ANP toward H_2_S (Fig. 2g, h). Afterglow images of **F1**^2+^-ANP became brighter with NaHS concentration, showing a linear correlation between afterglow intensities and NaHS concentrations from 0 to 50 μM (limit of detection: ~0.1 μM (3*σ*/*k*). Only H_2_S could turn on the afterglow of **F1**^2+^-ANP (Fig. 2i), consistent with those measured by fluorescence (Supplementary Fig. 16), demonstrating that **F1**^2+^-ANP was a specific probe for H_2_S. In addition, **F1**^2+^-ANP was highly stable under different physiological conditions (Supplementary Figs. 17 and 18); the fluorescence and afterglow of **F1**^2+^-ANP were similarly activated by NaHS in different cell culture mediums (Supplementary Fig. 19) or in PBS buffers with pH ranging from 5.0 to 8.0 (Supplementary Fig. 20). Taking advantage of high-sensitivity, specificity, and ultrafast reaction kinetics toward H_2_S, **F1**^2+^-ANP was applied to quantify H_2_S concentration in mouse blood (Supplementary Fig. 21). The H_2_S concentration in mouse whole blood was measured to be 24.8 ± 2.2 μM (the concentration is expressed as mean ± standard deviation of three samples) using NaHS as an internal standard, which was within reported values^35,48,49^.

### Detection of H_2_S in HCC cells

**Fig. 3:**
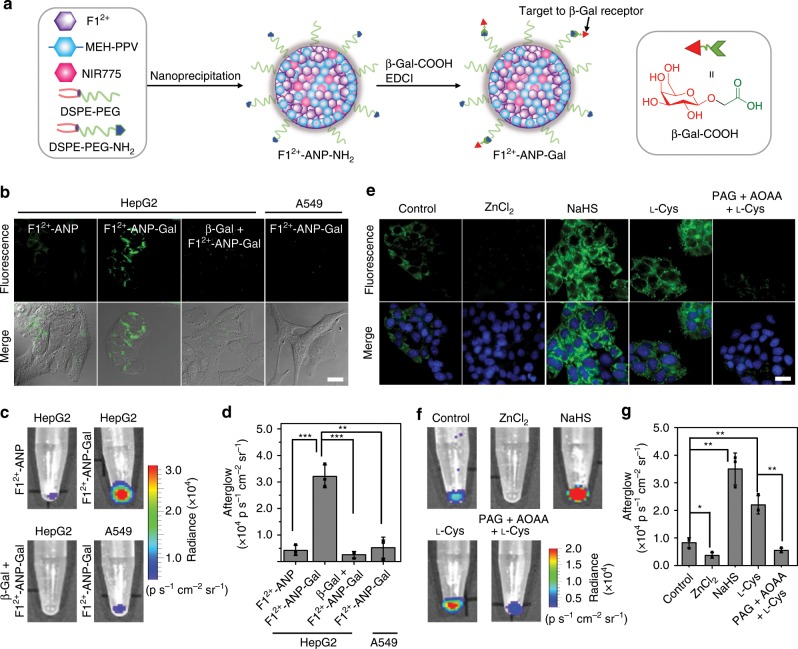
Preparation of **F1**^2+^-ANP-Gal for imaging of H_2_S in cells. **a** Schematic for the preparation of **F1**^2+^-ANP-Gal. **b** Fluorescence imaging of HepG2 cells after incubation with **F1**^2+^-ANP, **F1**^2+^-ANP-Gal, **F1**^2+^-ANP-Gal plus 20 mM free β-Gal, or A549 cells incubated with **F1**^2+^-ANP-Gal for 3 h. Scale bar: 20 μm. **c** Afterglow luminescence images and intensities (**d**) of cells (~1 × 10^5^ cells) treated with indicated conditions in **b**. **e** Fluorescence imaging of HepG2 cells incubated with **F1**^2+^-ANP-Gal, **F1**^2+^-ANP-Gal together with 300 µM ZnCl_2_, 1 mM NaHS, 200 μM l-Cys, or 200 μM l-Cys plus 50 mg L^−1^ PAG and 20 μM AOAA. Scale bar: 20 μm. **f** Afterglow luminescence images and intensities **g** of HepG2 cells (~3 × 10^4^ cells) treated with indicated conditions in **e**. All the cells were incubated with **F1**^2+^-ANP or **F1**^2+^-ANP-Gal at a concentration of 58/28/2.2 μg mL^−1^ **F1**^2+^(BF_4_^−^)_2_/MEH-PPV/NIR775 for 3 h. For afterglow luminescence imaging, the cell pellets were irradiated with the 808-nm laser (1 W cm^−2^) for 1 min. After cessation of laser, the afterglow images were acquired for 60 s with an open filter. Data denote mean ± s.d. (*P < 0.05, **P < 0.01, ***P < 0.001, *n* = 3). Statistical differences were analyzed by Student’s *t* test. Source data are provided as a Source Data file.

Since increased H_2_S level is found in malignant liver tumors^50,51^, we investigated the potential to detect H_2_S in human HCC HepG2 cells through afterglow imaging. The overexpression of cystathionine β-synthase (CBS) and cystathionine γ-lyase (CSE) for the biosynthesis of endogenous H_2_S in HepG2 cells was validated by Western Blot (WB) analysis (Supplementary Fig. 22). To enable efficient entry into HepG2 cells, β-Gal capable of targeting to β-Gal receptors overexpressed on HepG2 cells was introduced to the surface of **F1**^2+^-ANP to prepare **F1**^2+^-ANP-Gal (Fig. 3a). Akin to **F1**^2+^-ANP, **F1**^2+^-ANP-Gal manifested as mono-disperse nanoparticles in PBS buffer (pH 7.4) and exhibited significantly activated afterglow in response to H_2_S (Supplementary Fig. 23). **F1**^2+^-ANP-Gal demonstrated low toxicity to HepG2 cells by the 3-(4,5-dimethylthiazol-2-yl)-2,5-diphenyltetrazolium bromide (MTT) assay, with little cell death observed before and after irradiation with the 808-nm laser (1 W cm^−2^, 1 min) (Supplementary Fig. 24). After optimizing incubation conditions (Supplementary Figs. 25 and 26), **F1**^2+^-ANP-Gal could be taken up by HepG2 cells via β-Gal receptor-mediated endocytosis (Fig. 3b), resulting in intracellular fluorescence distributed mainly in lysosomes and mitochondria (Supplementary Fig. 27). We then collected cell pellets (~1 × 10^5^ cells) and irradiated with the 808-nm laser (1 W cm^−2^, 1 min) to produce afterglow. As presented in Fig. 3c, HepG2 cells incubated with **F1**^2+^-ANP-Gal showed a much brighter afterglow image compared to that of **F1**^2+^-ANP, β-Gal-pre-treated HepG2 cells, or β-Gal receptor-deficient human lung cancer A549 cells incubated with **F1**^2+^-ANP-Gal. The afterglow intensity in HepG2 cells incubated with **F1**^2+^-ANP-Gal was more than 7-fold higher than that with **F1**^2+^-ANP (Fig. 3d). We subsequently applied **F1**^2+^-ANP-Gal to monitor H_2_S fluctuation in HepG2 cells (Fig. 3f, g). Afterglow signals in **F1**^2+^-ANP-Gal-treated HepG2 cell pellets (~3 × 10^4^ cells) increased noticeably when pre-treated with extraneous NaHS; pre-treatment with ZnCl_2_ to scavenge H_2_S reduced afterglow substantially. HepG2 cells stimulated with l-Cys (a precursor for biosynthesis of H_2_S) to upregulate intracellular H_2_S levels could augment afterglow, which was greatly inhibited when adding dl-propargylglycine (PAG; 50 μg mL^−1^) and aminooxyacetic acid (AOAA; 20 μM) to inhibit endogenous CSE and CBS activities. These results were confirmed by fluorescence imaging of H_2_S in individual HepG2 cells (Fig. 3e). We also found that afterglow signals in **F1**^2+^-ANP-Gal-treated HepG2 cell pellets increased with the cell number, which could allow to detect 1000 and 5000 HepG2 cells in vitro and in vivo, respectively, much lower than fluorescence (Supplementary Figs. 28 and 29). Moreover, the afterglow luminescence of **F1**^2+^-ANP-Gal could still be clearly observed in HepG2 cells even after irradiation for 12 cycles over 24 h, suggesting the potential for long-term afterglow imaging in live HepG2 cells (Supplementary Fig. 30). Thus, **F1**^2+^-ANP-Gal was an efficient probe capable of sensitively detecting HepG2 cells through afterglow imaging of endogenous H_2_S.

### Afterglow imaging of H_2_S in subcutaneous liver tumors

Prior to afterglow imaging of H_2_S in vivo, the penetration depth of afterglow luminescence emitted from the H_2_S-activated **F1**^2+^-ANP-Gal was examined. The afterglow and fluorescence signals each declined with the thickness of chicken tissues (Supplementary Fig. 31). However, because tissue autofluorescence was minimized without real-time excitation, the afterglow exerted significantly higher SBR than NIR fluorescence. At a thickness of ~4 cm, the SBR of afterglow remained as high as 18.7 ± 4.6 (SBR is expressed as mean ± standard deviation, *n* = 3), whereas the SBR of NIR fluorescence was close to 1. These results indicate that the penetration of afterglow luminescence was much deeper than that of NIR fluorescence, which was also confirmed by the larger SBR of afterglow (~201) versus fluorescence (~2.5) when detecting signals through a mouse body (~1.9 cm). We next determined the blood half-life (*t*_1/2_) of **F1**^2+^-ANP-Gal following intravenous (i.v.) injection into mice. The *t*_1/2_ was measured to be ~7.5 h, permitting efficient circulation to facilitate delivery into tumor tissues. The biodistribution study showed that **F1**^2+^-ANP-Gal accumulated mainly in the liver of healthy mice at 12 h post injection (%ID/g ≈ 23.6%, Supplementary Fig. 32).

**Fig. 4:**
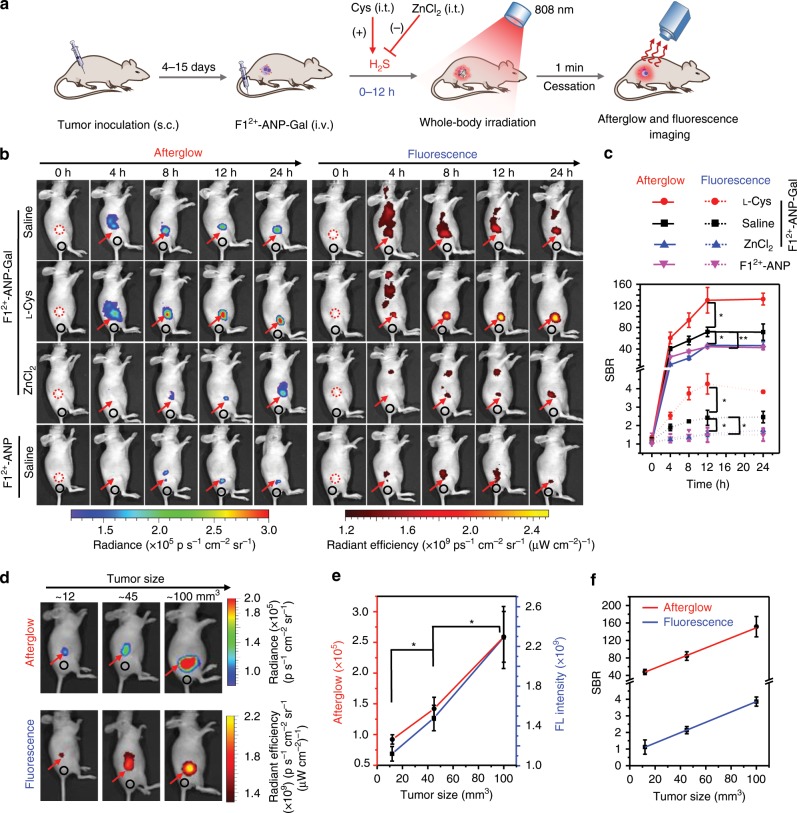
Afterglow imaging of H_2_S in s.c. HepG2 tumors. **a** Schematic for noninvasive fluorescence and afterglow imaging of H_2_S in HepG2 tumor-bearing mice. **b** Afterglow (left) and fluorescence (right) imaging of HepG2 tumors in mice at 0, 4, 8, 12, and 24 h following i.v. injection of **F1**^2+^-ANP-Gal or **F1**^2+^-ANP in saline (saline), **F1**^2+^-ANP-Gal with i.t. injection of l-Cys or ZnCl_2_. l-Cys (1 mM, 25 µL) or ZnCl_2_ (1 mM, 25 µL) was injected into tumors at 3.5 h post i.v. injection of **F1**^2+^-ANP-Gal (211/100/8 μg **F1**^2+^(BF_4_^−^)_2_/MEH-PPV/NIR775, 200 μL). **c** Quantification of SBRs for afterglow (solid) and fluorescence (dash) imaging of HepG2 tumors in mice treated with **F1**^2+^-ANP-Gal or **F1**^2+^-ANP alone, **F1**^2+^-ANP-Gal plus l-Cys, or ZnCl_2_ at indicated time point. **d** Afterglow (up) and fluorescence (down) imaging of HepG2 tumors at size of ~12, ~45, and ~100 mm^3^ in mice at 12 h post i.v. injection of **F1**^2+^-ANP-Gal. **e** Quantification of afterglow and fluorescence intensities of HepG2 tumors at different size. **f** Plots of the SBRs for afterglow and fluorescence imaging versus the tumor size revealed a strong correlation for **F1**^2+^-ANP-Gal (Person’s *r* = 0.99). For afterglow imaging, the mouse body was irradiated with the 808-nm laser (1 W cm^−2^) for 1 min. After cessation of laser, the afterglow images were acquired for 60 s with an open filter. The fluorescence images were acquired with *λ*_ex/em_ = 740/790 nm. Red arrows indicate the locations of HepG2 tumors in mice, and black circles indicate the background locations. Data denote mean ± s.d. (*P < 0.05, **P < 0.01, *n* = 3). Statistical differences were analyzed by Student’s *t* test. Source data are provided as a Source Data file.

We next applied **F1**^2+^-ANP-Gal for afterglow imaging of endogenous H_2_S in subcutaneous (s.c.) HepG2 tumors (Fig. 4a). After i.v. injection into mice, afterglow and fluorescence signals both increased gradually and peaked after 12 h (Fig. 4b and Supplementary Fig. 33). However, owing to the low background signal of afterglow, the SBR of afterglow was much higher than that of fluorescence (Fig. 4c). At 12 h, the average SBR of afterglow was 71.9 ± 8.8, ~30-fold higher than that of fluorescence (2.4 ± 0.4). When l-Cys was injected into tumors to upregulate H_2_S levels, afterglow and fluorescence signals were both significantly enhanced, whereas intratumoral (i.t.) injection of ZnCl_2_ to scavenge tumor H_2_S reduced tumor afterglow and fluorescence. The SBR of afterglow 12 h post injection of **F1**^2+^-ANP-Gal and l-Cys increased to 133.8 ± 24.9, ~1.9- and ~2.9-fold higher than that of **F1**^2+^-ANP-Gal alone and **F1**^2+^-ANP-Gal plus ZnCl_2_ (46.7 ± 1.6), respectively. In addition, mice injected with untargeted **F1**^2+^-ANP showed much lower afterglow signals and SBR (44.2 ± 3.1) in HepG2 tumors compared to **F1**^2+^-ANP-Gal. These results aligned with ex vivo fluorescence imaging of resected tumor tissue slices (Supplementary Fig. 34), affirming that **F1**^2+^-ANP-Gal could deliver into HepG2 tumors and efficiently detect tumor H_2_S levels.

We applied **F1**^2+^-ANP-Gal to noninvasively monitor HepG2 tumor growth in living mice. After s.c. injection of HepG2 cells (2 × 10^6^) at ~4, ~8, and ~15 days, average tumor sizes grew to ~12 (~2.8 × 2.9 mm^2^), ~45 (~4.4 × 4.5 mm^2^), and ~100 mm^3^ (~5.7 × 6.1 mm^2^), respectively. The afterglow and fluorescence signals of HepG2 tumors 12 h after i.v. injection of **F1**^2+^-ANP-Gal increased with tumor size, and their SBRs paralleled those of tumor size (Fig. 4d, e). For a tumor measuring only ~12 mm^3^, the SBR of afterglow was 47.8 ± 5.9; conversely, the SBR of fluorescence was only 1.1 ± 0.4 (Fig. 4f). These results indicate that the afterglow emitted from **F1**^2+^-ANP-Gal after activation by endogenous H_2_S was more appropriate than fluorescence when applied to detect tiny s.c. HepG2 tumors in vivo. Notably, we identified a strong correlation between the SBR of afterglow or fluorescence produced from **F1**^2+^-ANP-Gal and apparent tumor size growth (Pearson’s *r* = 0.99). Therefore, **F1**^2+^-ANP-Gal can offer valuable information about tumor growth in living mice.

### Imaging of orthotopic liver tumors in mice

**Fig. 5:**
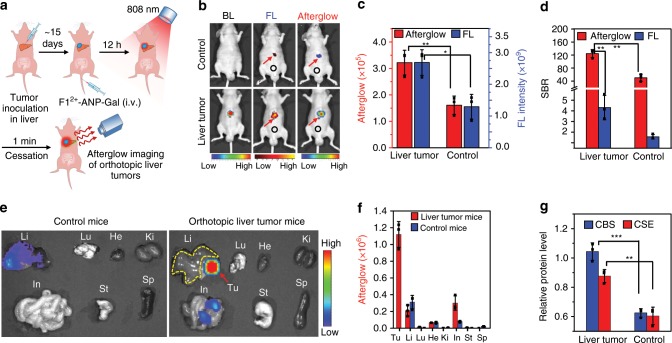
Noninvasive imaging of orthotopic liver tumors in mice. **a** Schematic for afterglow imaging of orthotopic HepG2 tumors in living mice. **b** Bioluminescence (BL), fluorescence (FL), and afterglow imaging of control mice (Control) and orthotopic HepG2 tumor (liver tumor) bearing mice at 12 h post i.v. injection of **F1**^2+^-ANP-Gal (211/100/8 μg **F1**^2+^(BF_4_^−^)_2_/MEH-PPV/NIR775, 200 μL). **c** Afterglow (red) and FL (blue) intensities in the livers of control mice and orthotopic liver tumor-bearing mice. **d** Comparison of the SBRs for afterglow and fluorescence imaging of livers in control mice and orthotopic liver tumor-bearing mice. Red arrows indicate the locations of livers, and black circles indicate the background locations. **e** Representative ex vivo afterglow images of main organs (e.g., liver (Li), lung (Lu), heart (He), kidneys (Ki), intestines (In), stomach (St), spleen (Sp), and tumor (Tu)) resected from control mice (left) and orthotopic HepG2 tumor-bearing mice (right) at 12 h post i.v. injection of **F1**^2+^-ANP-Gal. Red arrow and yellow dash box indicate the locations of HepG2 tumor in the liver and normal liver tissues chosen for region of interest (ROI), respectively. **f** Comparison of the average afterglow intensities of tumors and main organs resected from control (blue) and orthotopic liver tumor (red) mice. **g** WB analysis shows the relative CBS and CSE protein levels in the liver tissues resected from control mice and HepG2 tumor tissues resected from orthotopic liver tumor mice. All afterglow luminescence images were acquired for 60 s with an open filter, after pre-irradiation of mouse body or organs with the 808-nm laser (1 W cm^−2^, 1 min). All fluorescence images were acquired with *λ*_ex/em_ = 740/790 nm. Data denote mean ± s.d. (*P < 0.05, **P < 0.01, *n* = 3). Statistical differences were analyzed by Student’s *t* test. Source data are provided as a Source Data file.

By virtue of the deep-tissue penetration of afterglow imaging, **F1**^2+^-ANP-Gal was applied to detect orthotopic HepG2 liver tumors through afterglow imaging of H_2_S. Orthotopic liver tumors were established by inoculation of luciferase-transfected HepG2 cells (HepG2/Luc) into the lube of livers of mice (Fig. 5a). Strong bioluminescence (BL) in liver indicated the successful establishment of orthotopic liver tumors after 15 days (Fig. 5b). We then injected (i.v.) **F1**^2+^-ANP-Gal into mice followed by irradiation of each mice body (face up) with the 808-nm laser (1 W cm^−2^, 1 min) after 12 h (Fig. 5a). NIR fluorescence and afterglow images both showed that mice with orthotopic liver tumors had brighter signals relative to control mice (Fig. 5b, c), with locations closely matching those of BL images. The SBR of afterglow in orthotopic liver tumors was 124.5 ± 12.5, 2.5-fold higher than that in livers of control mice (50.0 ± 10.9) (Fig. 5d). The SBR of afterglow was also much higher compared to that of fluorescence in liver tumors (4.3 ± 1.2). We subsequently conducted ex vivo imaging of main organs (e.g., liver, lung, heart, kidneys, intestines, stomach, and spleen) resected from control mice and orthotopic liver tumor mice. Figure 5e shows that tumor tissues in the resected liver exhibited the brightest afterglow image among all resected organs. The afterglow intensity in tumor tissues was ~5.3-fold higher than that in surrounding liver tissues and ~3.6-fold higher than normal liver tissues of control mice (Fig. 5f). NIR fluorescence imaging of the same tissues verified these findings (Supplementary Fig. 35), which were further confirmed via fluorescence imaging of tumor and normal tissue slices (Supplementary Fig. 36). Subsequent WB analysis revealed that enhanced afterglow and fluorescence in orthotopic HepG2/Luc tumors concurred with the higher expression of CBS and CSE enzymes compared to normal liver tissues (Fig. 5g and Supplementary Fig. 37). These findings demonstrate that **F1**^2+^-ANP-Gal could offer a large SBR to accurately and noninvasively position orthotopic liver tumor foci via afterglow imaging of H_2_S.

### Detection of H_2_S in clinical blood samples

**Fig. 6:**
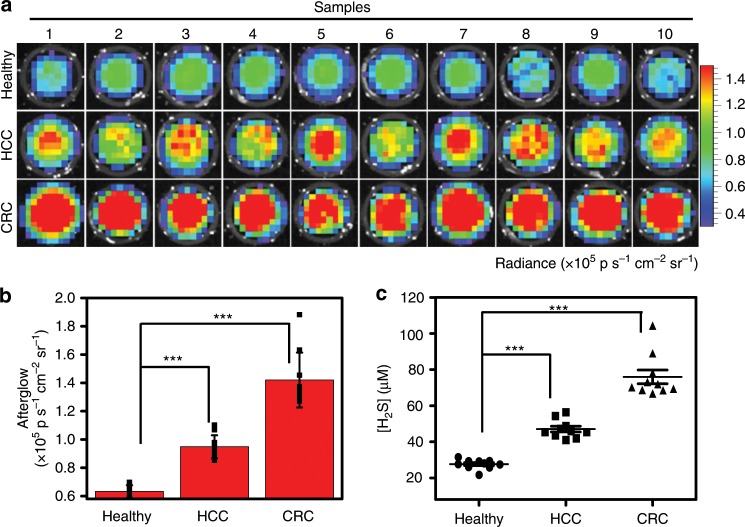
Detection of H_2_S in blood samples. **a** Afterglow images of **F1**^2+^-ANP in blood samples freshly collected from 10 healthy persons, 10 HCC patients, and 10 CRC patients. Freshly collected bloods were 2-fold diluted with PBS buffer (1×, pH 7.4), and then incubated with **F1**^2+^-ANP (58/28/2.2 μg mL^−1^ **F1**^2+^(BF_4_^−^)_2_/MEH-PPV/NIR775) at 37 °C for 1 min, followed by irradiation with the 808-nm laser (1 W cm^−2^) for 1 min. After removal of the laser, the afterglow luminescence images were immediately acquired for 60 s with an 790 nm filter. **b** Afterglow luminescence intensities of **F1**^2+^-ANP in 2-fold diluted blood samples. **c** Quantification of H_2_S concentrations in whole blood of healthy persons and HCC or CRC patients. Data denote mean ± s.d. (***P < 0.001, *n* = 10). Statistical differences were analyzed by Student’s *t* test. Source data are provided as a Source Data file.

We employed the activatable afterglow to detect H_2_S levels in human blood samples collected from 10 healthy persons, 10 HCC patients, and 10 CRC patients in clinics. Following incubation of 2-fold diluted blood with **F1**^2+^-ANP for 1 min, afterglow signals in HCC and CRC patients’ bloods were much higher than that in healthy persons (Fig. 6a, b). We took a standard curve established by the addition of NaHS into healthy persons’ blood as the internal standard, and the average H_2_S level in the whole blood of healthy persons was 27.6 ± 2.7 μM (the H_2_S level is expressed as mean ± standard deviation, *n* = 10); the level increased to 47.0 ± 5.0 and 75.9 ± 11.9 μM in the whole blood of HCC and CRC patients, respectively (Fig. 6c and Supplementary Fig. 38). These results revealed that blood H_2_S levels in HCC and CRC patients were upregulated compared to healthy persons, suggesting that H_2_S could serve as a potential blood marker for certain malignant cancers (e.g., HCC and CRC). Thus, **F1**^2+^-ANP presents high potential to quantify blood H_2_S levels for cancer diagnosis.

### Detection of liver tumor tissues in clinical specimens

**Fig. 7:**
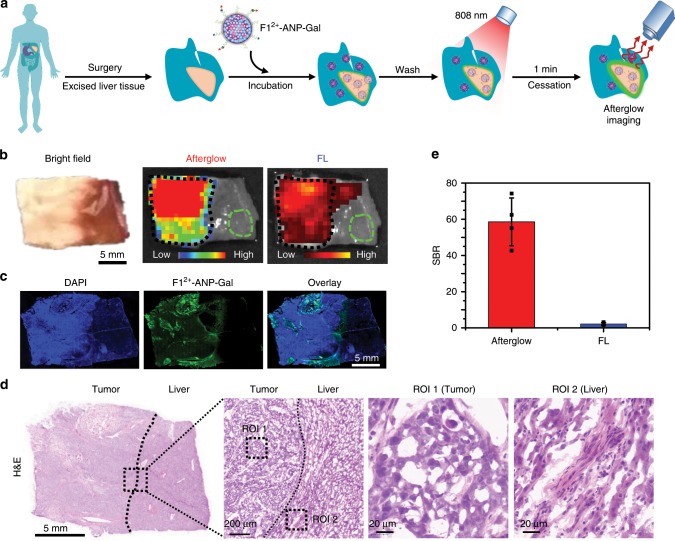
Afterglow imaging of liver tumor tissues in HCC specimens. **a** Schematic for afterglow imaging of tumor tissues in clinically excised liver specimens using **F1**^2+^-ANP-Gal. **b** Representative photograph (bright field), afterglow, and FL images of the liver specimen resected from an HCC patient. The specimen was incubated with **F1**^2+^-ANP-Gal (58/28/2.2 μg mL^−1^ **F1**^2+^(BF_4_^−^)_2_/MEH-PPV/NIR775) in PBS buffer (1×, pH 7.4) at 37 °C for 3 h, and then rinsed with PBS buffer for three times. The whole specimen was irradiated with the 808-nm laser (1 W cm^−2^, 1 min). After cessation of the laser, the afterglow image was acquired under an open filter, with an acquisition time of 60 s. The fluorescence image was collected with *λ*_ex/em_ = 740/790 nm. **c** Fluorescence images of liver tissue slices were dissected from the HCC specimen after incubation with **F1**^2+^-ANP-Gal (green) for 3 h and stained with DAPI (blue). **d** H&E staining of the liver tissue slice dissected from the HCC specimen. Black dash boxes indicate the enlarged areas, in which box ROI 1 shows the tumor tissue and box ROI 2 indicates the normal liver tissue, respectively. **e** Quantitative analysis of the average SBRs for afterglow and fluorescence imaging of liver specimens resected from HCC patients. Data denote mean ± s.d. (*n* = 4). Black dash boxes in **b** indicate the location of tumors, and green dash boxes indicate the location of normal liver tissues selected as the background. Source data are provided as a Source Data file.

Having demonstrated the higher blood H_2_S levels in HCC patients’ bloods, we employed afterglow to delineate tumor margins in clinically excised HCC specimens. Excised liver tissues from four HCC patients were incubated with **F1**^2+^-ANP-Gal in PBS buffer (pH 7.4) for 3 h to allow efficient internalization and activation by H_2_S. After being washed with PBS buffer, whole specimens were irradiated with the 808-nm laser (1 W cm^−2^, 1 min); the resulting afterglow and fluorescence images were acquired immediately (Fig. 7a). A strong afterglow image appeared in the left section of the specimen, corroborating the NIR fluorescence image of the tissue slice (Fig. 7b, c). Subsequent hematoxylin and eosin (H&E) staining revealed that the left section of the specimen was characterized by tumor tissue (Fig. 7d). Lesions with clear afterglow and fluorescence signals resulting from **F1**^2+^-ANP-Gal staining accorded with the tumor location was indicated by H&E staining, as was observed in the other three specimens from different HCC patients (Supplementary Figs. 39–41). These results suggest that **F1**^2+^-ANP-Gal was effective in delineating liver tumor lesions in clinical specimens. Upon dividing the signal intensity in tumor lesions by that in adjacent normal liver tissue, the average SBR in afterglow imaging of the four clinical specimens was 58.6 ± 13.2, significantly higher than that in fluorescence (2.2 ± 0.7) (Fig. 7e). Such a high afterglow SBR could promote detection of tumor lesions in the specimen covered with 1.5-cm-thick chicken tissue (Supplementary Fig. 42). These findings imply that **F1**^2+^-ANP-Gal may have promise for detecting deeply located tumor cells, which could be applied to identify tumor margins and accurately guide liver tumor surgery in the future.

### In vivo clearance and biosafety evaluations

To evaluate the in vivo clearance and biosafety, we injected (i.v.) **F1**^2+^-ANP-Gal at the same dose as the imaging dose (211/100/8 μg **F1**^2+^(BF_4_^−^)_2_/MEH-PPV/NIR775, 200 μL). The NIR fluorescence images showed that the NIR fluorescence was gradually observed in the liver, with intensity maximized at 12 h (Supplementary Fig. 43). After that, the NIR fluorescence in mice decreased overtime, and nearly returned to the background after 28 days, indicating that **F1**^2+^-ANP-Gal could be cleared out after systemic administration. We then monitored the body weights, showing little difference between **F1**^2+^-ANP-Gal-treated and saline-treated mice for 28 days (Supplementary Fig. 44a). Moreover, the subsequent biochemistry and hematology test of mice 1, 7, 14, and 28 days post injection of **F1**^2+^-ANP-Gal showed that all the markers related to liver or kidney functions were within the reference range of healthy mice (Supplementary Fig. 44b, c); **F1**^2+^-ANP-Gal did not cause obvious toxic effect on mice, which was further confirmed by the histologic analysis of resected main organs (e.g., liver, lung, kidney, heart, and spleen) (Supplementary Fig. 45).

## Discussion

Afterglow luminescence holds great promise for sensitive and noninvasive imaging of biomolecules in living subjects^26,31^; however, real-time afterglow imaging remains rare due to lack of afterglow probes that can be rapidly activated by a molecular target to produce intense afterglow luminescence. Herein, we optimized the structure of an organic π-electron system-based EM **1**^2+^ into EM **F1**^2+^ and engineered it as an effective H_2_S-responsive chromophore capable of integrating with NIR775 and MEH-PPV to develop H_2_S-activatable NIR afterglow probe (**F1**^2+^-ANP) for in vivo imaging. **F1**^2+^ with two electron-withdrawing pentafluorophenyl groups showed strong absorptions at 550 and 758 nm, overlapping more extensively with MEH-PPV (580 nm) and NIR775 emissions (780 nm) than **1**^2+^. **F1**^2+^ also possessed a more positive value of *E*^red^ (+0.40 V vs. SCE) than **1**^2+^ (+0.25 V vs. SCE), permitting much faster reaction kinetics toward H_2_S (*k*_2_ = 3600 ± 130 M^−1^ s^−1^). These features offer **F1**^2+^ noteworthy advantages over **1**^2+^ in designing activatable afterglow probes. The longer absorption of **F1**^2+^ could (a) allow it to prevent NIR775 from generating ^1^O_2_, which is key to eliciting afterglow luminescence (Supplementary Fig. 9) and (b) directly quench visible and NIR afterglow luminescence of **F1**^2+^-ANP. Accordingly, afterglow signals could be substantially quenched in an “off” state. Upon reaction with H_2_S, rapid reaction kinetics between **F1**^2+^ and H_2_S could allow H_2_S to efficiently reduce **F1**^2+^ into **F2** within 1 min, thereby recovering afterglow luminescence by a factor of ~122-fold (Fig. 2e). Such activatable afterglow probes with large turn-on ratio and ultrafast reaction kinetics could facilitate sensitive and real-time detection of H_2_S in vitro and in vivo.

H_2_S is recognized as the third signaling gasotransmitter, participating in many essential physiological and pathological processes^36–39^. Throughout the past few years, many colorimetric and fluorescent probes have been developed for H_2_S measurement^52–57^. However, due to the low concentration and transient nature of H_2_S in biology^58^, the detection of endogenous H_2_S with these probes has been compromised by insufficient sensitivity, selectivity, or reaction kinetics. **F1**^2+^-ANP possesses high sensitivity, selectivity, and an ultrafast response to H_2_S, rendering it suitable for quantification of H_2_S concentration in mouse blood samples (Supplementary Fig. 21). Parallel measurements of H_2_S levels in human blood freshly collected from healthy persons, HCC patients, and CRC patients were taken following incubation with **F1**^2+^-ANP for 1 min. Findings showed that blood H_2_S levels in HCC and CRC patients were substantially higher than in healthy persons (Fig. 6), suggesting that H_2_S could be a potential blood marker for cancer diagnosis.

In addition to detecting blood H_2_S in vitro, **F1**^2+^-ANP was engineered into a hepatic tumor-targeting and H_2_S-activatable afterglow luminescent probe, **F1**^2+^-ANP-Gal, for afterglow imaging of hepatic tumor H_2_S in vivo. With a long blood circulation half-life and β-Gal receptor-mediated active delivery, **F1**^2+^-ANP-Gal could efficiently enter s.c. HepG2 tumors after i.v. injection into mice, turning on afterglow luminescence for real-time and noninvasive monitoring of H_2_S fluctuation in tumors. Benefiting from low tissue autofluorescence in the absence of real-time light excitation, **F1**^2+^-ANP-Gal with activated NIR afterglow presented a much higher SBR (47.8 ± 5.9) than NIR fluorescence (1.1 ± 0.4), permitting greater sensitivity to detect tiny s.c. HepG2 tumors (~12 mm^3^, Fig. 4d, f). The SBR resulting from afterglow images also increased linearly with tumor size, suggesting the potential of **F1**^2+^-ANP-Gal for (a) accurate detection of early-stage liver tumors and (b) noninvasive monitoring of HepG2 tumor growth in living mice through afterglow imaging of H_2_S. Moreover, the enhanced penetration depth of NIR afterglow luminescence could allow **F1**^2+^-ANP-Gal to sensitively detect orthotopic liver tumors, affording a large SBR (124.5 ± 12.5) to precisely position liver tumor foci in mice (Fig. 5). Of note, the H_2_S-induced rapid and substantial afterglow enhancement in **F1**^2+^-ANP-Gal could also offer a large SBR (58.6 ± 13.2) to identify liver tumor lesions in excised HCC specimens, with tumor margins aligning with H&E staining (Fig. 7). These results may offer valuable information to guide surgical resection of liver tumor tissues in intraoperative HCC patients.

In conclusion, we report the design and development of H_2_S-activatable NIR afterglow luminescent probes by doping EM **F1**^2+^ and NIR775 into MEH-PPV-based organic afterglow nanoparticles. Our findings demonstrate the probe’s utility in noninvasive and real-time imaging of H_2_S in living mice. **F1**^2+^-ANP can be selectively and rapidly activated by H_2_S, leading to a large afterglow luminescence turn-on ratio (~122-fold) after pre-irradiation with an 808-nm laser, which is useful for quantifying the H_2_S concentration in the blood of healthy persons (27.6 ± 2.7 μM), HCC patients (47.0 ± 5.0 μM), and CRC patients (75.9 ± 11.9 μM). A hepatic tumor-targeting and H_2_S-activatable afterglow probe (**F1**^2+^-ANP-Gal) is also built, offering a high SBR and deep-tissue penetration for high-sensitivity imaging of s.c. and orthotopic liver tumors via afterglow imaging of H_2_S in mice. Moreover, **F1**^2+^-ANP-Gal can accurately delineate liver tumor margins in excised HCC specimens, which may be especially useful for image-guided HCC surgery. This study presents the potential of using H_2_S-activatable afterglow luminescent probes for in vivo imaging, which could detect H_2_S levels in other diseases (e.g., liver inflammation and hypertension) and promote early disease diagnosis in the future.

## Methods

### Chemicals and characterization

^1^H and ^13^C nuclear magnetic resonance (NMR) spectra were recorded on a BRUKER Ascend^TM^ 400 (^1^H/400 MHz and ^13^C/100 MHz) spectrometer. Mass spectra were recorded on a JEOL JMS-T100GCV spectrometer in FD mode (GC-MS&NMR Laboratory, Research Faculty of Agriculture, Hokkaido University). Redox potentials (*E*^ox^ and *E*^red^) were measured by CV in dry CH_2_Cl_2_ containing 0.1 M Bu_4_NBF_4_ as a supporting electrolyte at a scan rate of 100 mV s^–1^. A Pt disk and wire electrodes were used as the working and counter electrodes, respectively. The working electrode was polished using a water suspension Al_2_O_3_ (0.05 µmol) before use. Column chromatography was performed on a silica gel I-6-40 (YMC, 40–63 µm), a silica gel 60 N (Kanto Chemical Co., Inc., spherical neutral, 40–50 µm), or aluminum oxide 90 standardized (Merck, 63–200 µm). The DFT calculations were performed with the Gaussian 09 W program package. The geometries of the compounds were optimized by using the B3LYP method in combination with the 6-31G* basis set, unless otherwise indicated. Melting points were measured on a Yamato MP-21 melting point apparatus and are recorded uncorrected. Elemental analyses were performed on an Exeter Analytical CE440 (the Open Facility, Global Facility Center, Creative Research Institution, Hokkaido University). HPLC analysis was carried out on Thermo Fisher U3000 HPLC systems. Absorption spectra were performed an Ocean Optics UV–visible spectrometer. Fluorescence spectra were obtained on a HORIBA Jobin Yvon Fluoromax-4 fluorescence spectrometer. An Olympus IX73 fluorescent inverted microscope was used for fluorescence imaging for cells and tissue slices. DLS and zeta potential analysis were measured by a 90 Plus/BI-MAS equipment (Brookhaven, USA). Transmission electron microscopy images were conducted on a JEM-1011 transmission electron microscope (JEOL Ltd., Japan) with an accelerating voltage of 100 kV. MTT assay was performed on a microplate reader (Tcan). The afterglow luminescence spectra, afterglow, and fluorescence images in vitro or vivo were acquired with the IVIS Lumina XR III imaging system. An 808-nm high-power NIR laser (FC-808-10W-MM, Xilong Company, China) was used to irradiate the specified irradiation sites to generate the afterglow luminescence, unless otherwise noted.

### Synthesis of F1^2+^(BF_4_^−^)_2_

The synthesis of **F1**^2+^(BF_4_^−^)_2_ was depicted in Supplementary Fig. 46 and Supplementary Methods. The NMR spectra and single crystal analysis of **F1**^2+^(BF_4_^−^)_2_ were summarized in Supplementary Figs. 47–53.

### Preparation of F**1**^2+^-ANP and F**1**^2+^-ANP-Gal

All the nanoparticles were prepared using amphiphilic polymers-assisted nanoprecipitation method. Typically, to prepare **F1**^2+^-ANP, **F1**^2+^(BF_4_^−^)_2_ (0.52 mg), MEH-PPV (0.25 mg), NIR775 (0.02 mg), and DSPE-PEG_2000_ (10 mg) were dissolved in tetrahydrofuran (THF, 0.5 mL) to form a homogeneous solution. These solutions were rapidly injected into deionized (D.I.) water (9 mL) under continuous sonication. After addition, the mixture was kept sonicated for 10 min in an ice-water bath. Then, THF was removed with a rotary evaporator. The resulting deep color aqueous solution was washed with D.I. water three times, using a 10 K centrifugal filter (Millipore) under centrifugation at 2040 × *g* for 15 min. The amount of **F1**^2+^(BF_4_^−^)_2_ and MEH-PPV (NIR775) were determined by indirectly measuring the UV–visible absorption spectra of filtrate, showing that nearly all **F1**^2+^(BF_4_^−^)_2_, MEH-PPV, and NIR775 were encapsulated into nanoparticles.

To prepare **F1**^2+^-ANP-NH_2_, **F1**^2+^(BF_4_^−^)_2_ (0.52 mg), MEH-PPV (0.25 mg), NIR775 (0.02 mg), DSPE-PEG_2000_ (8 mg), and DSPE-PEG_2000_-NH_2_ (2 mg) were dissolved in THF (0.5 mL) to form a homogeneous solution. These solutions were rapidly injected into D.I. water (9 mL) under continuous sonication. After addition, the mixture was kept sonicated for 10 min in an ice-water bath. Then, THF was removed with a rotary evaporator. The resulting deep color aqueous solution was washed with D.I. water three times, using a 10 K centrifugal filter (Millipore) under centrifugation at 2040 × *g* for 15 min. The **F1**^2+^-ANP-NH_2_ solution was finally concentrated to 252 μg mL^−1^ (based on the mass of MEH-PPV) by ultrafiltration, and used for the preparation of **F1**^2+^-ANP-Gal. Typically, β-Gal-COOH (1.5 mg), 1-(3-dimethylaminopropyl)-3-ethylcarbodiimide hydrochloride (3.0 mg), and *N*-hydroxy succinimide (2 mg) were dissolved in 1.0 mL PBS (1×, pH 7.4) and the solution was stirred at room temperature (r.t.) for 1 h. Then, **F1**^2+^-ANP-NH_2_ (522/252/19.8 μg mL^−1^ **F1**^2+^(BF_4_^−^)_2_/MEH-PPV/NIR775) was added, and the mixture was kept stirring at r.t. for another 6 h. The reaction solution was then washed with PBS for three times via using centrifugal filter units with molecular weight cutoff of 10 kDa under centrifugation at 2040 × *g* to remove excess reactant. The stock solution was stored at 4 °C under dark. The amount of **F1**^2+^(BF_4_^−^)_2_ and MEH-PPV (NIR775) were determined by indirectly measuring the UV–visible–NIR absorption of filtrate, showing that nearly all **F1**^2+^(BF_4_^−^)_2_, MEH-PPV and NIR775 were encapsulated into nanoparticles.

### Measurement of the fluorescence and afterglow luminescence

To measure the fluorescence of **F1**^2+^-ANP or **F1**^2+^-ANP-Gal in response to H_2_S, **F1**^2+^-ANP, or **F1**^2+^-ANP-Gal (2.2 μg NIR775, 28 μg MEH-PPV, 58 μg F**1**^2+^(BF_4_^−^)_2_) in 1.0 mL PBS buffer (pH 7.4) were incubated with NaHS (200 µM) at 37 °C for 1 min, and the fluorescence spectra were recorded on a HORIBA Jobin Yvon Fluoromax-4 fluorometer with fluorescence synchronous scanning mode (*λ*_ex_ = 400–800 nm, offset = 100 nm).

To measure the afterglow luminescence of **F1**^2+^-ANP or **F1**^2+^-ANP-Gal in response to H_2_S, **F1**^2+^-ANP, or **F1**^2+^-ANP-Gal (2.2 μg NIR775, 28 μg MEH-PPV, 58 μg **F1**^2+^(BF_4_^−^)_2_) in 1.0 mL PBS buffer (pH 7.4) were incubated with 200 μM NaHS at 37 °C for 1 min. Then, the incubation solutions were illuminated by an 808-nm laser (1 W cm^−2^) for 1 min. The laser was removed, and the afterglow luminescence images were acquired on an IVIS Spectrum imaging system equipped with an open filter or a 790 nm emission filter, with an acquisition time of 60 s. The afterglow luminescence intensity in each image was quantified by applying region of interest (ROI) over the image, using the Living Image Software (4.5.2, PerkinElmer, MA, USA).

### Cell culture

Human HCC HepG2 cells and human lung cancer A549 cancer cells were purchased from Stem Cell Bank, Chinese Academy of Sciences (Shanghai, China). All cell lines were routinely tested to exclude infection with mycoplasma. They were authenticated by the supplier using short tandem repeat test. Human HCC HepG2 cells were cultured in Dulbecco’s modified Eagle’s medium (DMEM). Human lung cancer A549 cancer cells were grown in F-12 medium. All the mediums were supplemented with 10% fetal bovine serum (FBS), 1% penicillin, and streptomycin. All cells were maintained at 37 °C in a humidified atmosphere with 5% CO_2_.

### Imaging of H_2_S in HepG2 cells

HepG2 Cells were plated at a density of 5 × 10^4^ cells per well onto a glass-bottom dish (In Vitro Scientific, D35-20-1-N), and grown for 12 h. Cells were then exposed to various concentrations of **F1**^2+^-ANP-Gal (2, 7, 14, 28, 38, and 48 μg mL^−1^ based on mass concentration of MEH-PPV) at 37 °C for different times (0.5, 1, 2, 3, 4, and 6 h). To image the elevated H_2_S levels in living cells, HepG2 cells were incubated with **F1**^2+^-ANP-Gal (2.2/28/58 μg mL^−1^ NIR775/MEH-PPV/**F1**^2+^(BF_4_^−^)_2_) for 3 h, and then the medium was discarded and washed with cold PBS (1×, pH 7.4) once, followed by incubation at 37 °C for 1 h in the DMEM medium containing 1 mM NaHS. To elevate the endogenous H_2_S production, HepG2 cells were incubated with 0.2 mM l-Cys for 1 h; to image the down-regulated H_2_S levels in living cells, HepG2 cells were pre-treated with ZnCl_2_ (0.3 mM) for 10 min to scavenge the endogenous H_2_S; alternately, PAG (50 mg L^−1^) and AOAA (20 μM) were added into the cells, and then incubated for 0.5 h to inhibit the endogenous CSE and CBS activities, followed by adding l-Cys (0.2 mM) and incubating for another 1 h. Then, cells were incubated with **F1**^2+^-ANP-Gal (2.2/28/58 μg mL^−1^ NIR775/MEH-PPV/**F1**^2+^(BF_4_^−^)_2_) for 3 h. Cells were then stained with 2.0 µM Hoechst 33342 for another 20 min. Prior to fluorescence imaging, cells were washed with cold PBS (1×, pH 7.4) three times. After adding fresh medium, the fluorescence images were acquired on an Olympus IX73 fluorescent inverted microscope equipped with DAPI (4′,6-diamidino-2-phenylindole) and TRITC (tetramethylrhodamine) filter.

For afterglow imaging of cells, HepG2 cells were plated at a density of 3 × 10^4^ (or 1 × 10^5^) cells into culture dish. Cells were treated according to the above-mentioned procedures. Cells were harvested from the culture dish by digestion with trypsin. The cell pellets were then collected after centrifugation at 161 × *g* for 4 min. The cell pellets were irradiated with the 808-nm laser (1 W cm^−2^) for 1 min. After removal of the laser, the afterglow luminescence images were immediately acquired on an IVIS Spectrum imaging system equipped with an open filter, with an acquisition time of 60 s. The afterglow luminescence intensities in the cell pellets were quantified by applying ROI over the images, using the Living Image Software (4.5.2, PerkinElmer, MA, USA).

### Investigation of tissue-penetration ability

To investigate the tissue-penetration ability, **F1**^2+^-ANP-Gal (0.026/0.33/0.684 mg mL^−1^ NIR775/MEH-PPV/**F1**^2+^(BF_4_^−^)_2_, 200 µL) in PBS buffer (pH 7.4) was incubated with NaHS (2.0 mM) at 37 °C for 10 min. The incubation solution was then irradiated with the 808-nm laser (1 W cm^−2^) for 1 min. After removal of laser, the solution was covered with chicken tissues at varying thickness (0, 1.5, 2.0, 3.0 and 4.0 cm). The afterglow images were acquired on an IVIS Spectrum imaging system equipped with an open filter, with an acquisition time of 60 s. To investigate the tissue-penetration ability through a mouse body, a solution of H_2_S-activated **F1**^2+^-ANP-Gal (0.026/0.33/0.684 mg mL^−1^ NIR775/MEH-PPV/**F1**^2+^(BF_4_^−^)_2_, 50 µL) was irradiated with the 808-nm laser (1 W cm^−2^, 1 min), and then placed under a living mouse. The afterglow luminescence images were acquired on an IVIS Spectrum imaging system equipped with an open filter, with an acquisition time of 60 s. The fluorescence images were acquired on an IVIS Lumina XR III imaging system with λ_ex_/λ_em_ = 740/790 nm. Each experiment was conducted for three times.

### Animals and tumor models

BALB/c mice (~5–6 weeks old) were purchased from the Model Animal Research Center (MARC) of Nanjing University in China. All animal experiments were performed in compliance with the Guidelines established by the Institutional Animal Care and Use Committee (IACUC) of Nanjing University.

To establish tumors, HepG2 cells (2.0 × 10^6^ cells, 50 μL) suspended in 50 v/v% mixture of Matrigel in supplemented DMEM (10% FBS, 1% pen/strep (100 U mL^−1^ penicillin and 100 µg mL^−1^ streptomycin)) were injected subcutaneously into the selected positions of nude mice. The tumor volume (*V*) was determined assuming ellipsoid shape with the formula of *V* = (*L* × *W*^2^)/2, where the length (*L*) and width (*W*) of each tumor was measured using a caliper. When the tumor volume reached about 12–100 mm^3^, fluorescence and afterglow imaging were conducted. To establish an orthotopic HCC model, a midline incision of the anterior abdominal wall was made. HepG2 cells transfected with luciferase (HepG2/Luc) (2 × 10^6^, 100 μL) in serum-free culture medium were carefully injected into the lube of liver of mice under anesthesia by pentobarbital sodium. Tumor growths were monitored by BL imaging. After 2 weeks, mice with HCC model were successfully established for afterglow and fluorescence imaging.

### Imaging of tumor H_2_S in vivo

To image H_2_S in HepG2 tumors, mice bearing s.c. HepG2 tumors were i.v. injected with **F1**^2+^-ANP-Gal or **F1**^2+^-ANP (211/100/8 μg **F1**^2+^(BF_4_^−^)_2_/MEH-PPV/NIR775, in 200 μL saline). After 3.5 h, l-Cys (1 mM, 25 µL) or ZnCl_2_ (1 mM, 25 µL) was directly injected into tumors to regulate tumor H_2_S levels. The fluorescence and afterglow images were acquired at 0, 4, 8, 12, and 24 h using an IVIS Lumina XR III imaging system. To image H_2_S in different sizes of HepG2 tumors, HepG2 tumors at an average size of ~12, ~45, and ~100 mm^3^ were i.v. injected with **F1**^2+^-ANP-Gal (211/100/8 μg **F1**^2+^(BF_4_^−^)_2_/MEH-PPV/NIR775, in 200 μL saline). The fluorescence and afterglow images were acquired at 12 h post injection. To image H_2_S in orthotopic HepG2/Luc tumors, mice with orthotopic HepG2/Luc tumors and control mice were i.v. injected with **F1**^2+^-ANP-Gal (211/100/8 μg **F1**^2+^(BF_4_^−^)_2_/MEH-PPV/NIR775, in 200 μL saline). The fluorescence and afterglow images were acquired at 12 h post injection.

The fluorescence images were acquired on an IVIS Lumina XR III imaging system, with λ_ex_/λ_em_ = 740/790 nm. To acquire afterglow images, the mouse body at 0, 4, 8, 12, and 24 h post i.v. injection of **F1**^2+^-ANP-Gal was irradiated under the 808-nm laser (1 W cm^−2^) for 1 min. After cessation of laser, the afterglow luminescence images were immediately acquired on an IVIS Spectrum imaging system equipped with an open filter, with an acquisition time of 60 s. Every experiment was conducted in three mice. ROIs were drawn over the tumor and the thigh of the mouse to quantify the fluorescence and afterglow intensities using the Living Image Software. The SBRs were calculated by dividing the fluorescence and afterglow intensities in tumor to that in thigh.

### WB analysis of CSE and CBS expression

To conduct the WB analysis of mouse liver and orthotopic HepG2 tumor tissue homogenates, three normal mice and mice with orthotopic liver tumors were euthanized, and the livers from normal mice and the HepG2 tumor tissues from the orthotopic liver tumor-bearing mice were resected. Liver and HepG2 tumor tissues were then homogenized with lysis buffer. The homogenates were incubated on ice for 30 min, and the lysates were obtained by centrifugation (13,000 × *g*, 15 min) at 4 °C. Aliquots of proteins were separated by sodium dodecyl sulfate-polyacrylamide gel electrophoresis (10%) and subsequently transferred to nitrocellulose membranes by electroblotting. The membrane was blocked in 5% skim milk powder in 0.1% Tris-buffered saline/Tween-20 (TBST, 20 mM Tris-HCl, pH 7.4, 137 mM NaCl, and 0.1% Tween) at r.t. for 2 h, and then incubated with antibody raised against CBS (Abcam, ab135626, lot: GR3265732-10, dilution 1 : 1000) for 2 h at r.t. After three washes with TBST, the membrane was incubated with a secondary antibody horseradish peroxidase (HRP)-conjugated goat anti-rabbit immunoglobulin G (IgG) for 2 h at r.t. The films were developed with the ECL system. The CBS protein and markers were visualized using G:BOX chemiXR5. Afterwards, the membrane was rinsed in D.I. water at r.t. for 5 min, and antibody stripping buffer (weak alkaline) was added. The membrane was then rinsed with D.I. water at r.t. for 5 min, blocked in 5% skim milk powder in 0.1% Tris-buffered saline/Tween-20 at r.t. for 2 h, and incubated with antibody raised against CSE (Abcam, ab151769, lot: GR3257101-3, dilution 1 : 1000) for 2 h at r.t. After three washes with TBST, the membrane was incubated with a secondary antibody HRP-conjugated goat anti-rabbit IgG at r.t. for 2 h. The films were developed with the ECL system. The CSE protein and markers were visualized using G:BOX chemiXR5. The resulting band intensities were quantified using a Gel-Pro32. Each experiment was repeated for three times.

### Measurement of the H2S concentration in human blood

Whole-blood samples were collected from healthy volunteers and the patients with HCC and CRC at Affiliated Drum Tower Hospital of Nanjing University (Nanjing, China). For the detection of H_2_S in blood samples of healthy persons and patients diagnosing with HCC or CRC, freshly collected bloods were 2-fold diluted with PBS buffer (1×, pH 7.4), and then incubated with **F1**^2+^-ANP (2.2/28/58 μg mL^−1^ NIR775/MEH-PPV/**F1**^2+^(BF_4_^−^)_2_) at 37 °C for 1 min, followed by irradiation with the 808-nm laser (1 W cm^−2^) for 1 min. After removal of laser, the afterglow luminescence images were immediately acquired for 60 s with a 790 nm filter. The afterglow luminescence intensities at 790 nm for **F1**^2+^-ANP were quantified by applying ROI over the image using the Living Image Software (4.5.2, PerkinElmer, MA, USA). With constructing a standard curve in healthy blood by an internal standard method, the concentrations of H_2_S in whole bloods of healthy persons and HCC or CRC patients were obtained.

### Imaging of human HCC specimens

HCC specimens were collected from the patients with HCC at Affiliated Drum Tower Hospital of Nanjing University (Nanjing, China). This study was approved by institutional review board of Affiliated Drum Tower Hospital of Nanjing University, and all subjects provided written informed consent under institutional review board prior to sample collection. HCC specimens were resected from four HCC patients. HCC specimens were infiltrated with **F1**^2+^-ANP-Gal (58/28/2.2 μg mL^−1^ **F1**^2+^(BF_4_^−^)_2_/MEH-PPV/NIR775) in PBS buffer for 3 h. The specimens were then rinsed with PBS buffer (1×, pH 7.4) three times to remove nonspecifically absorbed **F1**^2+^-ANP-Gal on the tissues. The specimens were irradiated with the 808-nm laser (1 W cm^−2^, 1 min). After removal of laser, the afterglow images were acquired on an IVIS Spectrum imaging system equipped with an open filter, with an acquisition time of 60 s. The fluorescence images were acquired with *λ*_ex/em_ = 740/790 nm. ROIs were drawn over the tumor and adjacent normal liver tissues to quantify the fluorescence and afterglow intensities using the Living Image Software. The SBRs were calculated by dividing the fluorescence and afterglow intensities in tumor to that in normal liver tissues.

### Immunohistochemistry studies

HCC tissues were fixed in 4% formalin and then embedded in paraffin before 10-µm sectioning. Histology samples were stained by H&E using a standard protocol. White light images were acquired using an IX73 optical microscope equipped with a color camera.

### Statistical analysis

Results are expressed as the mean ± standard deviation unless otherwise stated. Statistical differences among experimental groups were analyzed by Student’s *t* test. *P* < 0.05 was considered statistically significant. All statistical calculations were performed using GraphPad Prism 6 including assumptions of tests used (GraphPad Software Inc., CA, USA).

### Reporting summary

Further information on research design is available in the Nature Research Reporting Summary linked to this article.

## Supplementary information

Supplementary Information

Description of Additional Supplementary Files

Supplementary Data 1

Reporting Summary

## Data Availability

The source data underlying Figs. 2b, e, f, h, i, 3d, g, 4c, e, f, 5c, d, f, g, 6b, c and 7e and Supplementary Figs. 2d–i, 5c, f, 8, 9d, 11, 12b, 14c, d, 15, 16d, 17a–c, j–l, 18, 19b, d, 20b, d, 22, 21a, c, 23a, b, f, 24, 28b, d, 29b, c, 30c, 31b, e, 32, 33, 35b, 37, 38b, 39d, 40d, 41d, 42c, d, 43b and 44 are provided as a Source Data file. The authors declare that all other data related to this study are available in the article/and or its Supplementary Information files or from the authors upon reasonable request.

## Source data

Source Data
