# Supplementary material for: H2S-activatable near-infrared afterglow luminescent probes for sensitive molecular imaging in vivo
Source: Nat Commun. 2020 Jan 23;11:446. doi: 10.1038/s41467-020-14307-y (PMC6978336; doi:10.1038/s41467-020-14307-y)
Supplement: Supplementary file 1 — Supplementary Information [file 41467_2020_14307_MOESM1_ESM.pdf]

Supplementary Information

H_2S-Activatable Near-infrared Afterglow Luminescent Probes for Sensitive Molecular Imaging in vivo

Wu et al.

Table of Contents

		Page
1	Supplementary Figures 1-53	3-43
2	Supplementary Methods	44-52
3	Supplementary Notes 1-2	52-53

Supplementary Figures

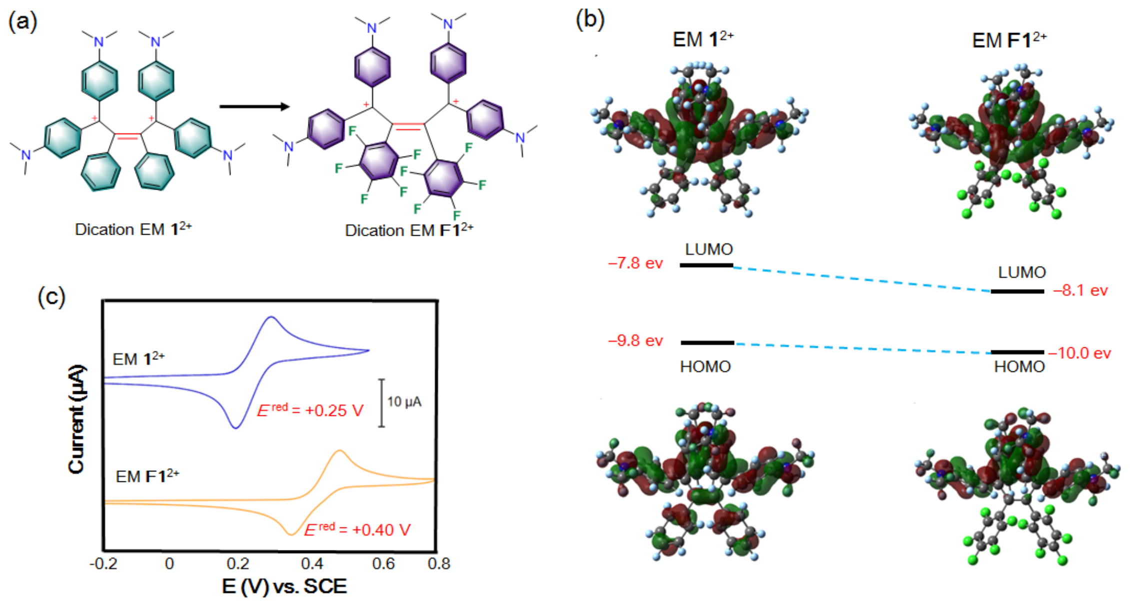

Supplementary Figure 1. Optimization of EM 1^{2+} into EM $\mathbf{F1}^{2+}$. (**a**) Chemical structures of dication EM $\mathbf{1}^{2+}$and $\mathbf{F1}^{2+}$. (**b**) Highest occupied molecular orbital (HOMO) and lowest unoccupied molecular orbital (LUMO) of $\mathbf{1}^{2+}$ and $\mathbf{F1}^{2+}$ based on density functional theory (DFT) calculations (B3LYP/6-31G*). (**c**) Cyclic voltammetry (CV) of $\mathbf{1}^{2+}$ (blue) and $\mathbf{F1}^{2+}$ (orange) in CH_2Cl_2 containing 0.1 M Bu_4NBF_4 as a supporting electrolyte (scan rate 100 mV s^{-1}, Pt electrode). $\mathbf{F1}^{2+}$ with two electron-withdrawing pentafluorophenyl groups hold more positive E^{red} (+0.40 V vs. SCE) compared to $\mathbf{1}^{2+}$ (+0.25 V vs. SCE), indicating a higher ability of $\mathbf{F1}^{2+}$ to accept electrons from H_2S. E^{red}: halfwave potentials for reductions; SCE: saturated calomel electrode.

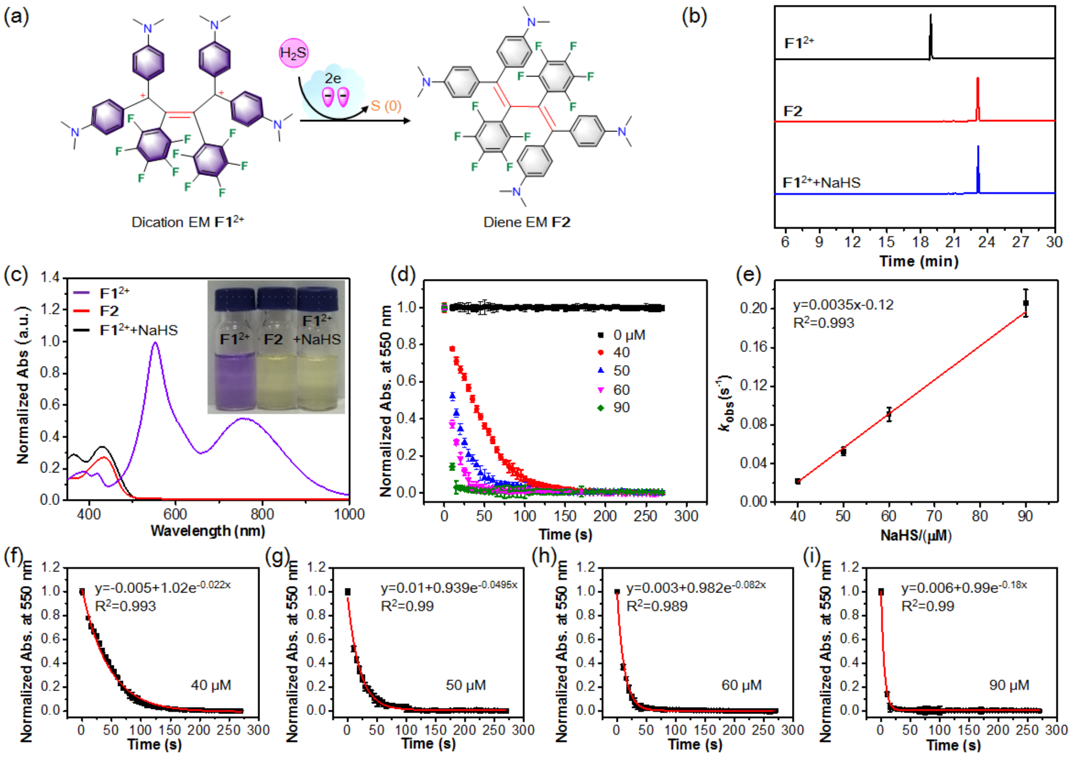

Supplementary Figure 2. Characterization of the reaction between **F1^{2+}** and NaHS in vitro. (**a**) Proposed conversion of EM **F1^{2+}** to diene **F2** upon reduction by H_2S. (**b**) HPLC traces and (**c**) absorption spectra of **F1^{2+}**, **F2**, and **F1^{2+}** (9 μg mL^{-1}) following incubation with 90 μM NaHS in PBS buffer (pH 7.4， 5% DMSO) at 37 ºC for 1 min. Inset: photographs of **F1^{2+}**, **F2** and **F1^{2+}** following treatment with NaHS in PBS buffer (1×, pH 7.4). (**d**) Normalized time-dependent decline of UV-vis absorption (550 nm) of **F1^{2+}**$(BF_4^-)_2$ (9 μg mL^{-1}) following incubation with different concentration of NaHS in PBS buffer (pH 7.4) at r.t.. (**e**) Plot of the pseudo-first-order rate (k_{obs}) versus NaHS concentration (40-90 μM) afford the second-order reaction rate k_2 = 3600 ± 130 M^{-1} s^{-1} between **F1^{2+}** and NaHS in PBS buffer at r.t.. The k_{obs} was determined by fitting the absorption intensity with single exponential function of $y = y_0 + A \times \exp(R_0 \times t)$, where $k_{obs} = -R_0$, and the k_2 value was obtained from the slope of the linear plot between k_{obs} and NaHS concentration (R^2 = 0.998). (**f-i**) The exponential fit curves of **F1^{2+}**$(BF_4^-)_2$ (9 μg mL^{-1}) following incubation with 40 (**f**), 50 (**g**), 60 (**h**), and 90 μM (**i**) NaHS in PBS buffer (pH 7.4) at r.t. Data denote mean ± standard deviation (s.d.) (n = 3). Source data are provided as a Source Data file.

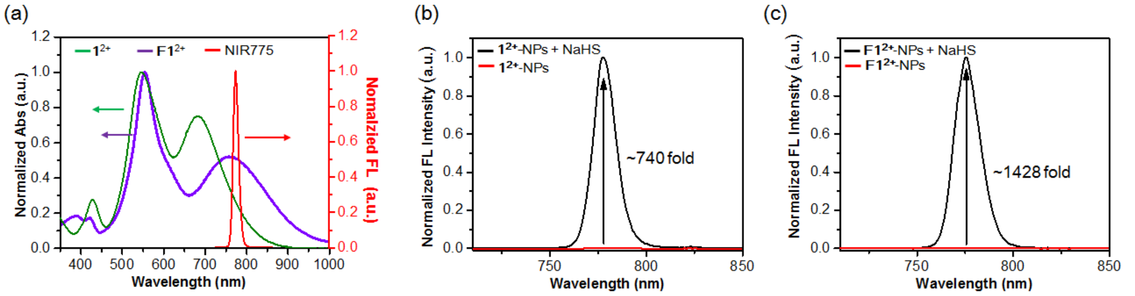

Supplementary Figure 3. Characterization of **F1**$^{2+}$-NPs and **1**$^{2+}$-NPs in vitro. (**a**) Normalized UV−vis-NIR absorption of EM **1**$^{2+}$ (green), **F1**$^{2+}$ (violet) and fluorescence emission of NIR775 (red). A better overlap between **F1**$^{2+}$ absorption and NIR775 emission was observed compared to that between **1**$^{2+}$ absorption and NIR775 emission. (**b**) Fluorescence spectra of micellar nanoparticles (**1**$^{2+}$-NIR775) containing **1**$^{2+}$(BF_4^-)$_2$ (48 μg mL^{-1} = 56 μM) and NIR775 (2.2 μg mL^{-1}) upon incubation with NaHS (350 μM) at r.t. for 10 min. (**c**) Fluorescence spectra of micellar nanoparticles (**F1**$^{2+}$-NIR775) containing **F1**$^{2+}$(BF_4^-)$_2$ (58 μg mL^{-1} = 56 μM) and NIR775 (2.2 μg mL^{-1}) upon incubation with 200 μM NaHS at r.t. for 1 min. A higher turn-on ratio was observed for **F1**$^{2+}$-NIR775 compare to **1**$^{2+}$-NIR775, suggesting that **F1**$^{2+}$ can quench the NIR775 fluorescence more efficiently than **1**$^{2+}$.

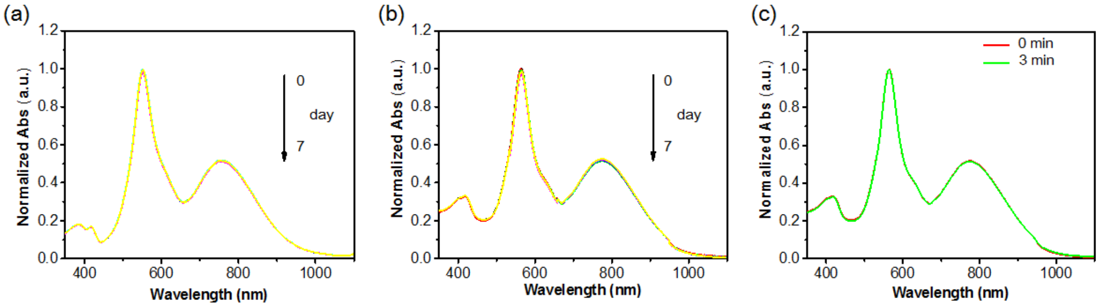

Supplementary Figure 4. Evaluation of the stability of **F1**$^{2+}$ and **F1**$^{2+}$-NPs in vitro. (**a**) Normalized absorption spectra of **F1**$^{2+}$(BF_4^-)$_2$ (9 μg mL^{-1}) following incubation in PBS buffer (pH 7.4) at r.t. for 7 days. (**b**) Normalized absorption spectra of **F1**$^{2+}$(BF_4^-)$_2$ containing micellar nanoparticles (**F1**$^{2+}$-NPs, 9 μg mL^{-1} **F1**$^{2+}$(BF_4^-)$_2$) following incubation in PBS buffer (pH 7.4) at r.t. for 7 days. (**c**) Normalized absorption spectra of **F1**$^{2+}$-NPs (9 μg mL^{-1} **F1**$^{2+}$(BF_4^-)$_2$) before and after irradiation with an 808 nm laser (1 W cm^{-2}) for 3 min. The results demonstrated that **F1**$^{2+}$ was stable and resistant to photobleaching.

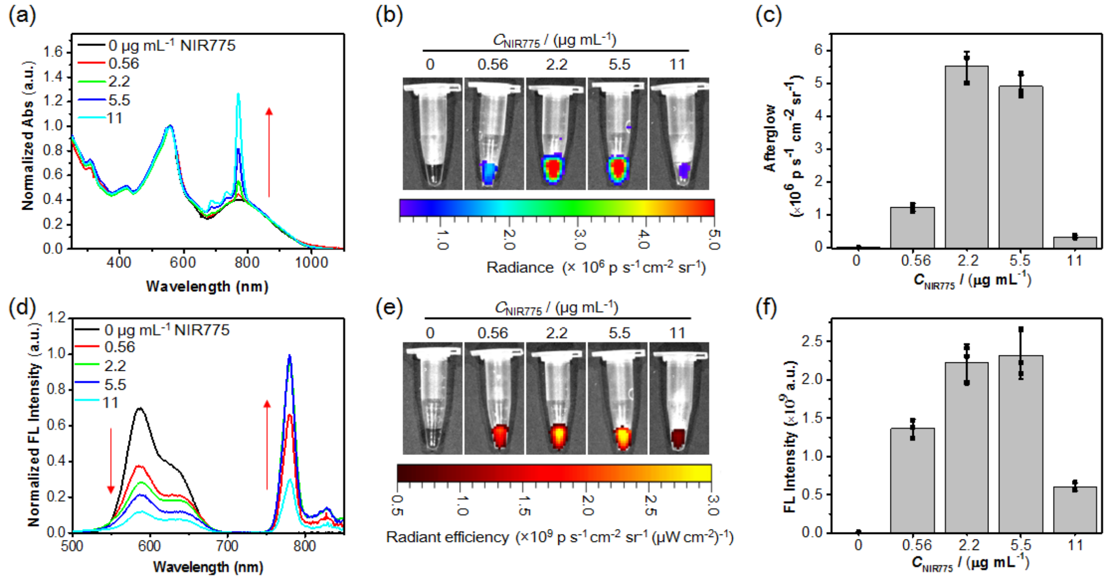

Supplementary Figure 5. Optimization of the loading amount of NIR775 in **F1**$^{2+}$-ANP. (**a**) Normalized UV-vis-NIR absorption of **F1**$^{2+}$-ANP (58/28 μg mL^{-1} **F1**$^{2+}$(BF_4^-)$_2$/MEH-PPV) loading with 0, 0.56, 2.2, 5.5, and 11 μg mL^{-1} NIR775. (**b**) Afterglow luminescence images and (**c**) intensities of **F1**$^{2+}$-ANP (58/28 μg mL^{-1} **F1**$^{2+}$(BF_4^-)$_2$/MEH-PPV) loading with indicated amount of NIR775 upon treatment with NaHS (200 μM, 1 min), followed by illumination with 808 nm laser (1 W cm^{-2}) at 37 ºC for 1 min. After removal of the laser, the afterglow luminescence images were immediately acquired under an open filter, with an acquisition time of 60 s. (**d**) Fluorescence spectra, (**e**) images and (**f**) intensities of **F1**$^{2+}$-ANP loading with indicated amount of NIR775 upon treatment with NaHS (200 μM, 1 min) in PBS buffer (pH 7.4). Values are mean ± s.d. (n = 3). It was shown that the fluorescence of MEH-PPV at 580 nm decreased with the loading amount of NIR775, while the maximum fluorescence intensity of NIR775 at 780 nm was observed when the loading amount of NIR775 was at 2.2 and 5.5 μg mL^{-1}, matching well that of afterglow images observed in (**b**). These results suggested that the optimum loading ratio among MEH-PPV, NIR 775 and **F1**$^{2+}$(BF_4^-)$_2$ within **F1**$^{2+}$-ANP was ~28/2.2/58 (by mass). Source data are provided as a Source Data file.

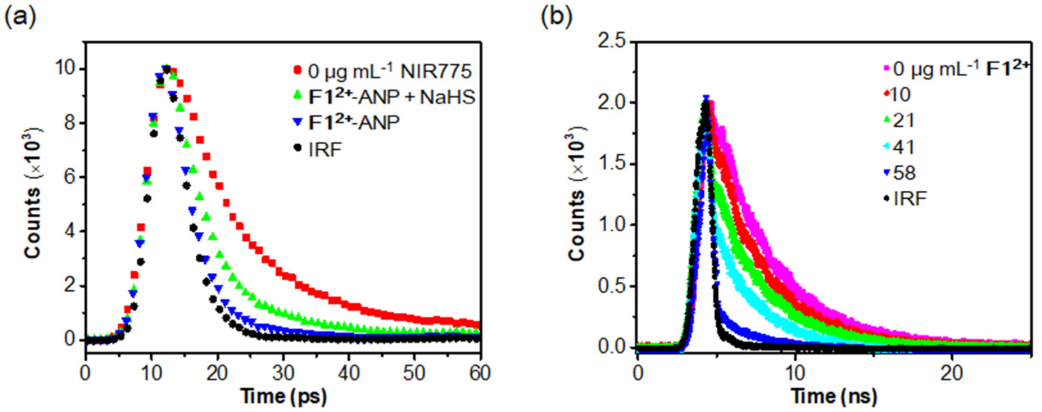

Supplementary Figure 6. Fluorescence lifetime measurement of MEH-PPV or NIR775. (**a**) Fluorescence decay curves of MEH-PPV within **F1**$^{2+}$-ANP, H_2S-activated **F1**$^{2+}$-ANP, and H_2S-activated nanoparticles containing **F1**$^{2+}$(BF_4^-)$_2$/MEH-PPV (58/28 μg mL^{-1}) but without NIR775 (0 μg mL^{-1} NIR775). (**b**) Fluorescence decay curves of NIR775 within **F1**$^{2+}$-ANP (58 μg mL^{-1} **F1**$^{2+}$), H_2S-activated **F1**$^{2+}$-ANP (0 μg mL^{-1} **F1**$^{2+}$), and nanoparticles containing MEH-PPV/NIR775 (28/2.2 μg mL^{-1}) along with different amount of EM **F1**$^{2+}$ (10, 21, and 41 μg mL^{-1}). Instrument response function (IRF) (black) is also indicated.

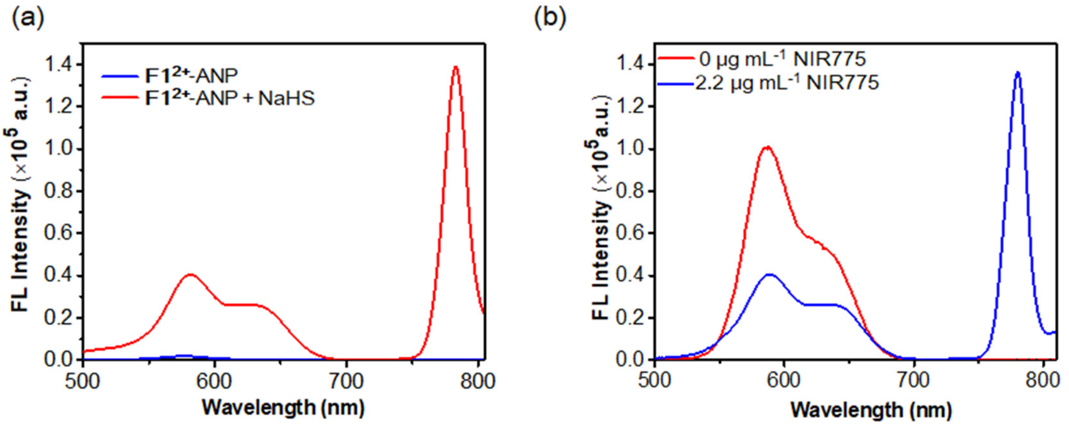

Supplementary Figure 7. Evaluation of the energy transfer efficiency from donor to acceptor. (**a**) Fluorescence spectra of **F1**$^{2+}$-ANP (58/28/2.2 μg mL^{-1} **F1**$^{2+}$(BF_4^-)$_2$/MEH-PPV/NIR775) upon incubation with or without NaHS (200 μM) in PBS (1×, pH 7.4) at 37 ºC for 1 min. (**b**) Fluorescence spectra of **F1**$^{2+}$-ANP (58/28/2.2 μg mL^{-1} **F1**$^{2+}$(BF_4^-)$_2$/MEH-PPV/NIR775) (blue) and nanoparticles containing **F1**$^{2+}$(BF_4^-)$_2$/MEH-PPV (58/28 μg mL^{-1}) but without NIR775 (0 μg mL^{-1}) (red) following activation with NaHS (200 μM, 1 min) in PBS buffer (1×, pH 7.4).

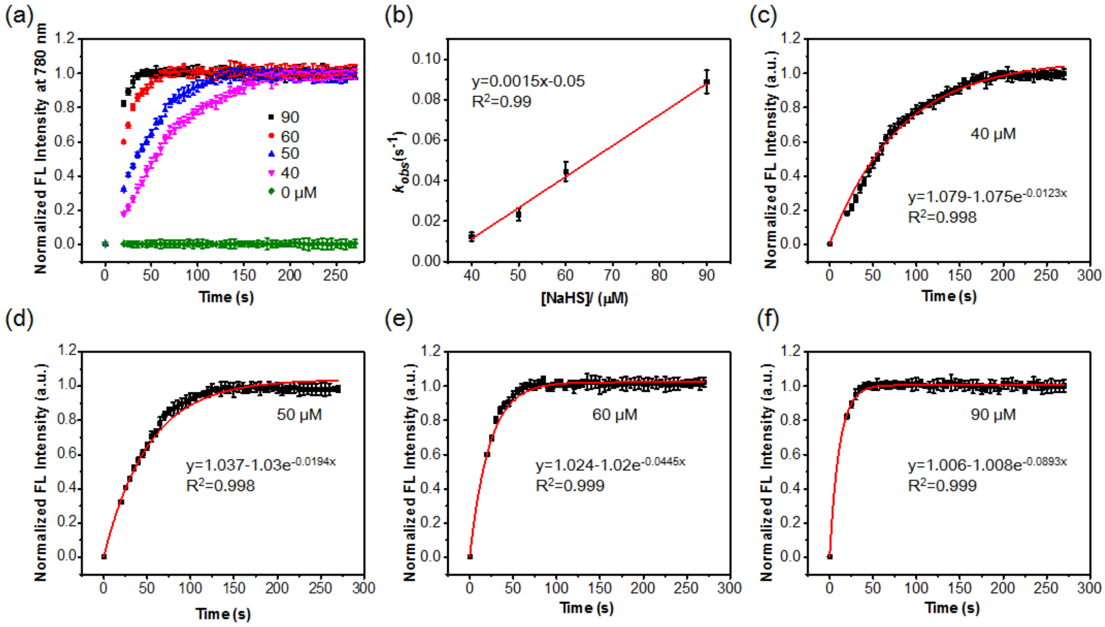

Supplementary Figure 8. Measurement of reaction kinetics of **F1**$^{2+}$-ANP toward NaHS. (**a**) Normalized time-dependent fluorescence intensities ($\lambda_{em/em}$ = 740/790 nm) of **F1**$^{2+}$-ANP (9/4.5/0.35 μg mL^{-1} **F1**$^{2+}$(BF_4^-)$_2$/MEH-PPV/NIR775) following incubation with varying concentrations of NaHS (0, 40, 50, 60 and 90 μM) in PBS buffer (pH 7.4) at r.t.. (**b**) Plot of the pseudo-first-order rate (k_{obs}) versus NaHS concentration (40-90 μM) afford the second-order reaction rate between **F1**$^{2+}$-ANP and NaHS at r.t.. The k_{obs} was determined by fitting the FL intensity with single exponential function of $y = y_0 + A' \times \exp(R_0 \times t)$, where $k_{obs} = -R_0$, and the k_2 value was obtained from the slope of the linear plot between k_{obs} and NaHS concentration (R^2 = 0.993). The apparent k_2 for **F1**$^{2+}$-ANP was found to be $k_2 = 1563 \pm 141$ M^{-1} s^{-1}, which was ~2-fold lower than that of the homogeneously dispersed **F1**$^{2+}$. (**c-f**) The exponential fit curves of **F1**$^{2+}$-ANP following incubation with 40 (**c**), 50 (**d**), 60 (**e**), and 90 μM (**f**) NaHS in PBS buffer (pH 7.4) at r.t.. Values are mean ± s.d. (n = 3). We postulated that the slower kinetics between **F1**$^{2+}$-ANP and NaHS than that between free EM **F1**$^{2+}$ and NaHS could be due to: 1) because EM **F1**$^{2+}$ molecules are encapsulated within the interior of **F1**$^{2+}$-ANP, it may have to take some time for the H_2S molecules to across the phospholipid layer on **F1**$^{2+}$-ANP and diffuse into the nanoparticles; 2) as the environment within **F1**$^{2+}$-ANP was stickier and more hydrophobic compared to the exterior aqueous buffer, EM **F1**$^{2+}$ molecules may be not homogeneously dispersed, but packed closely as aggregates within **F1**$^{2+}$-ANP. This will increase the steric hindrance for H_2S molecules to reduce EM **F1**$^{2+}$ presenting in the interior of the aggregates, which will also prolong the reaction. Source data are provided as a Source Data file.

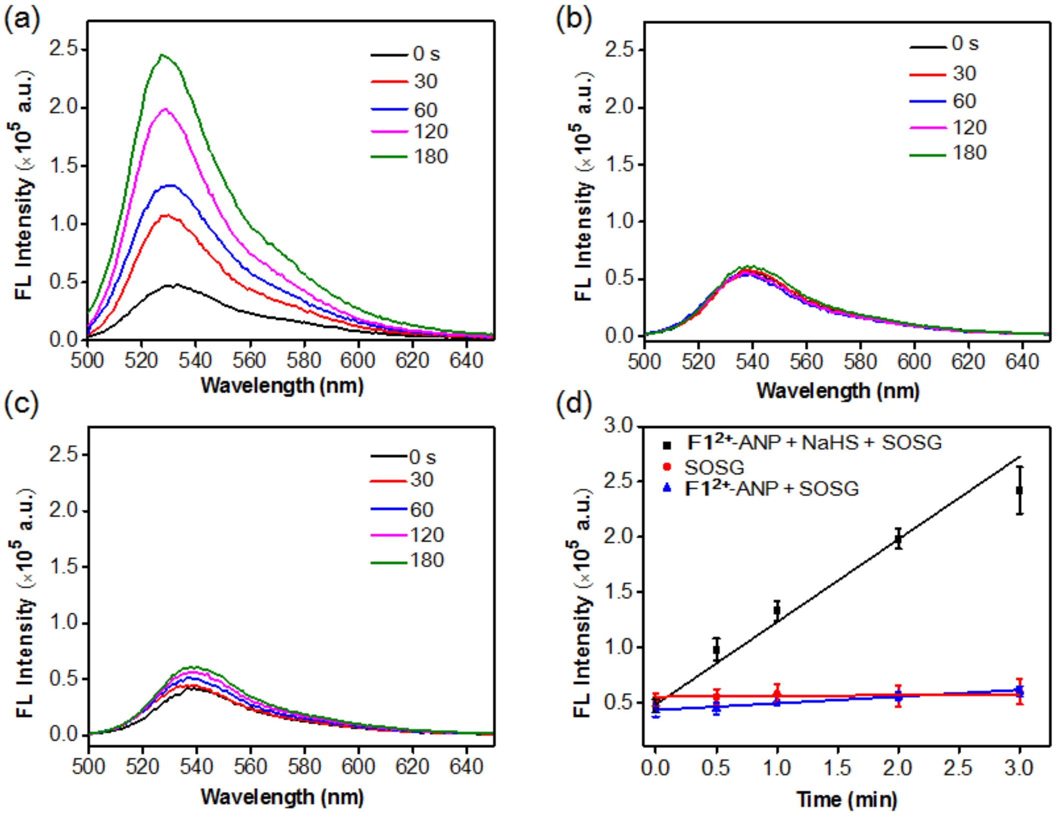

Supplementary Figure 9. Comparison of $^1O_2$ generation ability of **F1**$^{2+}$-ANP using SOSG. (**a**) Fluorescence spectra of SOSG (20.0 μM) in the presence of **F1**$^{2+}$-ANP (58/28/2.2 μg mL^{-1} **F1**$^{2+}$(BF_4^-)$_2$/MEH-PPV/NIR775) and NaHS (200 μM), (**b**) SOSG alone, and (**c**) SOSG (20.0 μM) in the presence of **F1**$^{2+}$-ANP in PBS buffer (1×, pH 7.4), following irradiation with an 808 nm laser (1 W cm^{-2}) for 0, 30, 60, 120 and 180 s. (**d**) Plots of the SOSG fluorescence intensities at 528 nm in indicated solutions as a function of irradiation time. Values are mean ± s.d. (n = 3). The fluorescence spectra were recorded with $\lambda_{ex}/\lambda_{em}$ = 488/528 nm. Source data are provided as a Source Data file. The results showed that **F1**$^{2+}$-ANP had little $^1O_2$ generation capacity upon 808 nm laser irradiation, while **F1**$^{2+}$-ANP after activation with H_2S showed obvious $^1O_2$ generation, suggesting that H_2S could efficiently switch on the $^1O_2$ generation ability of **F1**$^{2+}$-ANP. Source data are provided as a Source Data file.

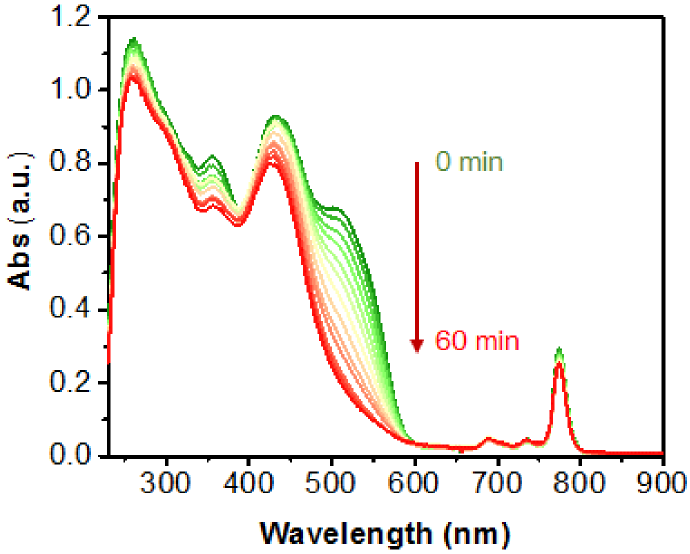

Supplementary Figure 10. Monitoring of the oxidation and degradation of MEH-PPV. UV-vis-NIR absorption spectra of **F1**$^{2+}$-ANP (29/14/1.1 μg mL^{-1} **F1**$^{2+}$(BF_4^-)$_2$/MEH-PPV/NIR775) in the presence of 200 μM NaHS, followed by illumination with the 808 nm laser (1 W cm^{-2}) for different time (0, 5, 10, 15, 20, 25, 30, 35, 40, 45, 50, 55, 60 min). The gradual decline in the absorption of MEH-PPV indicates that the in situ formation of $^1O_2$ upon laser irradiation can trigger the oxidation and degradation of MEH-PPV.

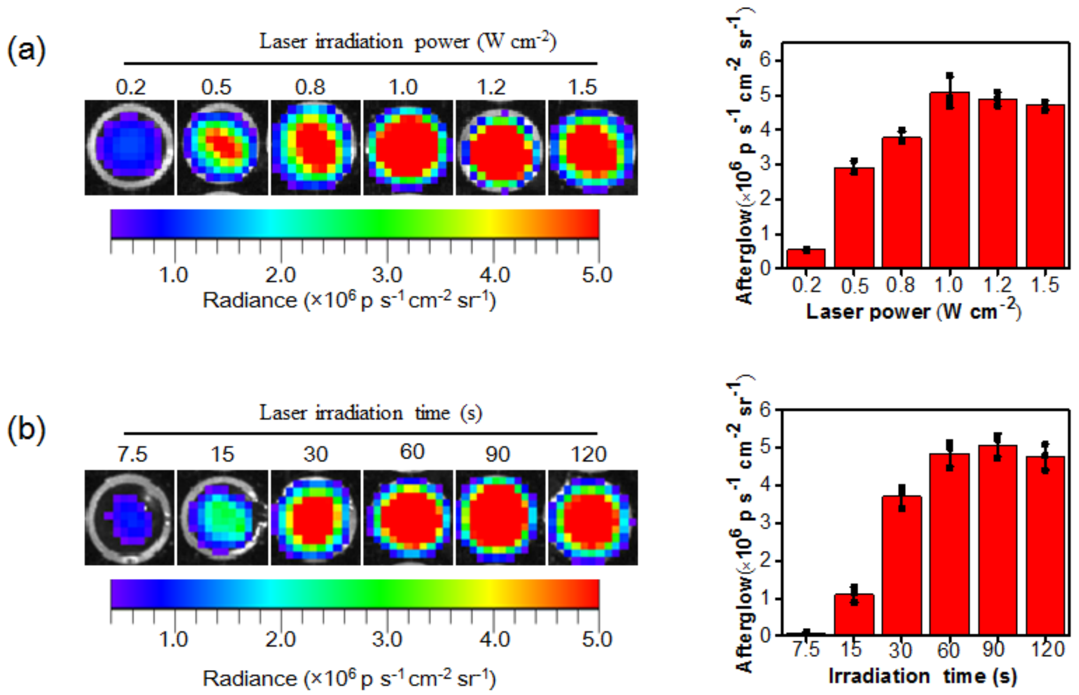

Supplementary Figure 11. Optimization of laser irradiation power and time for afterglow. (**a**) Afterglow images (left) and luminescence intensities (right) of **F1**$^{2+}$-ANP (58/28/2.2 μg mL^{-1} **F1**$^{2+}$(BF_4^-)$_2$/MEH-PPV/NIR775) in the presence of 200 μM NaHS, following by illumination with the 808 nm laser at different power densities for 1 min. (**b**) Afterglow images (left) and luminescence intensities (right) of H_2S-activated **F1**$^{2+}$-ANP following irradiation by the 808 nm laser (1 W cm^{-2}) for different time. The afterglow luminescence images were acquired for 60 s with an open filter after cessation of the laser. Values are mean ± s.d. (n = 3). The results demonstrate that irradiation of H_2S-activated **F1**$^{2+}$-ANP with the 808 nm laser at a power density of 1 W cm^{-2} for 1 min is the optimum condition for afterglow luminescence. Source data are provided as a Source Data file.

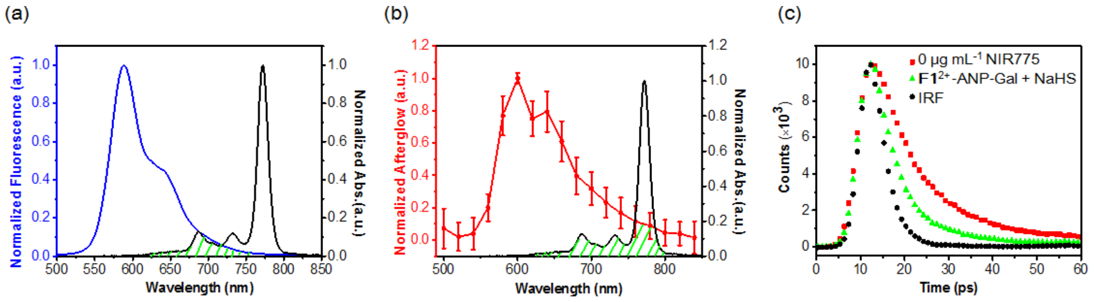

Supplementary Figure 12. The energy transfer process between MEH-PPV and NIR775. (**a**) Spectral overlap between the fluorescence of MEH-PPV (blue) and absorption of NIR775 (black). (**b**) Spectral overlap between the afterglow luminescence of MEH-PPV (red) and absorption of NIR775 (black). Values are mean ± s.d. (n = 3). Source data are provided as a Source Data file. (**c**) Fluorescence decay curves of MEH-PPV within H_2S-activated **F1**$^{2+}$-ANP (green) and H_2S-activated nanoparticles containing **F1**$^{2+}$(BF_4^-)$_2$/MEH-PPV (58/28 μg mL^{-1}) but without NIR775 (0 μg mL^{-1} NIR775) (red). Black dot line in **c** represents the internal reference (IRF).

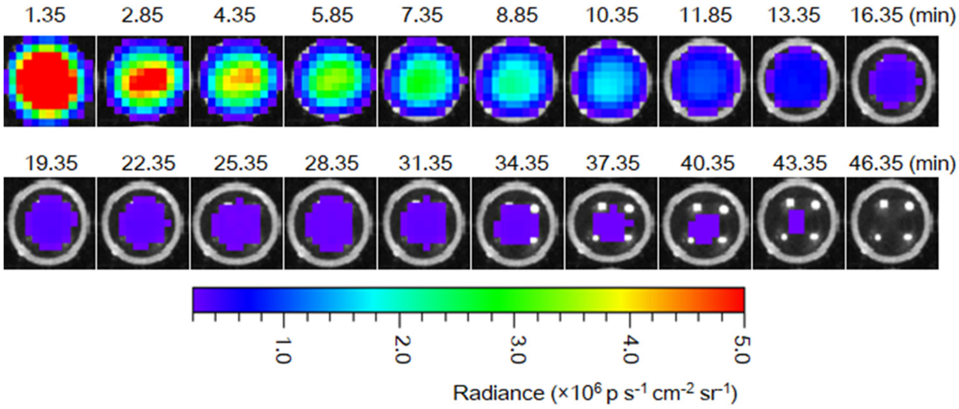

Supplementary Figure 13. Decay of afterglow images. **F1**$^{2+}$-ANP (58/28/2.2 μg mL^{-1} **F1**$^{2+}$(BF_4^-)$_2$/MEH-PPV/NIR775 in 200 μL PBS) was treated with 200 μM NaHS at 37 ºC for 1 min, and then irradiated by the 808 nm laser (1 W cm^{-2}) for 1 min. The afterglow images were acquired at indicated time after the end of laser irradiation. Each afterglow image was acquired for 60 s with an open filter.

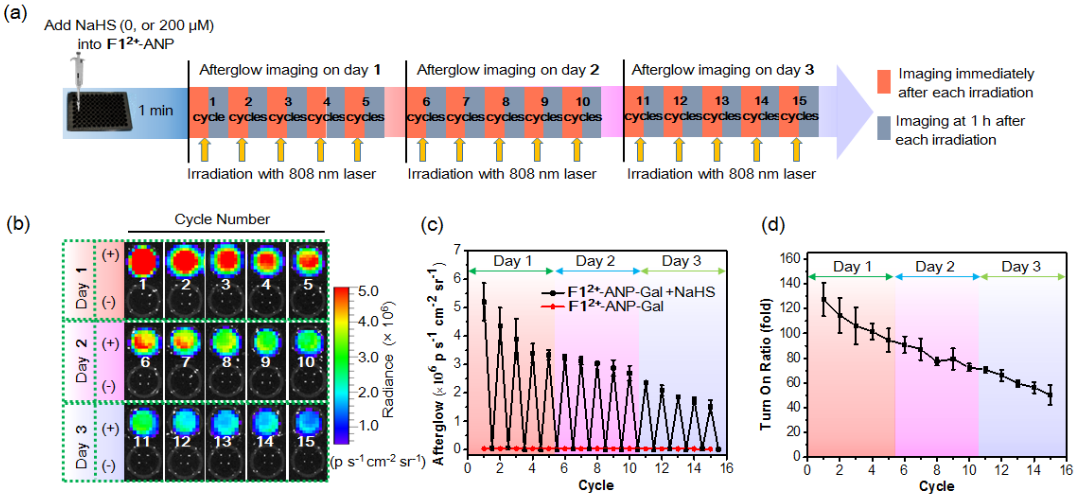

Supplementary Figure 14. Investigation of the ability to recharge afterglow. (**a**) Schematic illustration of the procedure to investigate the ability of **F1**$^{2+}$-ANP for long-term afterglow imaging. (**b**) Afterglow images and (**c**) intensities of **F1**$^{2+}$-ANP (-) and H_2S-activated **F1**$^{2+}$-ANP (+) following irradiation by 808 nm laser (1 W cm^{-2}, 1 min) for 15 cycles over 3 days. (**d**) Plot of the afterglow turn-on ratio of **F1**$^{2+}$-ANP toward NaHS following 15 cycle of irradiation over 3 days. Values are mean ± s.d. (n = 3). Briefly, on day 1, **F1**$^{2+}$-ANP (58/28/2.2 μg mL^{-1} **F1**$^{2+}$(BF_4^-)$_2$/MEH-PPV/NIR775) in PBS buffer were incubated without or with NaHS (200 μM) at 37 ºC for 1 min, and the solution was irradiated by the 808 nm laser (1 W cm^{-2}) for 1 min. The afterglow images were acquired immediately and at 1 h after the luminescence nearly decayed to background. Then, the solutions were irradiated with the 808 nm laser (1 W cm^{-2}, 1 min) for another 4 times, and the afterglow images were acquired immediately and at 1 h after each irradiation, under the same acquisition conditions. On day 2 and day 3, the experiment was performed on the same solution according to the procedure on day 1. Source data are provided as a Source Data file.

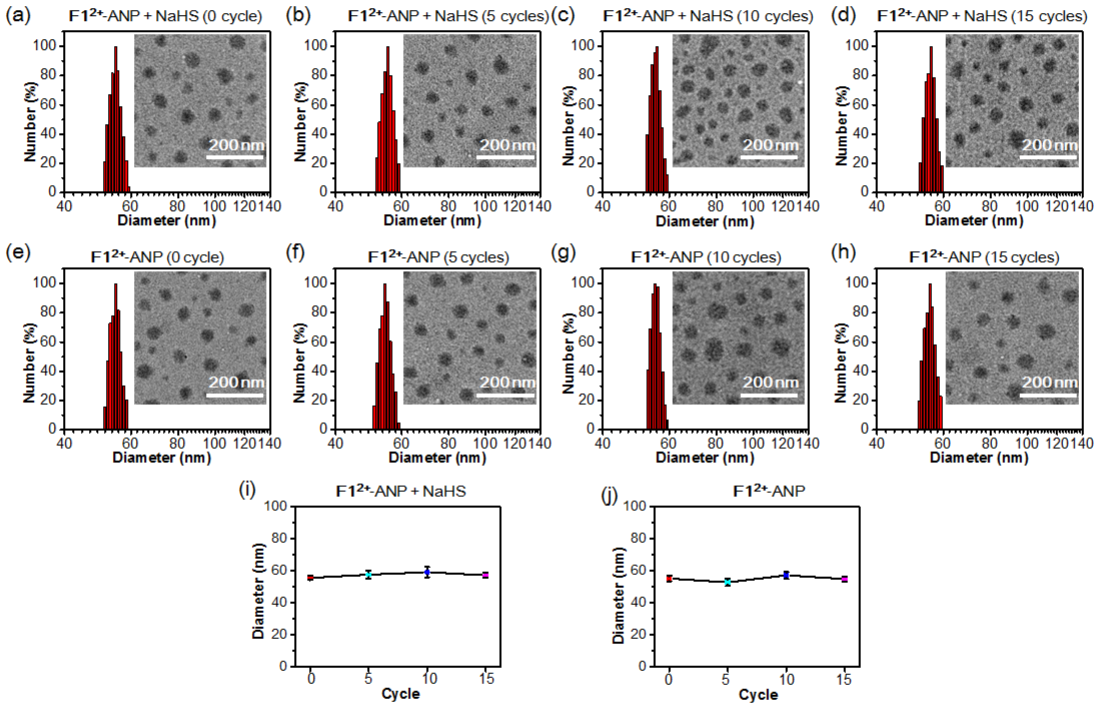

Supplementary Figure 15. Stability of **F1**$^{2+}$-ANP and activated **F1**$^{2+}$-ANP after irradiation. (**a-d**) DLS analysis and TEM image (inset) of **F1**$^{2+}$-ANP incubated with NaHS following irradiation by 808 nm laser (1 W cm^{-2}, 1 min) for 0, 5, 10 and 15 cycles over 3 days. (**e-h**) DLS analysis and TEM image (inset) of **F1**$^{2+}$-ANP alone following irradiation by 808 nm laser (1 W cm^{-2}, 1 min) for 0, 5, 10 and 15 cycles over 3 days. (**i**) Plot of the average hydrodynamic size of **F1**$^{2+}$-ANP incubated with NaHS following indicated irradiation cycles. **(j**) Plot of the average hydrodynamic size of **F1**$^{2+}$-ANP following indicated irradiation cycles. Values are mean ± s.d. (n = 3). Source data are provided as a Source Data file.

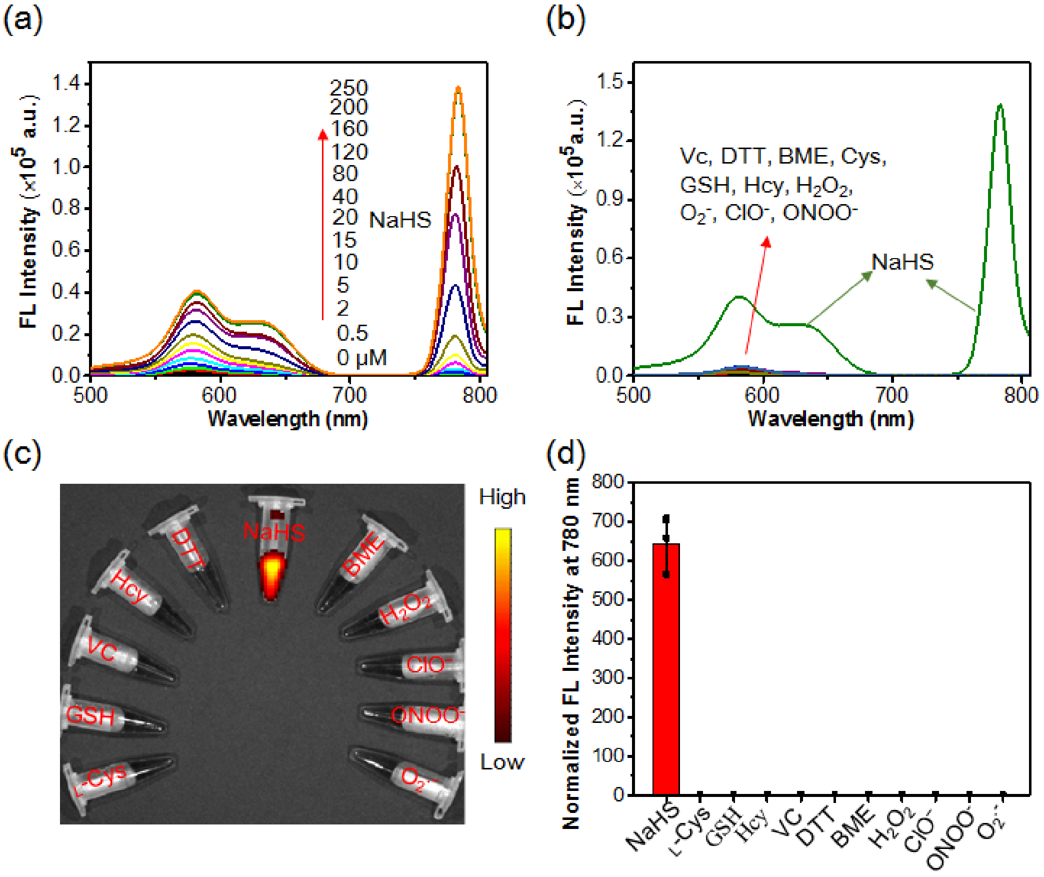

Supplementary Figure 16. Activation of the fluorescence of **F1**$^{2+}$-ANP toward H_2S. (**a**) Fluorescence spectra of **F1**$^{2+}$-ANP (58/28/2.2 μg mL^{-1} **F1**$^{2+}$(BF_4^-)$_2$/MEH-PPV/NIR775) following incubation with different concentrations of NaHS (0, 0.5, 2, 5, 10, 15, 20, 40, 80, 120, 160, 200, 250 μM) in PBS (1×, pH 7.4) at 37 ºC for 1 min. (**b**) Fluorescence spectra of **F1**$^{2+}$-ANP upon incubation with different reductants or ROS in PBS (1×, pH 7.4) at 37 ºC for 10 min. (**c**) Fluorescence images and (**d**) normalized FL intensities of **F1**$^{2+}$-ANP at 780 nm (λ_{ex} = 740 nm) following incubation with indicated agents (200 μM NaHS, 1.25 mM L-Cys, 10 mM GSH, 1 mM Hcy, 1.25 mM VC, 1.25 mM DTT, 100 μM BME, 1 mM H_2O_2, 1 mM ClO^- $ONOO^-$ (1 mM $NaNO_2$ + 1 mM H_2O_2) and $O_2^{\cdot-}$ (100 μM xanthine + 22 mU XO)). Values are mean ± s.d. (n = 3). Source data are provided as a Source Data file.

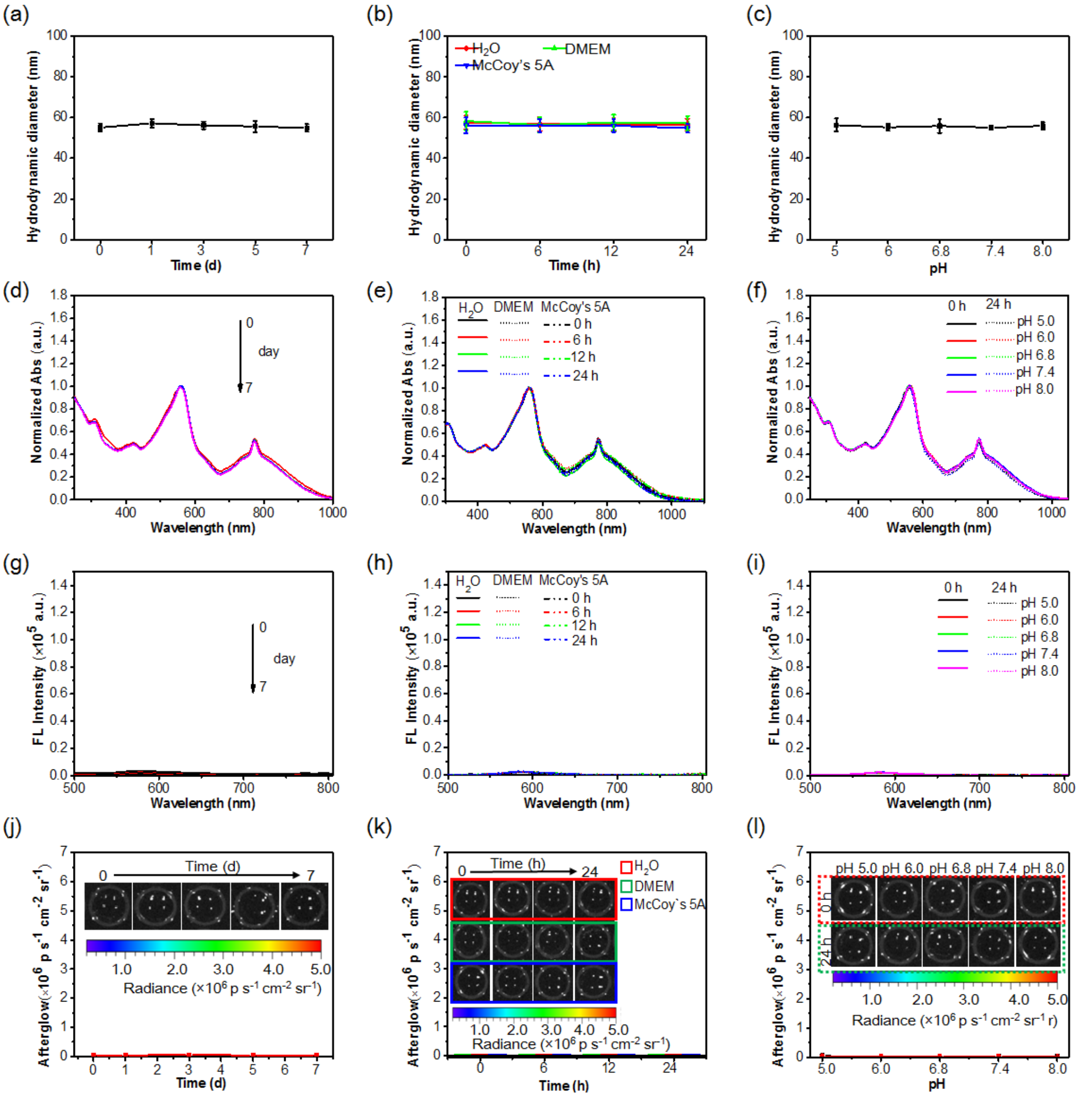

Supplementary Figure 17. Evaluation of the stability of **F1**$^{2+}$-ANP. (**a**) The hydrodynamic size of **F1**$^{2+}$-ANP following incubation in PBS buffer (1×, pH 7.4) at. r.t. for 7 days. (**b**) The hydrodynamic size of **F1**$^{2+}$-ANP following incubation in H_2O, DMEM or McCoy's 5A culture medium. (**c**) The hydrodynamic size of **F1**$^{2+}$-ANP following incubation in PBS buffer under indicated pH. (**d**) Absorption spectra of **F1**$^{2+}$-ANP following incubation in PBS buffer for 7 days. (**e**) Absorption spectra of **F1**$^{2+}$-ANP following incubation in indicated solution. (**f**) Absorption spectra of **F1**$^{2+}$-ANP following incubation in PBS buffer under indicated pH. (**g**) Fluorescence spectra of **F1**$^{2+}$-ANP following incubation in PBS buffer for 7 days. (**h**) Fluorescence spectra of **F1**$^{2+}$-ANP following incubation in indicated solution. (**i**) Fluorescence spectra of **F1**$^{2+}$-ANP following incubation in PBS buffer under indicated pH. (**j**) Afterglow intensities of **F1**$^{2+}$-ANP following incubation in PBS buffer for 7 days. Inset: afterglow images. (**k**) Afterglow intensities of **F1**$^{2+}$-ANP following incubation in indicated solution. Inset: afterglow images. (**l**) Afterglow intensities of **F1**$^{2+}$-ANP following incubation in PBS buffer under indicated pH. Inset: afterglow images. Values are mean ± s.d. (n = 3). Source data are provided as a Source Data file.

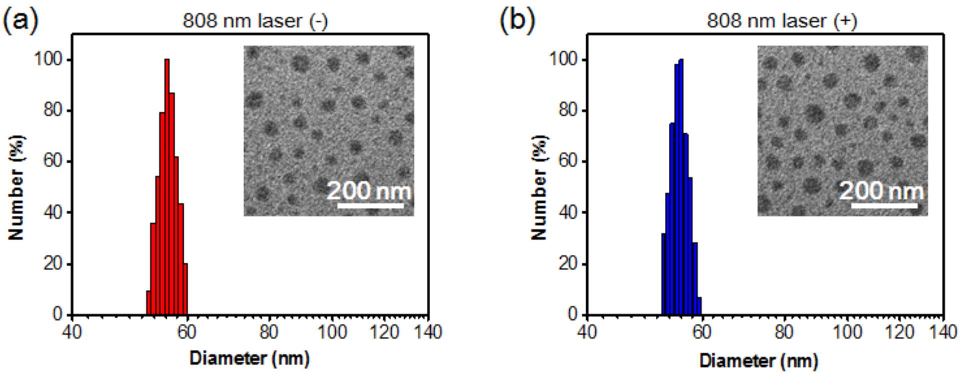

Supplementary Figure 18. Characterization of H_2S-activated **F1**$^{2+}$-ANP. (**a**) DLS analysis and TEM images (inset) of H_2S-activated **F1**$^{2+}$-ANP before irradiation. (**b**) DLS analysis and TEM images (inset) of H_2S-activated **F1**$^{2+}$-ANP with the 808 nm laser (1 W cm^{-2}, 1 min). Source data are provided as a Source Data file.

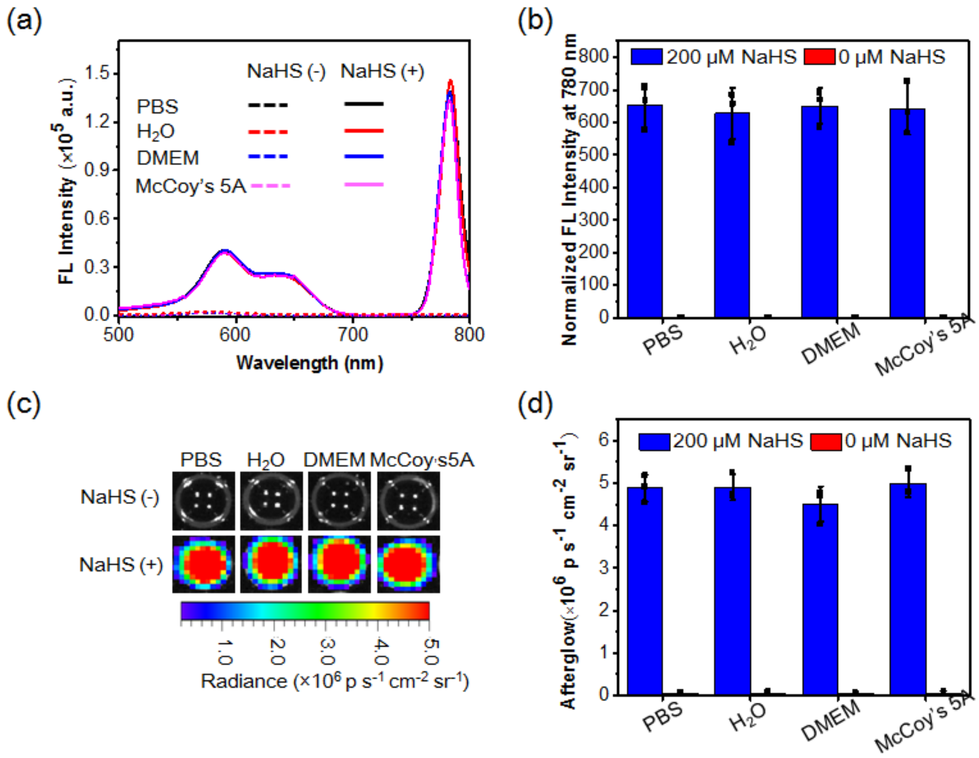

Supplementary Figure 19. Response of **F1**$^{2+}$-ANP toward H_2S. (**a**) Comparison of the fluorescence spectra of **F1**$^{2+}$-ANP (58/28/2.2 μg mL^{-1} **F1**$^{2+}$$(BF_4^-)_2$/MEH-PPV/NIR775) upon incubation with or without 200 μM NaHS in PBS (1×, pH 7.4), H_2O, DMEM or McCoy's 5A culture medium for 1 min. (**b**) Normalized FL intensities at 780 nm. (**c**) Comparison of the afterglow images of **F1**$^{2+}$-ANP before and after incubation with 200 μM NaHS in indicated solution for 1 min. (**d**) Afterglow intensities of indicated solutions. The solutions were irradiated with the 808 nm laser (1 W cm^{-2}) for 1 min. After cessation of laser, the afterglow images were acquired immediately under bioluminescence mode, with an acquisition time of 60 s. Values are mean ± s.d. (n = 3). Source data are provided as a Source Data file.

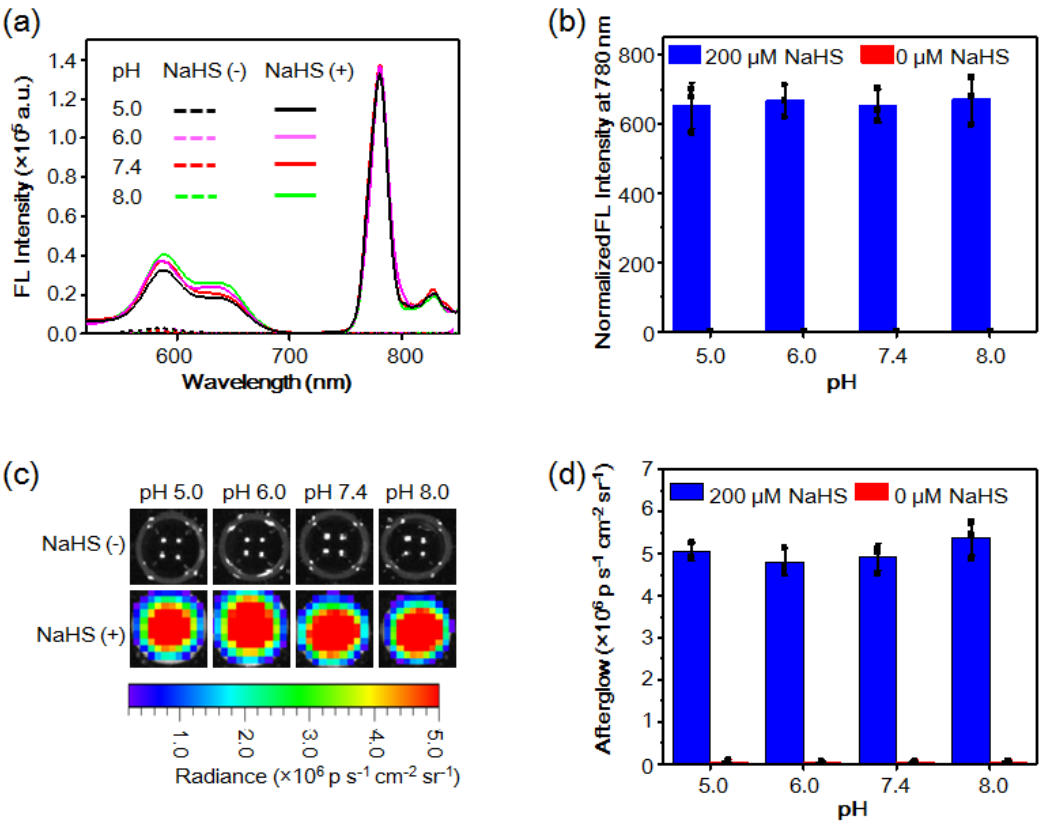

Supplementary Figure 20. Effect of pH on the activation of **F1**$^{2+}$-ANP toward H_2S. (**a**) Fluorescence spectra of **F1**$^{2+}$-ANP (58/28/2.2 µg mL^{-1} **F1**$^{2+}$(BF_4^-)$_2$/MEH-PPV/NIR775) in the absence or presence of 200 µM NaHS in PBS buffer at different pH. (**b**) Normalized FL intensities at 780 nm. (**c**) Afterglow images of **F1**$^{2+}$-ANP before and after incubation with 200 µM NaHS in PBS buffer at indicated pH. (**d**) Afterglow intensities of **F1**$^{2+}$-ANP before and after incubation with 200 µM NaHS in PBS buffer at indicated pH. The solutions were irradiated with the 808 nm laser (1 W cm^{-2}) for 1 min. After cessation of laser, the afterglow images were acquired immediately under bioluminescence mode, with an acquisition time of 60 s. Values are mean ± s.d. (n = 3). Source data are provided as a Source Data file.

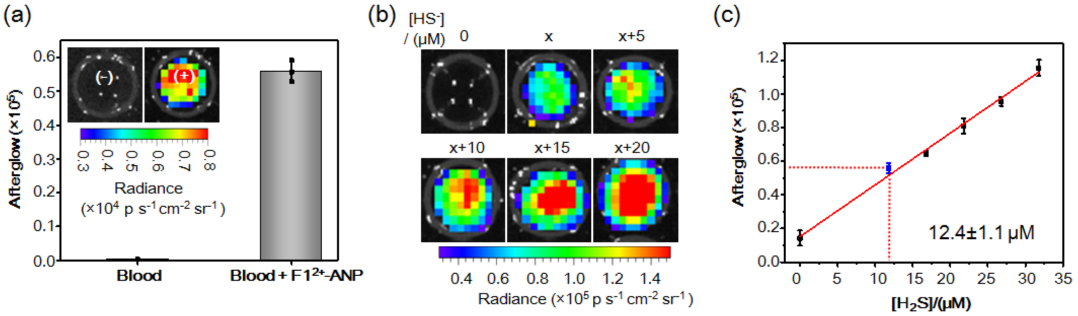

Supplementary Figure 21. Measurement of H_2S in mouse blood. (**a**) Afterglow luminescence images (inset) and intensities of 2-fold diluted mouse blood or 2-fold diluted mouse blood incubating with **F1**$^{2+}$-ANP (58/28 μg mL^{-1} **F1**$^{2+}$/ MEH-PPV) at 37 °C for 1 min. (**b**) Afterglow images of **F1**$^{2+}$-ANP (58/28/2.2 μg mL^{-1} **F1**$^{2+}$(BF_4^-)$_2$/MEH-PPV/NIR775) incubating in 2-fold diluted mouse blood with the addition of 0, 5, 10, 15 and 20 μM NaHS at 37 °C for 1 min. Each sample was irradiated under the 808 nm laser (1 W cm^{-2}) for another 1 min. After removal of the laser, the afterglow luminescence images were immediately acquired with a 790 nm emission filter, with an acquisition time of 60 s. (**c**) Plot of the afterglow intensity and HS^- concentration (0, x, x + 5, x + 10, x + 15, and x + 20) to determine the H_2S concentration in mouse blood. The 0 point was obtained by adding $ZnCl_2$ into mouse blood to trap sulfide. Values are mean ± s.d. (n = 3). The reuslts show that blood alone demonstrated nearly no afterglow signals in the absence of real-time excitation, while incubation with **F1**$^{2+}$-ANP for only 1 min produced obvious afterglow luminescence. Adding NaHS into the blood as an internal standard led to an H_2S concentration in two-fold diluted mouse blood of approximately 12.4 ± 1.1 μM; thus, the H_2S concentration in whole mouse blood was 24.8 ± 2.2 μM. Source data are provided as a Source Data file.

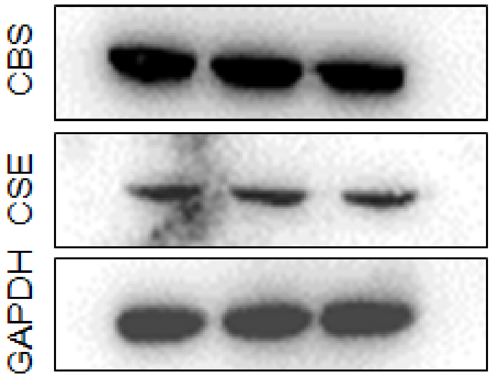

Supplementary Figure 22. Western Blot analysis of CBS and CSE. The result shows the expression of CBS and CSE in HepG2 tumor cells. Source data are provided as a Source Data file.

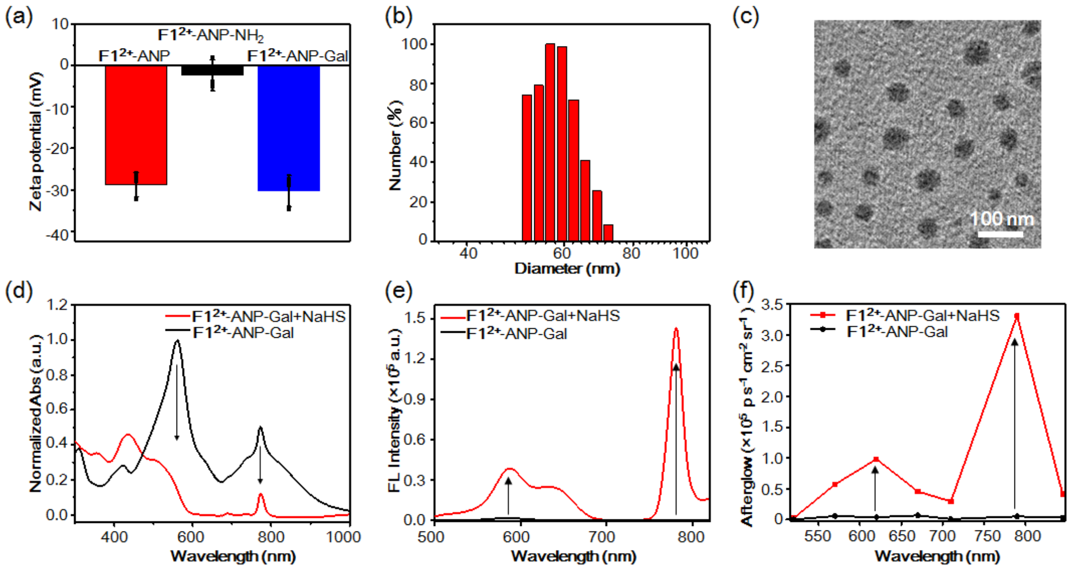

Supplementary Figure 23. Characterization of **F1**$^{2+}$-ANP-Gal in vitro. (**a**) Zeta potential of **F1**$^{2+}$-ANP, **F1**$^{2+}$-ANP -NH_2 and **F1**$^{2+}$-ANP-Gal in water. Values are mean ± s.d. (n = 3). (**b**) DLS analysis shows the average hydrodynamic diameter of **F1**$^{2+}$-ANP-Gal in PBS buffer (1×, pH 7.4). (**c**) TEM image of **F1**$^{2+}$-ANP-Gal. (**d**) UV-vis-NIR spectra of **F1**$^{2+}$-ANP-Gal before and after incubation with NaHS (200 μM) in PBS buffer (1×, pH 7.4) for 1 min. (**e**) Fluorescence and afterglow luminescence (**f**) spectra of **F1**$^{2+}$-ANP-Gal (58/28/2.2 μg mL^{-1} **F1**$^{2+}$(BF_4^-)$_2$/MEH-PPV/NIR775) before and after incubation with NaHS (200 μM) in PBS buffer (1×, pH 7.4) at 37 ºC for 1 min . Fluorescence spectra was acquired by synchronous fluorescence scanning (λ_{ex} = 400-800 nm, offset = 100 nm). For afterglow luminescence spectra, the incubation solutions were irradiated with the 808 nm laser (1 W cm^{-2}) for 1 min. After cessation of laser, the afterglow images were acquired under indicated filters, with an acquisition time of 60 s. Source data are provided as a Source Data file.

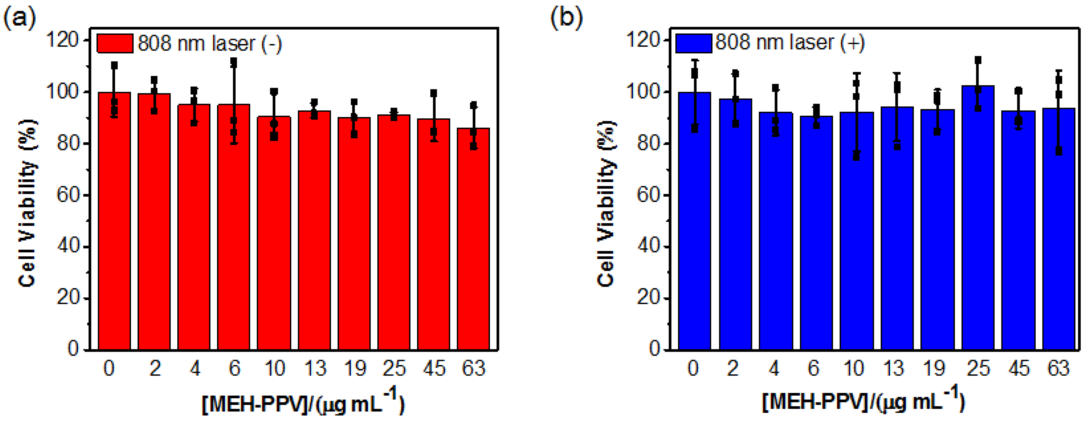

Supplementary Figure 24. Evaluation of biocompatibility of **F1**$^{2+}$-ANP-Gal in living cells. The cytotoxicity of **F1**$^{2+}$-ANP-Gal (**a**) and **F1**$^{2+}$-ANP-Gal together with 808 nm laser irradiation (**b**) against HepG2 cells. Cells were incubated with indicated concentration of **F1**$^{2+}$-ANP-Gal (based on MEH-PPV) for 24 h, and irradiated in the absence and presence of 808 nm laser (1 W cm^{-2}) for 1 min. The cytotoxicity was test by MTT assay. Values are mean ± s.d. (n = 3). Source data are provided as a Source Data file.

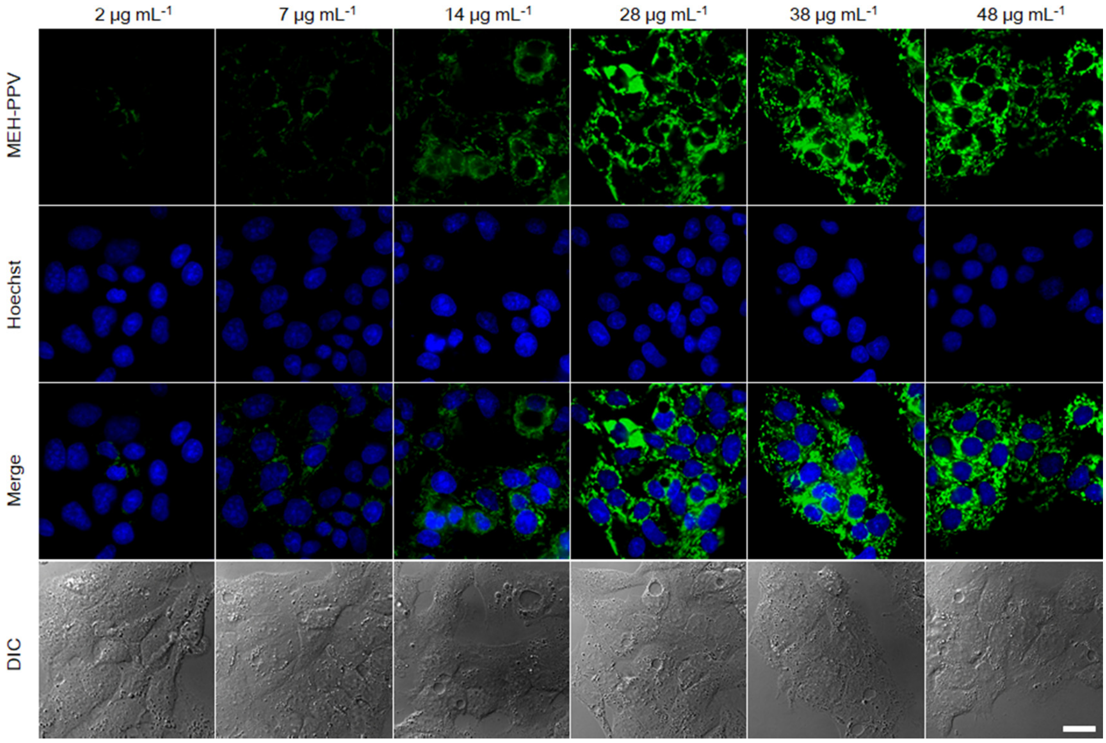

Supplementary Figure 25. Concentration-dependent fluorescence images of HepG2 cells. HepG2 cells were incubated with **F1**$^{2+}$-ANP-Gal (2, 7, 14, 28, 38, 48 μg mL^{-1} based on MEH-PPV) for 3 h, washed, and then stained with 2.0 μM Hoechst for 20 min. Scale bar: 20 μm.

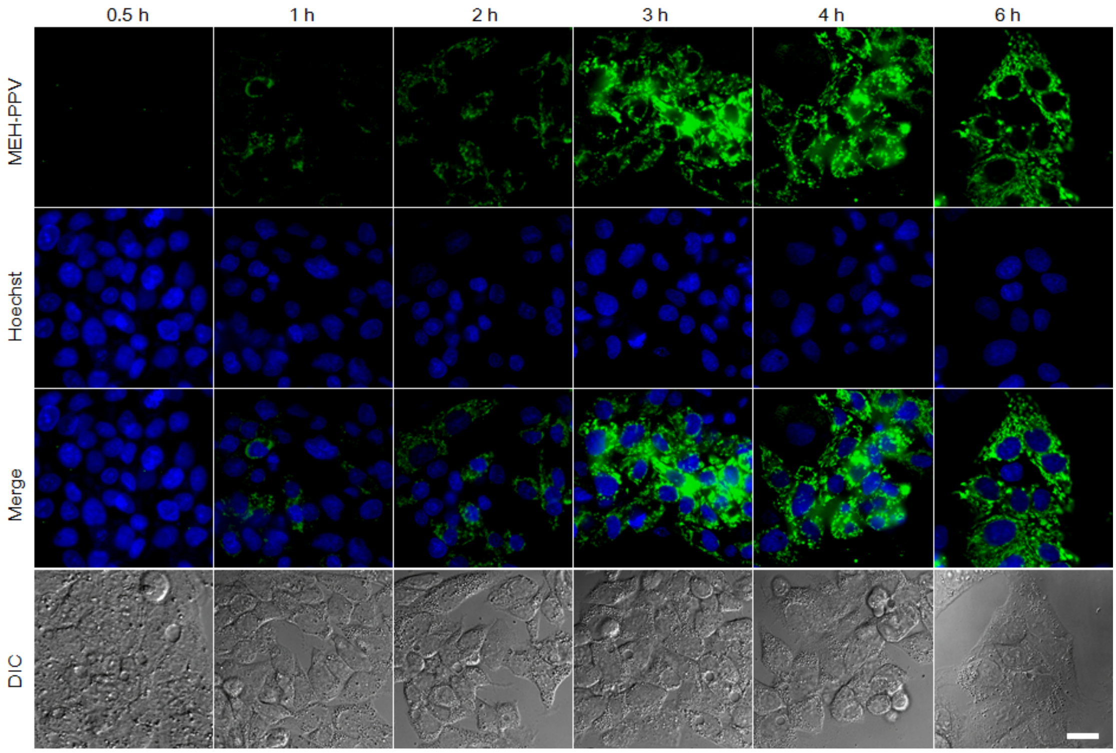

Supplementary Figure 26. Time-dependent fluorescence images of HepG2 cells. HepG2 cells were incubated with **F1**$^{2+}$-ANP-Gal (58/28/2.2 μg mL^{-1} **F1**$^{2+}$(BF_4^-)$_2$/MEH-PPV/NIR775) for 0.5, 1, 2, 3, 4, 6 h, washed, and then stained with 2.0 μM Hoechst for 20 min. Scale bar: 20 μm.

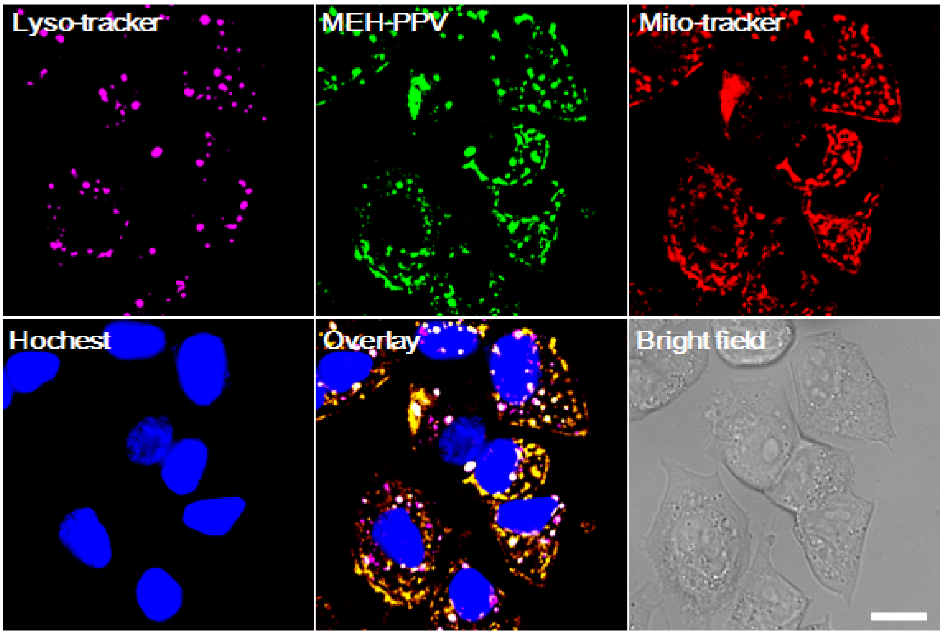

Supplementary Figure 27. Colocalization study of **F1**$^{2+}$-ANP-Gal in HepG2 cells. HepG2 cells were incubated with **F1**$^{2+}$-ANP-Gal (58/28/2.2 μg mL^{-1} **F1**$^{2+}$(BF_4^-)$_2$/MEH-PPV/NIR775) for 3 h, washed, and then incubated with 2 μM Lyso-tracker (Magenta) or 200 nM Mitro-Tracker (Red) and 2.0 μM nucleus staining dye (Hoechst 33342, blue) for 20 min. Scale bar: 20 μm. The fluorescence images revealed that **F1**$^{2+}$-ANP-Gal could accumulated in both lysosomes and mitochondria of HepG2 cells.

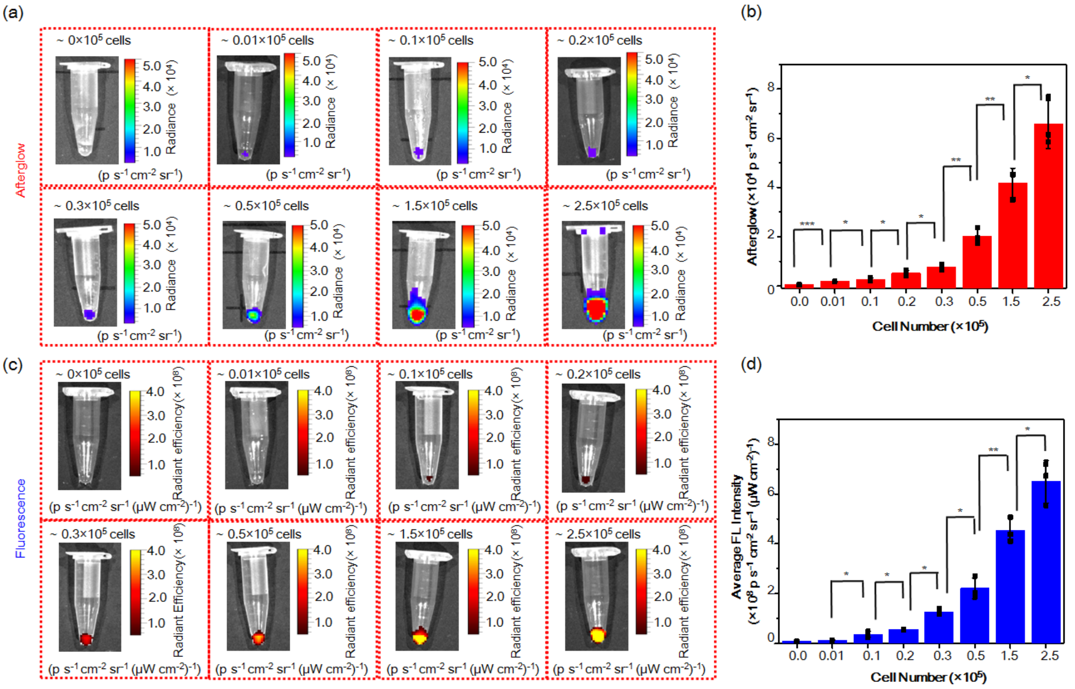

Supplementary Figure 28. Detection of endogenous H_2S in HepG2 cell pellets. Representative afterglow (**a**) and fluorescence images (**c**) of **F1^{2+}**-ANP-Gal-loading HepG2 cell pellets with varying cell number (0, 1×10^3, 1×10^4, 2×10^4, 3×10^4, 5×10^4, 1.5×10^5, 2.5×10^5). Comparison of the afterglow (**b**) and fluorescence intensities (**d**) of HepG2 cells at indicated number. Data are mean ± s.d. (*$P < 0.05$, **$P < 0.01$, ***$P < 0.001$, $n = 3$). HepG2 cells were incubated with **F1^{2+}**-ANP-Gal (58/28/2.2 μg mL^{-1} **F1^{2+}**$(BF_4^-)_2$/MEH-PPV/NIR775) for 3 h. Then, the incubation medium was removed, washed with cold PBS buffer for three times. The cell pellets were collected after trypsinization, washed, and irradiated with the 808 nm laser (1W cm^{-2}) for 1 min. After cessation of laser, the afterglow images were acquired for 60 s, with an open filter. The fluorescence images were acquired with $\lambda_{ex}/\lambda_{em} = 740/790$ nm. Statistical differences were analyzed by Student's t-test. Source data are provided as a Source Data file.

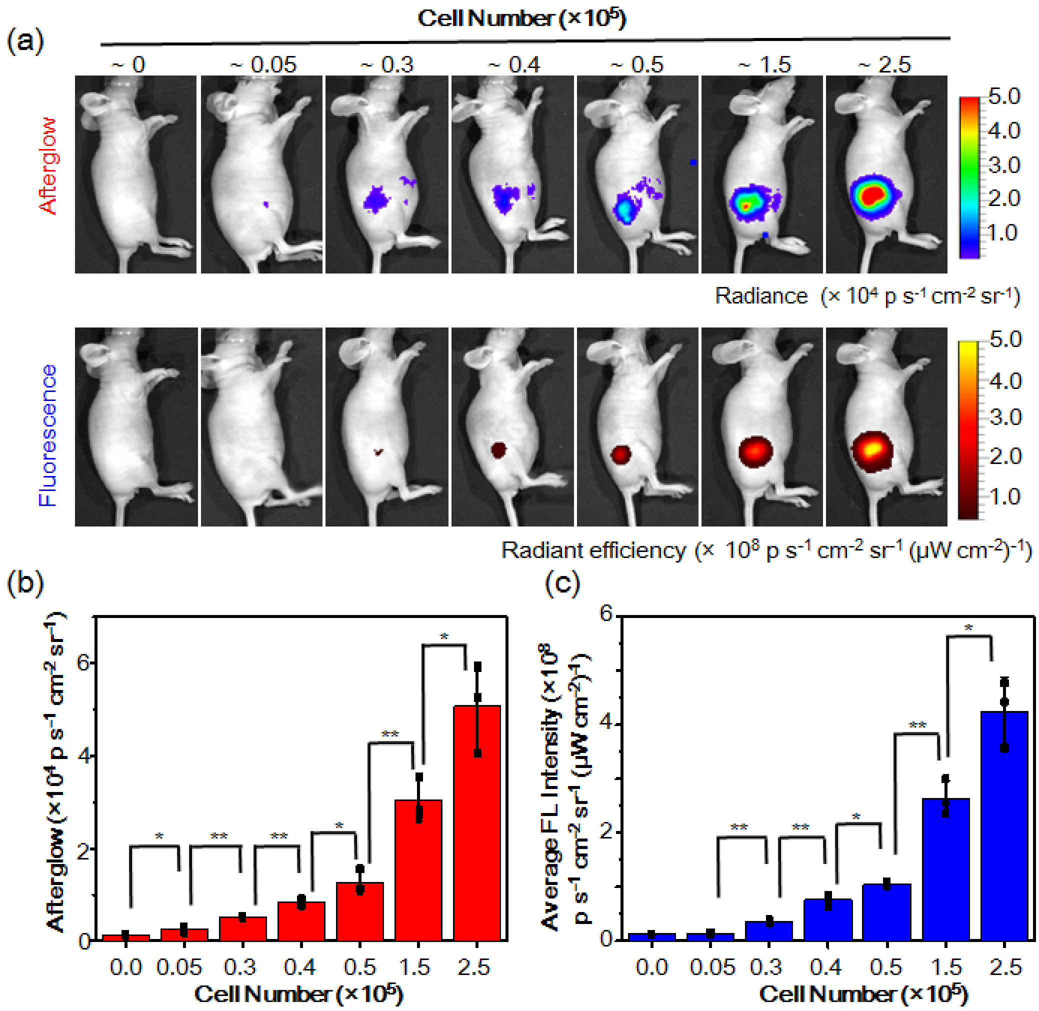

Supplementary Figure 29. Detection of endogenous H_2S in HepG2 cells in vivo. (**a**) Afterglow and fluorescence images of mice subcutaneously injected with different number of HepG2 cells (0, 5×10^3, 3×10^4, 4×10^4, 5×10^4, 1.5×10^5 and 2.5×10^5). Comparison of the afterglow (**b**) and fluorescence intensities (**c**) of HepG2 cells at indicated number. Data are mean ± s.d. (*P < 0.05, **P < 0.01, n = 3). Cells mixed with **F1**$^{2+}$-ANP-Gal (2.2/28/58 μg mL^{-1} NIR775/MEH-PPV/**F1**$^{2+}$(BF_4^-)$_2$) were implanted in the right thigh. After 3 h, the cells were irradiated with the 808 nm laser (1 W cm^{-2}) for 1 min. After cessation of laser, the afterglow images were acquired for 60 s, with an open filter. The fluorescence images were acquired with $\lambda_{ex}/\lambda_{em}$ = 740/790 nm. Statistical differences were analyzed by Student's t-test. Source data are provided as a Source Data file.

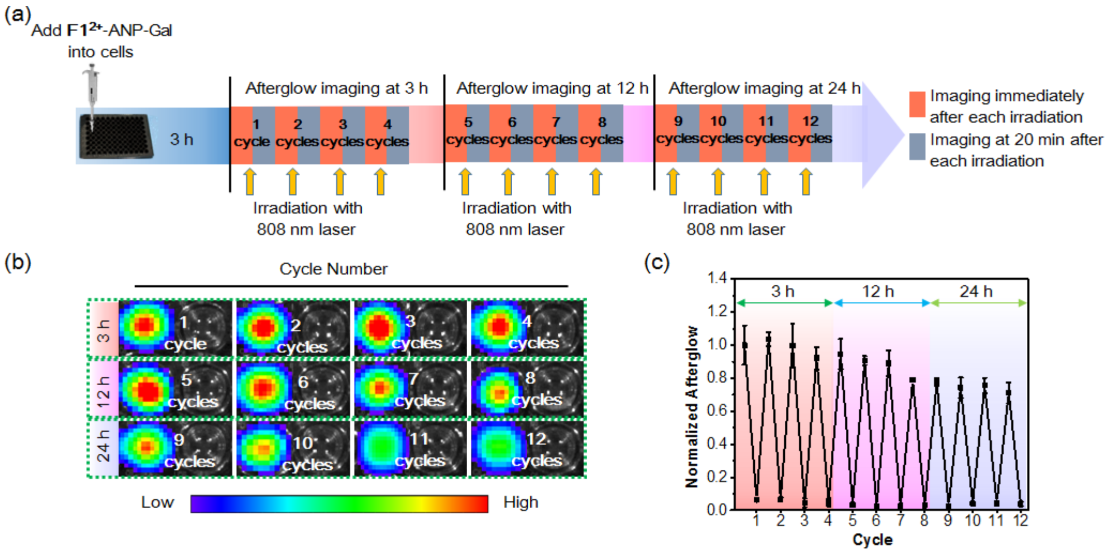

Supplementary Figure 30. Ability of **F1**$^{2+}$-ANP-Gal for long-term afterglow imaging. (**a**) Schematic illustration of the procedure to investigate the ability of **F1**$^{2+}$-ANP-Gal for long-term afterglow imaging in living HepG2 cells. (**b**) Afterglow images and (**c**) average afterglow luminescence intensities of HepG2 cells incubated with **F1**$^{2+}$-ANP-Gal, following by irradiation with the 808 nm laser (1 W cm^{-2}, 1 min) for 12 cycles over 24 h. Values are mean ± s.d. (n = 3). Source data are provided as a Source Data file.

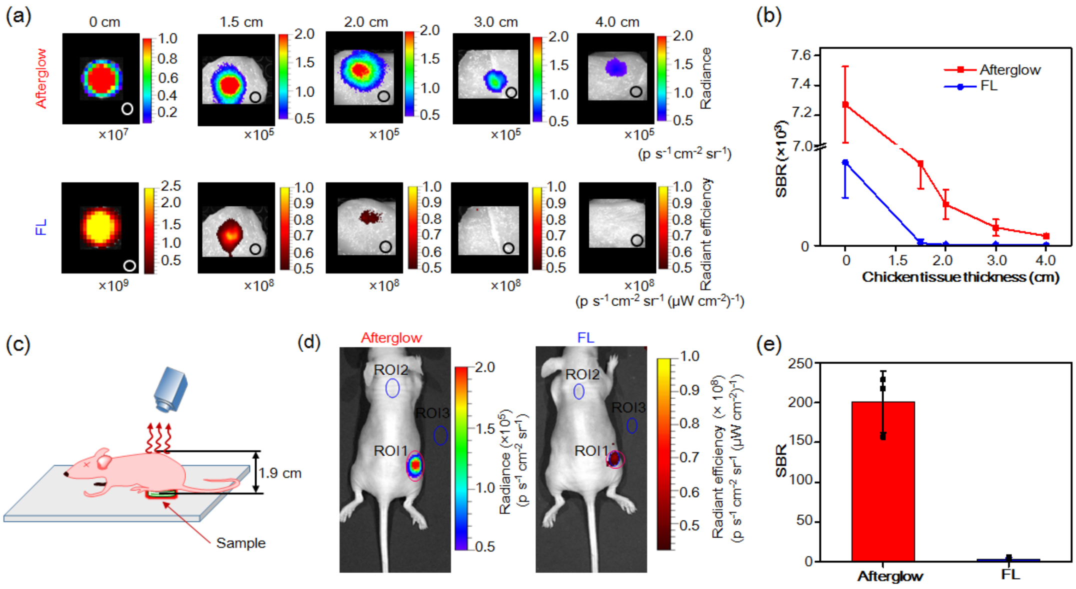

Supplementary Figure 31. Tissue penetration study of H_2S-activated **F1**$^{2+}$-ANP-Gal. (**a**) Afterglow luminescence (up) and fluorescence (down) images of the solution of **F1**$^{2+}$-ANP-Gal (0.684/0.33/0.026 mg mL^{-1} **F1**$^{2+}$$(BF_4^-)_2$/MEH-PPV/NIR775, 200 µL) in the presence of NaHS (2 mM), which was covered with chicken tissues of different thickness (0, 1.5, 2.0, 3.0 and 4.0 cm). (**b**) Plot of the SBRs for afterglow luminescence (red) and fluorescence (blue) images as a function of chicken tissue thickness. Circles indicate the location of background chosen for SBRs. (**c**) Schematic illustration of afterglow luminescence imaging through a living mouse body, where the H_2S-activated **F1**$^{2+}$-ANP-Gal is located under the mouse body with a thickness of ~1.9 cm. (**d**) Afterglow luminescence (left) and fluorescence (right) images of the H_2S-activated **F1**$^{2+}$-ANP-Gal through a living mouse body. A solution of **F1**$^{2+}$-ANP-Gal (0.684/0.33/0.026 mg mL^{-1} **F1**$^{2+}$$(BF_4^-)_2$/MEH-PPV/NIR775, 50 µL) in the presence of NaHS (2 mM) were pre-irradiated with the 808 nm laser (1 W cm^{-2}, 1 min), and then placed under the mouse body. The afterglow images were collected for 60 s with an open filter. The fluorescence images were acquired with $\lambda_{ex}/\lambda_{em}$ = 740/790 nm. (**e**) SBRs for afterglow luminescence and fluorescence imaging of H_2S-activated **F1**$^{2+}$-ANP-Gal. SBR = [(afterglow signal, ROI 1) - (background 2, ROI 3)] / [(background 1, ROI 2) - (background 2, ROI 3)]. The results show that the afterglow signals of H_2S-activated **F1**$^{2+}$-ANP-Gal hold much higher ability to penetrate into biological tissues than NIR fluorescence much deeper. Values are mean ± s.d. (n=3). Source data are provided as a Source Data file.

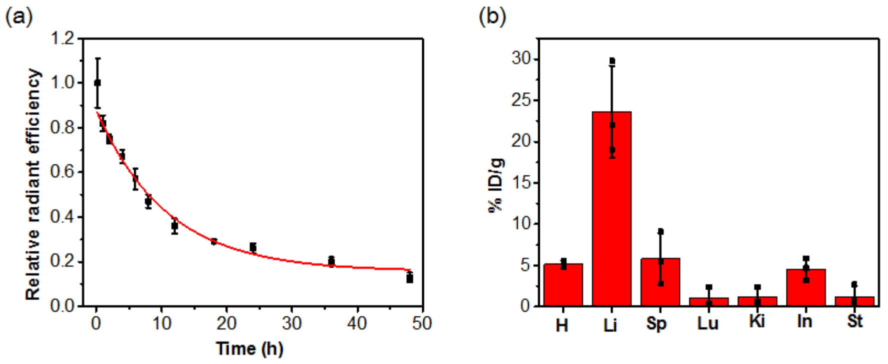

Supplementary Figure 32. Blood circulation and biodistribution of **F1**$^{2+}$-ANP-Gal. (**a**) Blood circulation curve of **F1**$^{2+}$-ANP-Gal in mice. The blood half-life ($t_{1/2}$) of **F1**$^{2+}$-ANP-Gal was measured to be ~7.5 h after i.v. administration into mice. (**b**) Biodistribution of **F1**$^{2+}$-ANP-Gal (211/100/8 μg **F1**$^{2+}$(BF_4^-)$_2$/MEH-PPV/NIR775, 200 μL) at 12 h post i.v. injection into mice. Values are mean ± s.d. (n = 3). The %ID/g in liver was found to be ~23.6%, much higher compared to that in other organs, suggesting that **F1**$^{2+}$-ANP-Gal was mainly accumulated in liver at 12 h post i.v. injection. Source data are provided as a Source Data file.

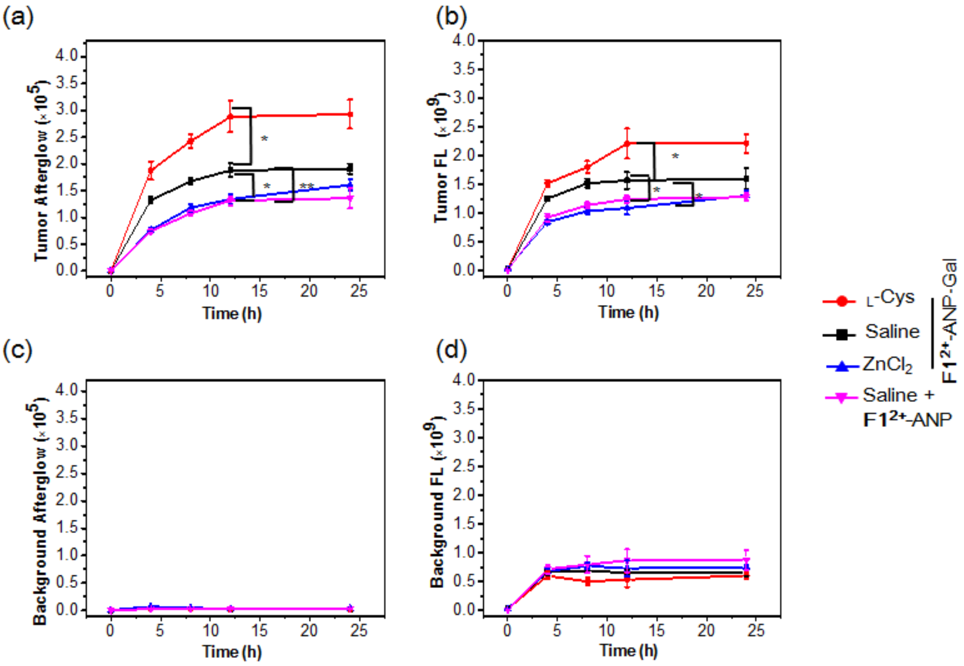

Supplementary Figure 33. Afterglow and fluorescence intensities at different time. Plots of the average (**a**) afterglow and FL (**b**) intensity of the HepG2 tumors in mice at 0, 4, 8, 12 and 24 h following i.v. injection of **F1**$^{2+}$-ANP-Gal or **F1**$^{2+}$-ANP in saline (saline), **F1**$^{2+}$-ANP-Gal with i.t. injection of L-Cys or $ZnCl_2$. L-Cys (1 mM, 25 μL) or $ZnCl_2$ (1 mM, 25 μL) was injected into tumors at 3.5 h post i.v. injection of **F1**$^{2+}$-ANP-Gal (211/100/8 μg **F1**$^{2+}$(BF_4^-)$_2$/MEH-PPV/NIR775, 200 μL). Plots of the average afterglow (**c**) and FL (**d**) intensity of the background in HepG2 tumor bearing mice following indicated treatment. The background locations in mice were shown in Fig. 4b. Values are mean ± s.d. (* $P < 0.05$, ** $P < 0.01$, n = 3). Statistical differences were analyzed by Student's t-test. Source data are provided as a Source Data file.

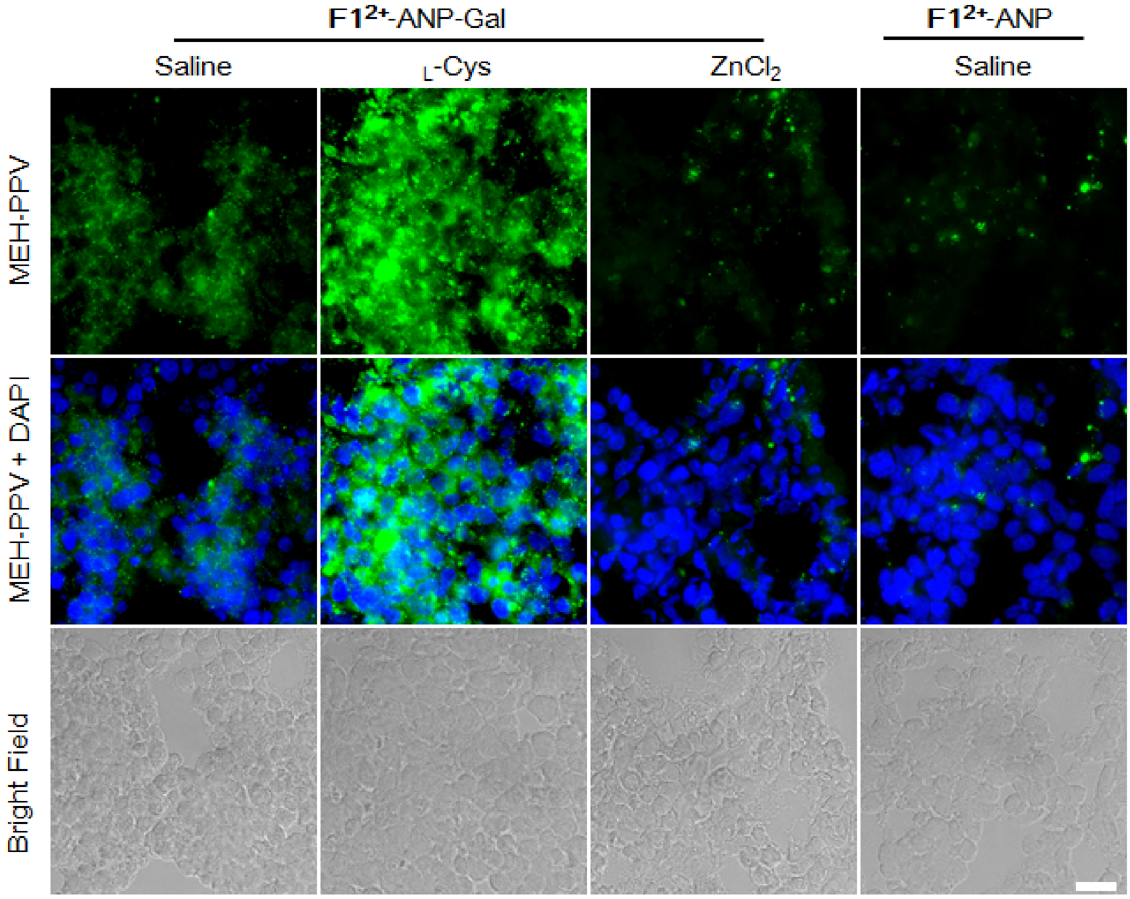

Supplementary Figure 34. Fluorescence imaging of HepG2 tumor tissue slices. HepG2 tumor-bearing mice were i.v. injected with **F1**$^{2+}$-ANP-Gal or **F1**$^{2+}$-ANP (211/100/8 µg **F1**$^{2+}$(BF_4^-)$_2$/MEH-PPV/NIR775 in 200 µL saline), **F1**$^{2+}$-ANP-Gal plus L-Cys (i.t), or **F1**$^{2+}$-ANP-Gal plus $ZnCl_2$ (i.t). The mice were sacrificed at 12 h, and the tumors were dissected. The tumor tissues were then cut to obtain 10 µm-thickness slices. After staining with DAPI, the tumor tissue slices were imaged with the IX73 optical microscope equipped with DAPI and Cy3 filters. Scale bar: 20 µm.

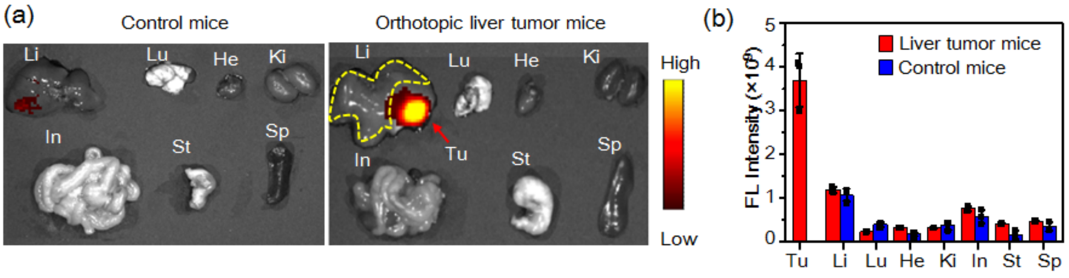

Supplementary Figure 35. Evaluation of the biodistribution of **F1**$^{2+}$-ANP-Gal ex vivo. (**a**) Representative ex vivo fluorescence images and (**b**) average FL intensities of main organs resected from control mice and orthotopic HepG2 tumor bearing mice at 12 h post i.v. injection of **F1**$^{2+}$-ANP-Gal (211/100/8 µg **F1**$^{2+}$(BF_4^-)$_2$/MEH-PPV/NIR775, 200 µL). Tu: tumor, Li: liver, Lu: lung, He: heart, Ki: kidneys, In: intestines, St: stomach, Sp: spleen. Red arrow indicates the location of HepG2 tumor in the liver, and the yellow box indicates the location chosen for ROI of liver. The fluorescence images were acquired with $\lambda_{ex/em}$ = 740/790 nm. Values are mean ± s.d. (n = 3). Source data are provided as a Source Data file.

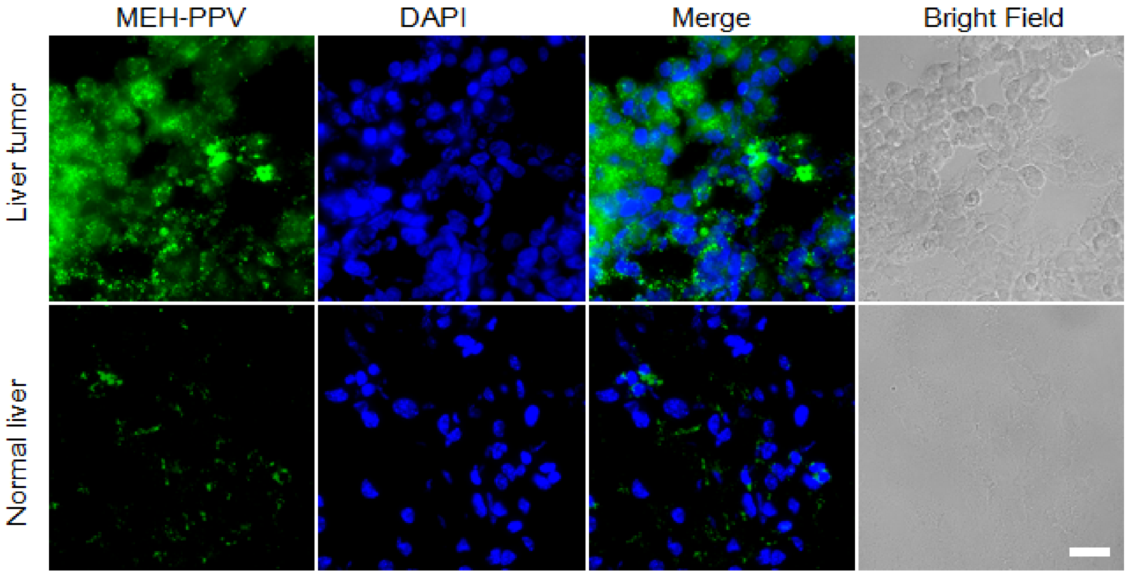

Supplementary Figure 36. Fluorescence imaging of HepG2 tumor and liver tissue slices. The orthotopic HepG2 tumor-bearing mice and control mice were i.v. injected with **F1**$^{2+}$-ANP-Gal (211/100/8 µg **F1**$^{2+}$(BF_4^-)$_2$/MEH-PPV/NIR775 in 200 µL saline). After 12 h, the mice were sacrificed, and the tumors from orthotopic HepG2 tumor mice and normal liver from control mice were dissected. The tumor and normal liver tissues were then cut to obtain 10 µm-thickness slices. After staining with DAPI, the tissue slices were imaged with the IX73 optical microscope equipped with DAPI and Cy3 filters. Scale bar: 20 µm.

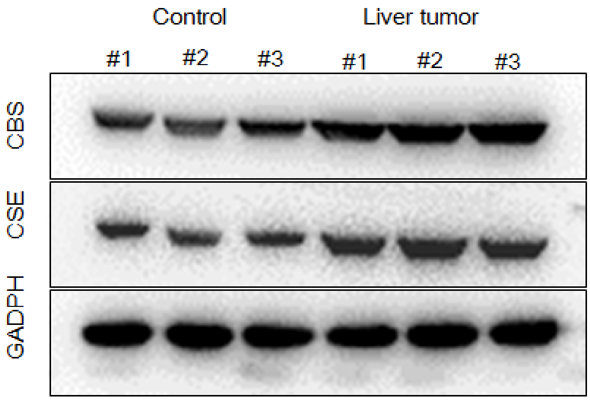

Supplementary Figure 37. Western Bolt analysis of CBS and CSE. The result shows the comparison of CBS and CSE expression in the liver tissues from control mice and tumor tissues from orthotopic liver tumor mice. #1, #2 and #3 indicate the number of three mice used for the analysis. Source data are provided as a Source Data file.

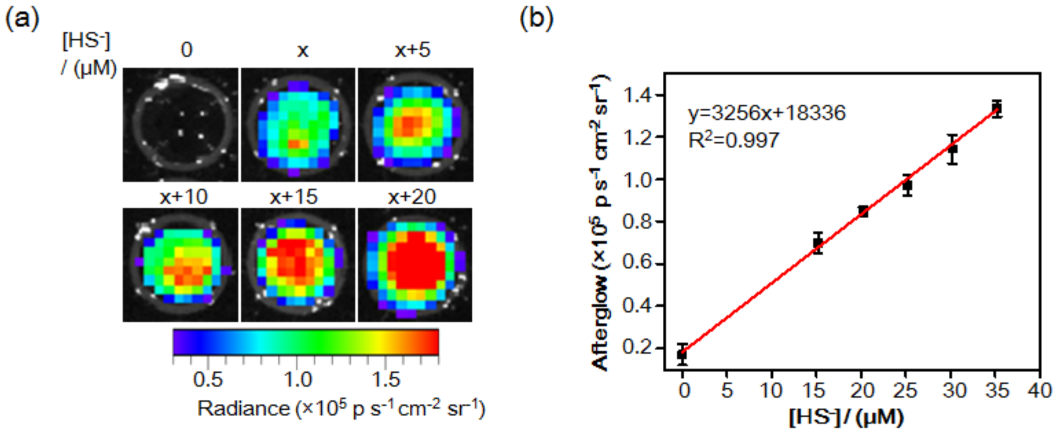

Supplementary Figure 38. A standard curve established by adding NaHS into blood. (**a**) Afterglow images of **F1**$^{2+}$-ANP (58/28/2.2 μg mL^{-1} **F1**$^{2+}$(BF_4^-)$_2$/MEH-PPV/NIR775) incubating in 2-fold diluted healthy persons' blood with the addition of 0, 5, 10, 15 and 20 μM NaHS at 37 °C for 1 min. Each sample was irradiated under the 808 nm laser (1 W cm^{-2}) for another 1 min. After removal of the laser, the afterglow luminescence images were immediately acquired with a 790 nm emission filter, with an acquisition time of 60 s. (**b**) Plot of the afterglow intensity and HS^- concentration (0, x, x + 5, x + 10, x + 15, and x + 20) to determine the H_2S concentration in healthy persons' blood. The 0 point was obtained by adding $ZnCl_2$ into healthy persons' blood to trap sulfide. Values are mean ± s.d. (n = 3). Source data are provided as a Source Data file.

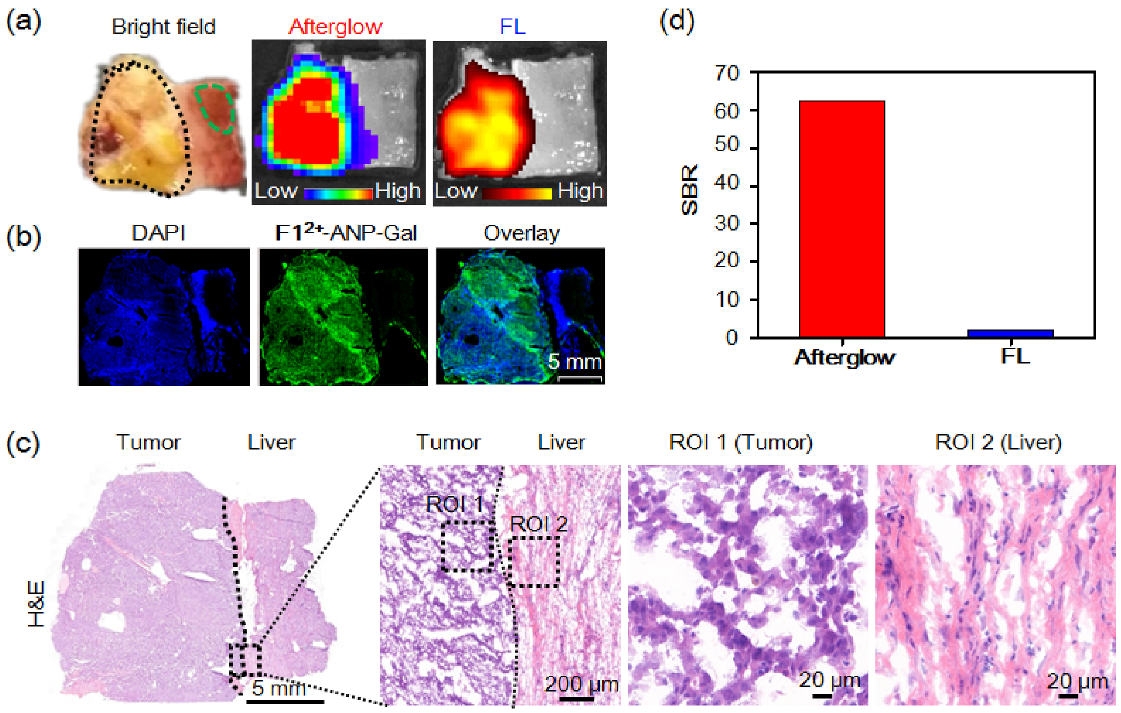

Supplementary Figure 39. Afterglow imaging of liver tumor tissue in excised HCC specimen. (**a**) Representative photograph (Bright field), afterglow and FL images of the liver specimen resected from an HCC patient. The specimen was incubated with **F1**$^{2+}$-ANP-Gal (58/28/2.2 μg mL^{-1} **F1**$^{2+}$(BF_4^-)$_2$/MEH-PPV/NIR775) in PBS buffer (1×, pH 7.4) at 37 ºC for 3 h, and then rinsed with PBS buffer for three times. The whole specimen was irradiated with the 808 nm laser (1 W cm^{-2}, 1 min). After cessation of laser, the afterglow image was acquired under an open filter, with acquisition time of 60 s. The fluorescence image was collected with $\lambda_{ex/em}$ = 740/790 nm. (**b**) Fluorescence images of liver tissue slices dissected from the HCC specimen after incubation with **F1**$^{2+}$-ANP-Gal (green) for 3 h and stained with DAPI (Blue). (**c**) H&E staining of the liver tissue slice dissected from the HCC specimen. The black dash boxes indicate the enlarged areas, in which the box labeled ROI 1 shows the tumor tissue and the box labeled ROI 2 indicates the normal liver tissue in the resected liver tissue slice, respectively. (**d**) SBR for afterglow and fluorescence imaging of liver specimen resected from the HCC patient. Black dash box in (**a**) indicates the location of tumor, and the green dash box indicates the location of normal liver tissue selected as the background. Source data are provided as a Source Data file.

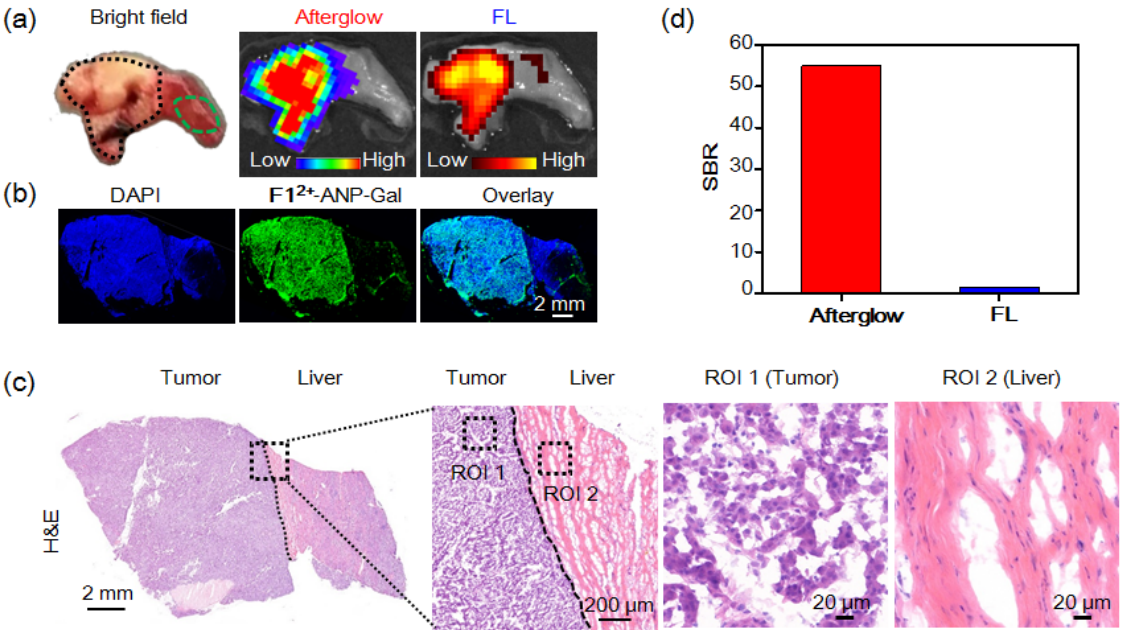

Supplementary Figure 40. Afterglow imaging of liver tumor tissue in excised HCC specimen. (**a**) Representative photograph (Bright field), afterglow and FL images of the liver specimen resected from an HCC patient. The specimen was incubated with **F1**$^{2+}$-ANP-Gal (58/28/2.2 μg mL^{-1} **F1**$^{2+}$(BF_4^-)$_2$/MEH-PPV/NIR775) in PBS buffer (1×, pH 7.4) at 37 ºC for 3 h, and then rinsed with PBS buffer for three times. The whole specimen was irradiated with the 808 nm laser (1 W cm^{-2}, 1 min). After cessation of laser, the afterglow image was acquired under an open filter, with acquisition time of 60 s. The fluorescence image was collected with $\lambda_{ex}/_{em}$ = 740/790 nm. (**b**) Fluorescence images of liver tissue slices dissected from the HCC specimen after incubation with **F1**$^{2+}$-ANP-Gal (green) for 3 h and stained with DAPI (Blue). (**c**) H&E staining of the liver tissue slice dissected from the HCC specimen. The black dash box indicate the enlarged areas, in which the box labeled ROI 1 shows the tumor tissue and the box labeled ROI 2 indicates the normal liver tissue in the resected liver tissue slice, respectively. (**d**) SBR for afterglow and fluorescence imaging of liver specimen resected from the HCC patient. Black dash box in (**a**) indicates the location of tumor, and the green dash box indicates the location of normal liver tissue selected as the background. Source data are provided as a Source Data file.

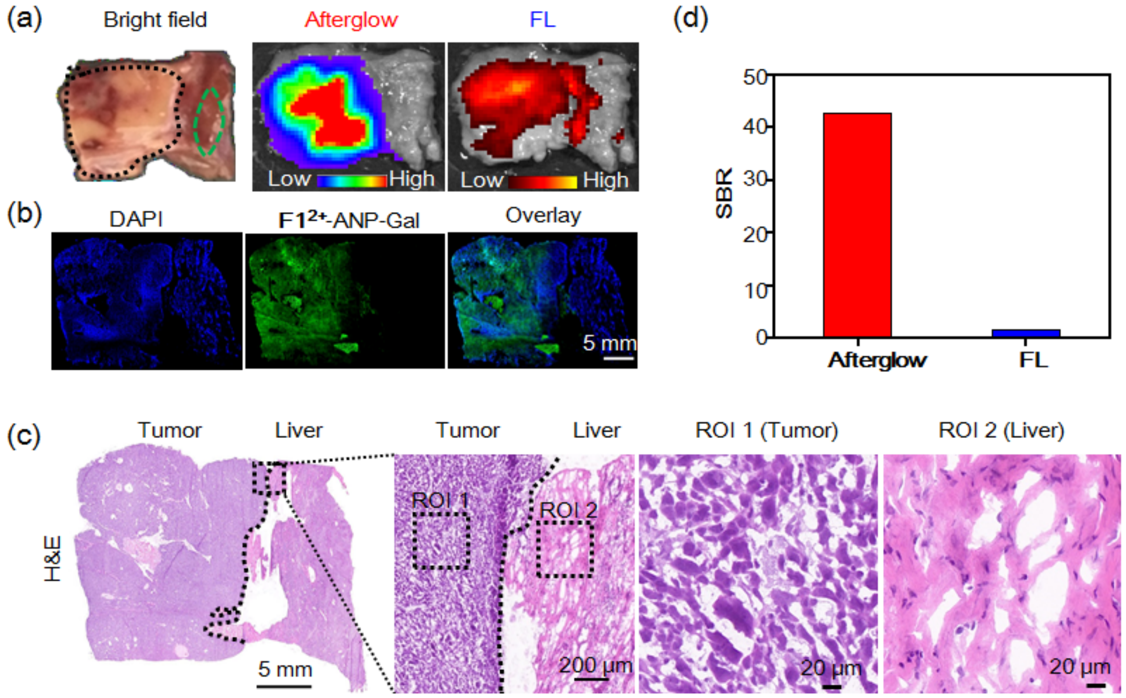

Supplementary Figure 41. Afterglow imaging of liver tumor tissue in excised HCC specimen. (**a**) Representative photograph (Bright field), afterglow and FL images of the liver specimen resected from an HCC patient. The specimen was incubated with **F1**$^{2+}$-ANP-Gal (58/28/2.2 μg mL^{-1} **F1**$^{2+}$(BF_4^-)$_2$/MEH-PPV/NIR775) in PBS buffer (1×, pH 7.4) at 37 ºC for 3 h, and then rinsed with PBS buffer for three times. The whole specimen was irradiated with the 808 nm laser (1 W cm^{-2}, 1 min). After cessation of laser, the afterglow image was acquired under an open filter, with acquisition time of 60 s. The fluorescence image was collected with $\lambda_{ex}/_{em}$ = 740/790 nm. (**b**) Fluorescence images of liver tissue slices dissected from the HCC specimen after incubation with **F1**$^{2+}$-ANP-Gal (green) for 3 h and stained with DAPI (Blue). (**c**) H&E staining of the liver tissue slice dissected from the HCC specimen. The black dash box indicate the enlarged areas, in which the box labeled ROI 1 shows the tumor tissue and the box labeled ROI 2 indicates the normal liver tissue in the resected liver tissue slice, respectively. (**d**) SBR for afterglow and fluorescence imaging of liver specimens resected from the HCC patient. Black dash box in (**a**) indicates the location of tumor, and the green dash box indicates the location of normal liver tissues selected as the background. Source data are provided as a Source Data file.

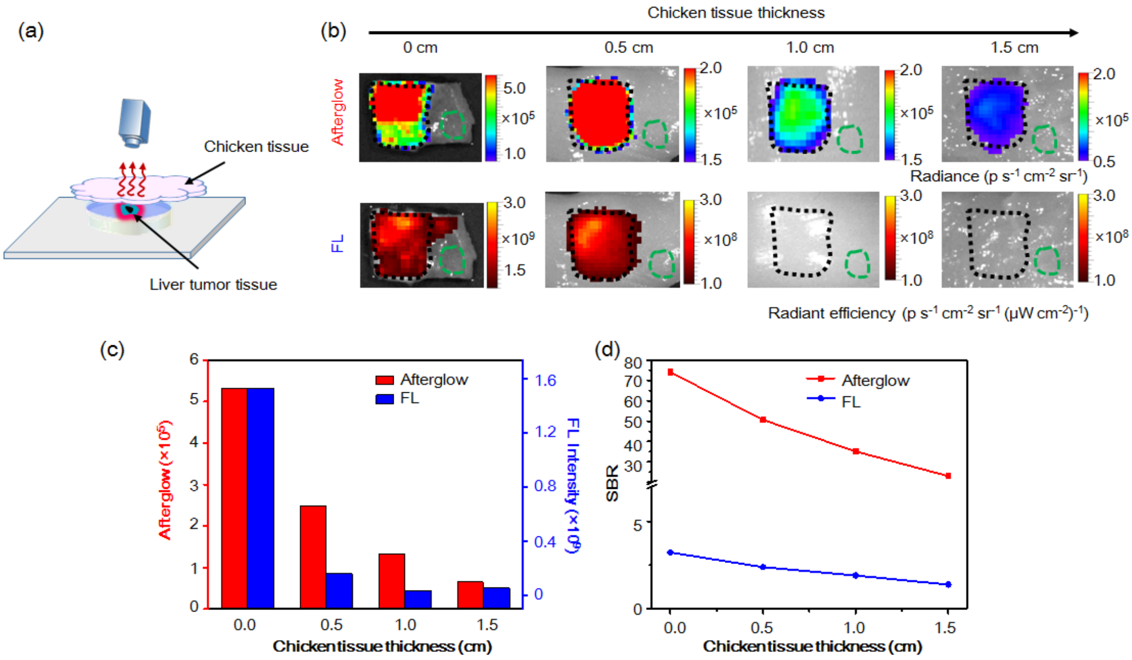

Supplementary Figure 42. Tissue penetration study of **F1**$^{2+}$-ANP-Gal in HCC specimen. (**a**) Schematic Illustration of the afterglow luminescence imaging of a **F1**$^{2+}$-ANP-Gal stained HCC specimen covered with chicken tissues at different thickness. (**b**) Afterglow (up) and fluorescence (down) images of **F1**$^{2+}$-ANP-Gal stained HCC specimens covered with chicken tissues at different thickness (0, 0.5, 1.0 and 1.5 cm). Clinically resected HCC specimen was infiltrated with **F1**$^{2+}$-ANP-Gal (58/28/2.2 μg mL^{-1} **F1**$^{2+}$(BF_4^-)$_2$/MEH-PPV/NIR775) in PBS buffer (pH 7.4) at 37 ºC for 3 h, and then rinsed with PBS buffer (pH 7.4) for three times. The whole specimen was irradiated with the 808 nm laser (1 W cm^{-2}, 1 min), and then covered with chicken tissues at different thickness. The afterglow images were acquired under an open filter, with acquisition time of 60 s. The fluorescence images were collected with $\lambda_{ex/em}$ = 740/790 nm. (**c**) The average afterglow (red) and fluorescence (blue) intensities and (**d**) SBRs of HCC specimen covered with chicken tissues at different thickness. Black circles indicate the locations of tumor tissues, and green circles indicate the locations of liver background. The results suggest that the enhanced penetration depth of NIR afterglow can allow **F1**$^{2+}$-ANP-Gal to detect liver tumor tissues in HCC specimens in deeper locations. Source data are provided as a Source Data file.

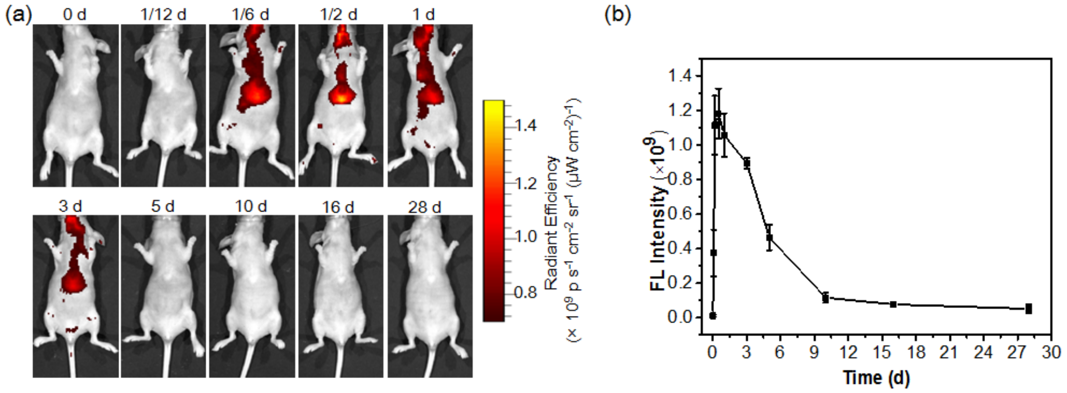

Supplementary Figure 43. The clearance of **F1**$^{2+}$-ANP-Gal in vivo. (**a**) Representative fluorescence images of nude mice at 0, 2 h, 4 h, 12 h, 1 d, 3 d, 5 d, 10 d, 16 d and 28 d after systemic administration of **F1**$^{2+}$-ANP-Gal (211/100/8 μg **F1**$^{2+}$(BF_4^-)$_2$/MEH-PPV/NIR775, in 200 μL saline) via tail vein injection. The fluorescence images were acquired at 790 nm upon excitation at 740 nm. (**b**) Quantification of NIR fluorescence of liver in living mice as a function of time. Values are mean ± s.d. (n = 3). Source data are provided as a Source Data file.

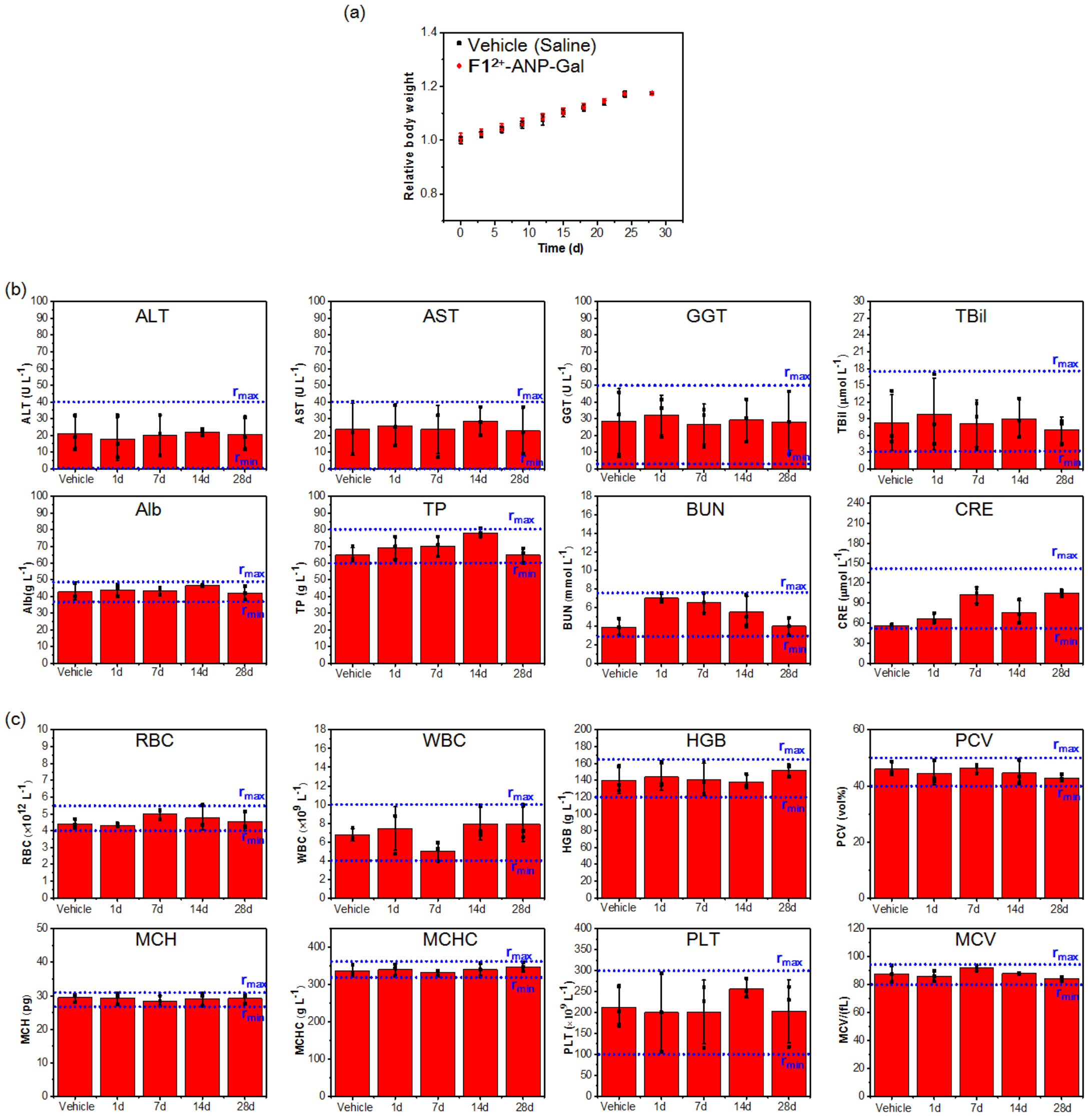

Supplementary Figure 44. Investigation of the biosafety of **F1**$^{2+}$-ANP-Gal in healthy mice. (**a**) Plot of the normalized body weight of mice after tail vein injection of **F1**$^{2+}$-ANP-Gal (211/100/8 μg **F1**$^{2+}$(BF_4^-)$_2$/MEH-PPV/NIR775, in 200 μL saline) or saline (Vehicle) for over 28 days. (**b**) Biochemistry and (**c**) blood count analysis of mice after i.v. injection of saline or **F1**$^{2+}$-ANP-Gal at 1, 7, 14 and 28 d. Abbreviations: ALT, alanine aminotransferase; AST, glutamic oxaloacetic transaminase; GGT, γ-glutamyl transpeptidase; TBil, total bilirubin; Alb, albumin; TP, total protein; BUN, blood urea nitrogen; CRE, creatinine; RBC, red blood cells; WBC, white blood cells; HGB, hemoglobin; HCT, hematocrit; MCH, mean corpuscular hemoglobin; MCHC, mean corpuscular hemoglobin concentration; PLT, platelet count; MCV, mean corpuscular volume. Blue short dotted lines indicate the range of normal reference values ($r_{min} \rightarrow r_{max}$) for BALB/c mice. Values are mean ± s.d. (n = 3). Source data are provided as a Source Data file.

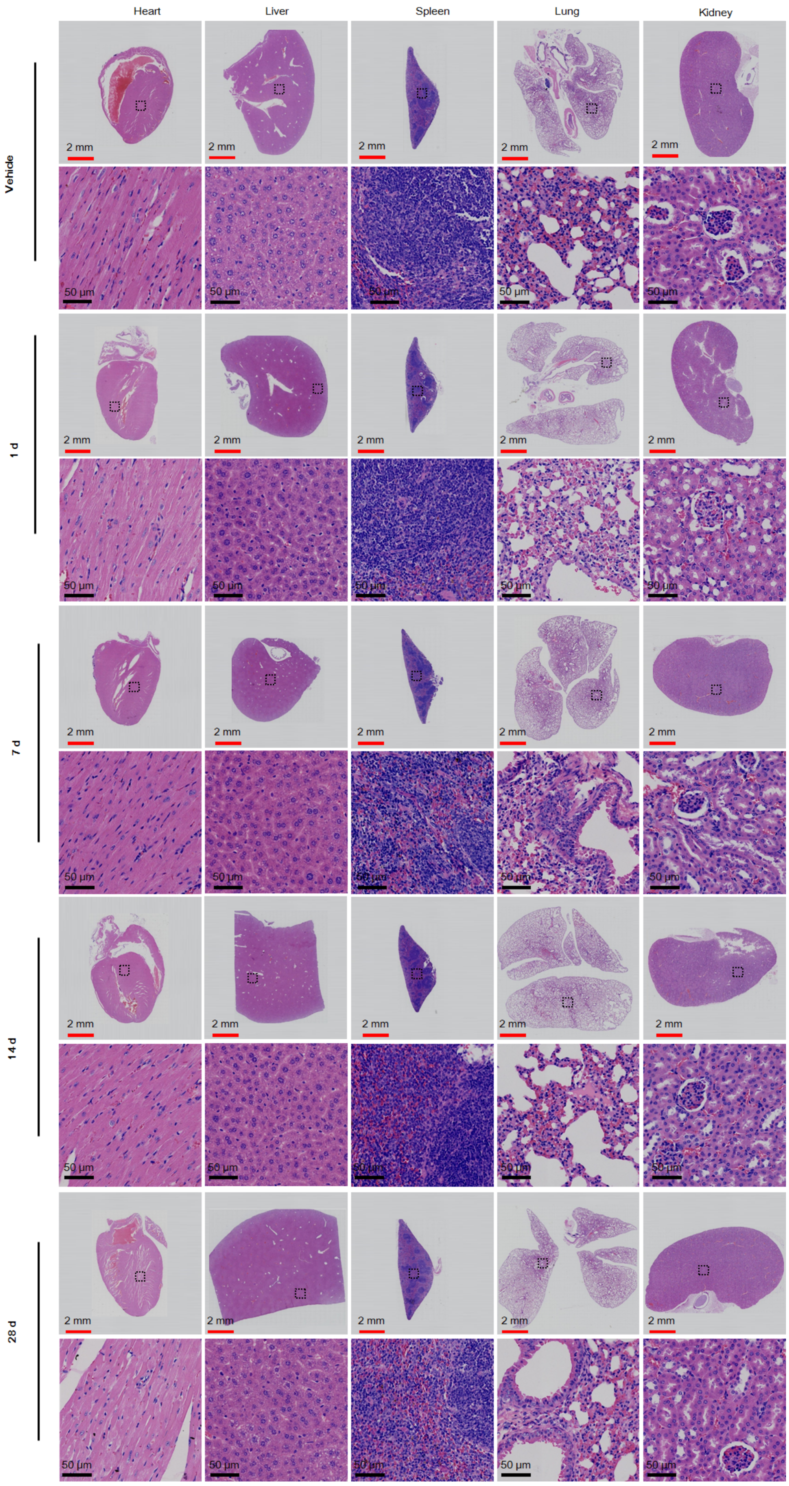

Supplementary Figure 45. Histologic analysis of major organs resected from mice. Representative H&E staining images of the main organs resected from the mice after i.v. injection of saline or **F1**$^{2+}$-ANP-Gal (211/100/8 μg **F1**$^{2+}$(BF_4^-)$_2$/MEH-PPV/NIR775, in 200 μL saline) on indicated days (1, 7, 14 and 28 days). The dot boxes indicated the enlarged areas in the intact tissue slices.

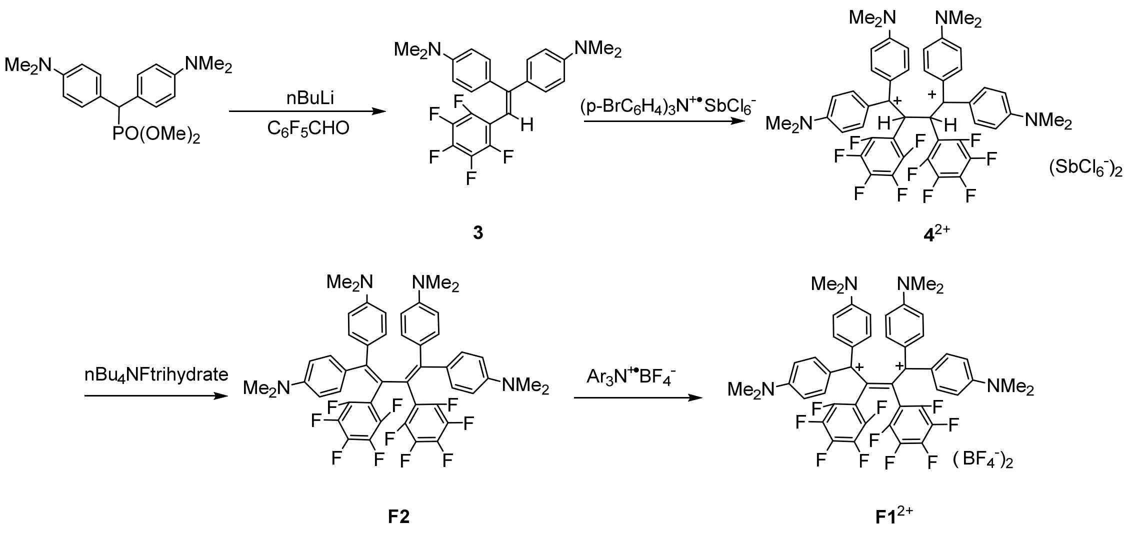

Supplementary Figure 46. Synthesis of **F1**$^{2+}$(BF_4^-)$_2$.

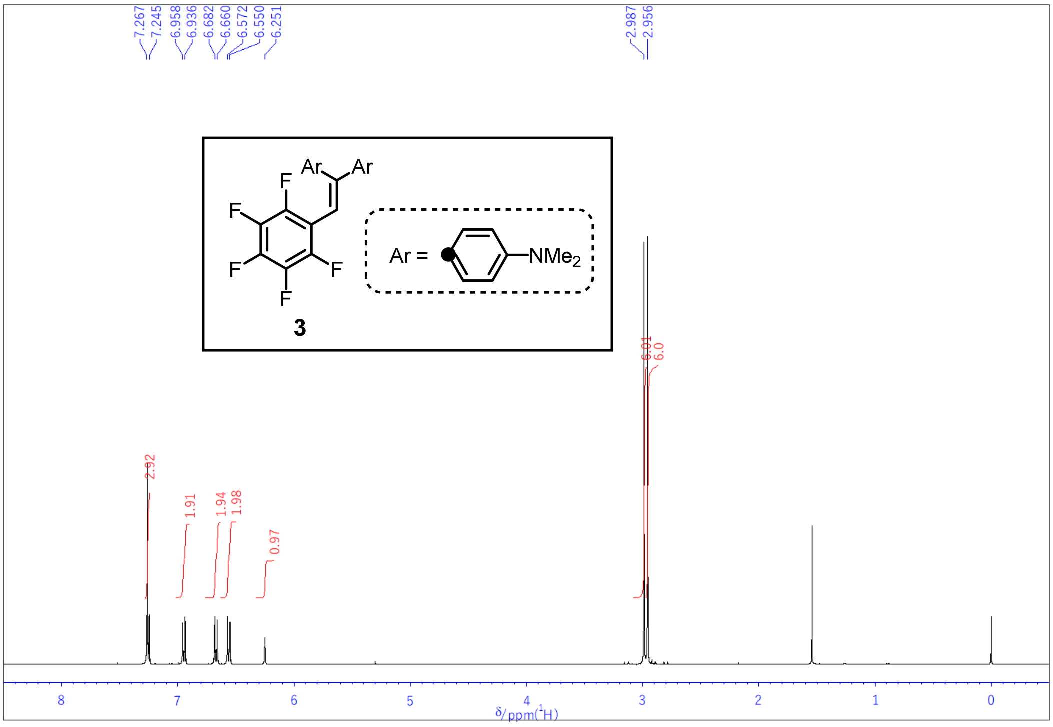

Supplementary Figure 47. ^{1}H NMR spectra of **3** in $CDCl_3$.

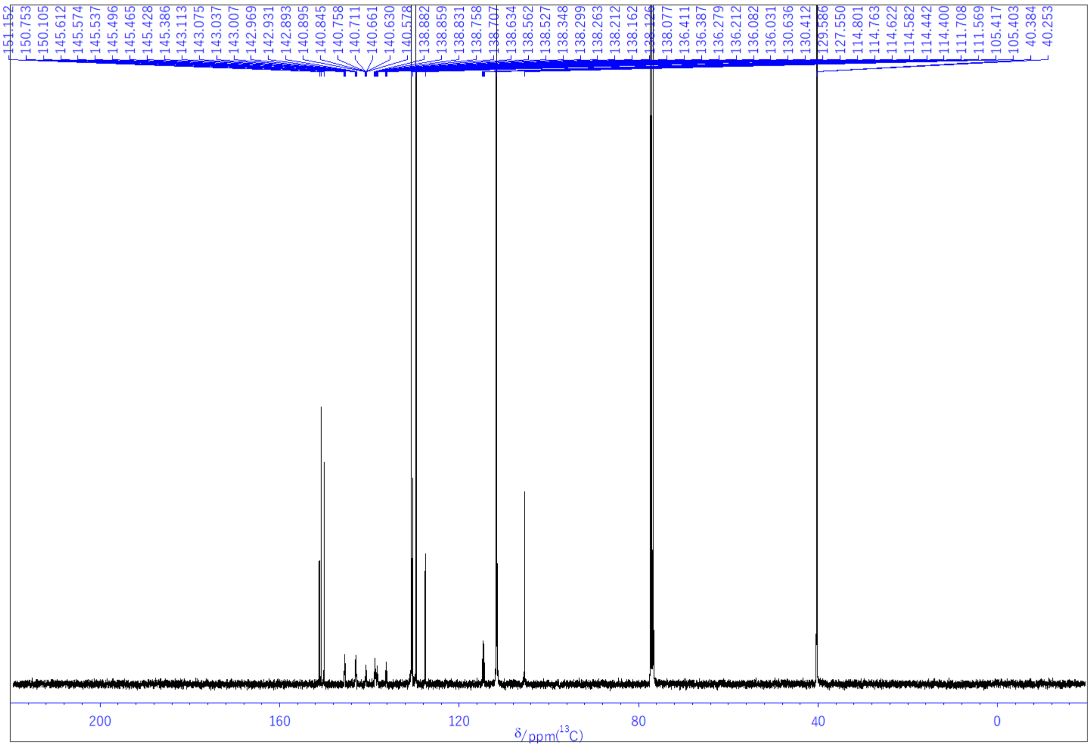

Supplementary Figure 48. ^{13}C NMR spectra of **3** in $CDCl_3$.

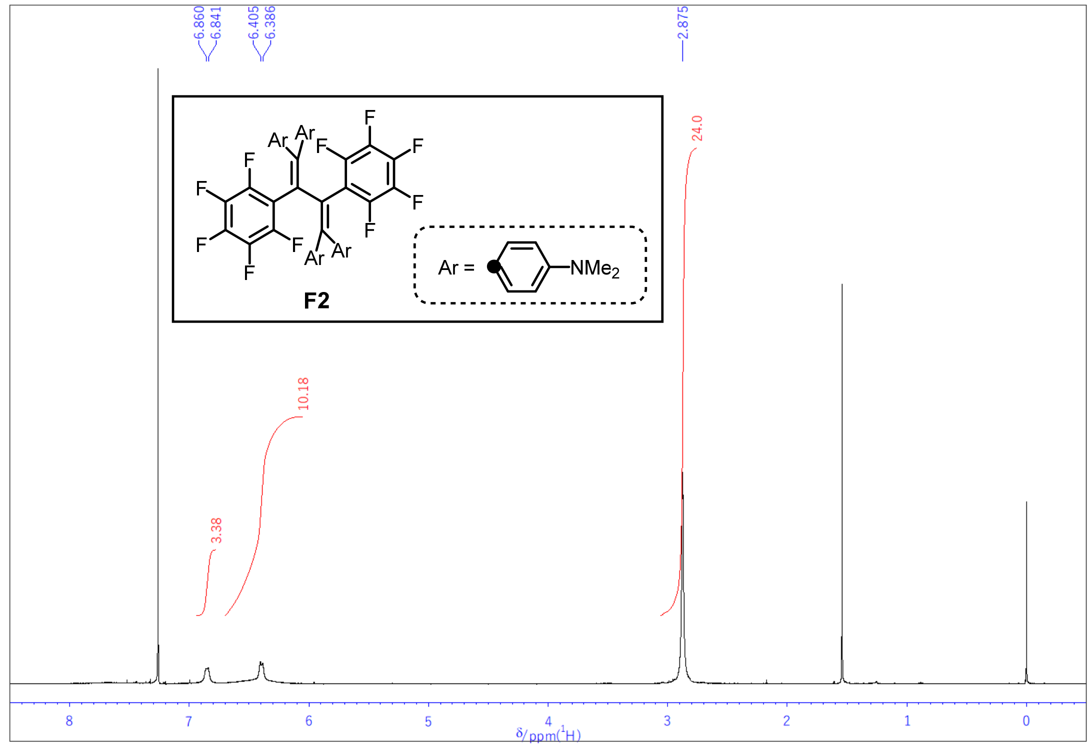

Supplementary Figure 49. ^{1}H NMR spectra of **F2** in $CDCl_3$.

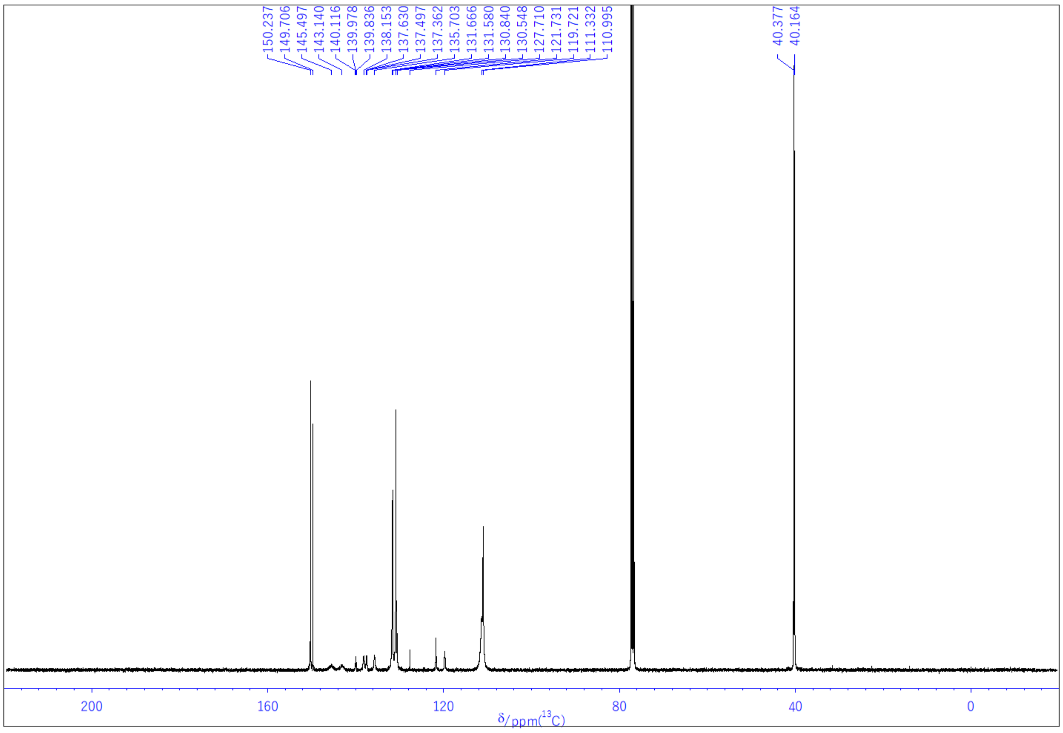

Supplementary Figure 50. ^{13}C NMR spectra of **F2** in $CDCl_3$.

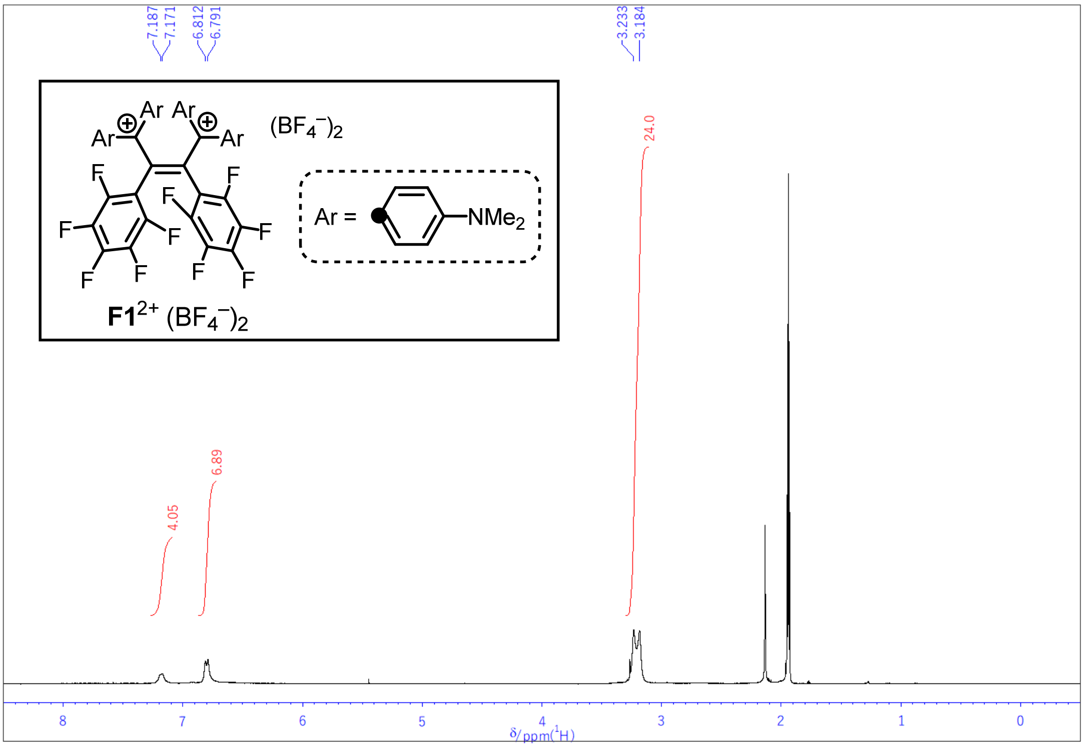

Supplementary Figure 51. ^{1}H NMR spectra of **F1**$^{2+}$ ·$(BF_4^-)_2$ in CD_3CN.

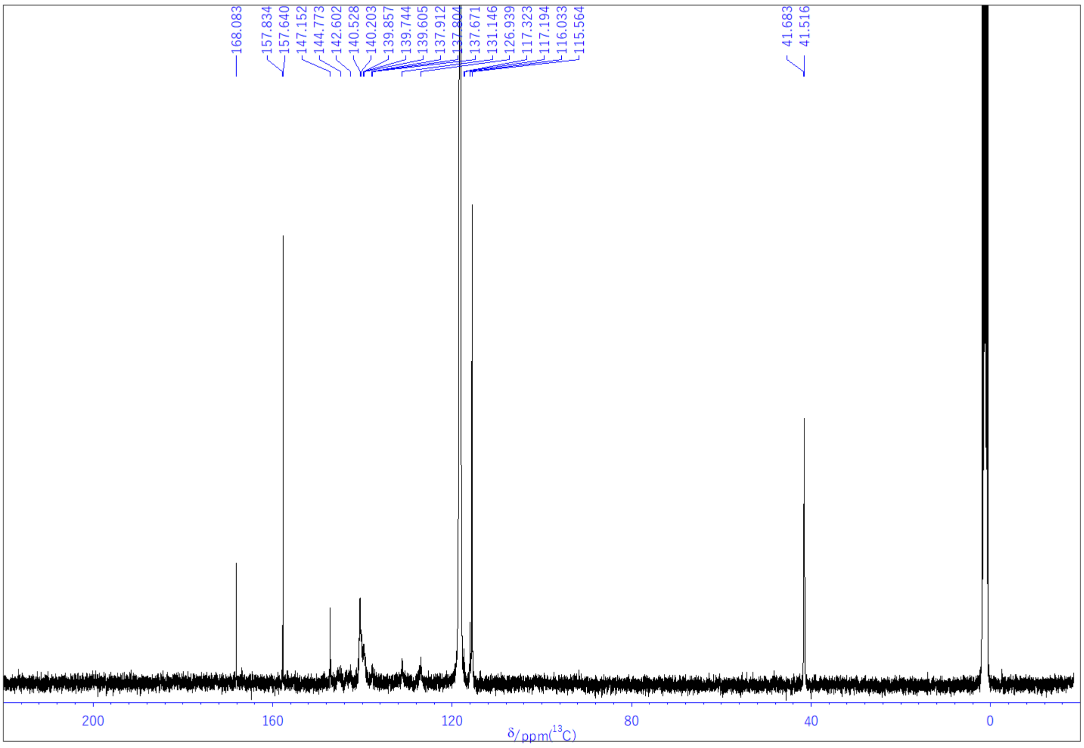

Supplementary Figure 52. ^{13}C NMR spectra of **F1**$^{2+}$·$(BF_4^-)_2$ in CD_3CN.

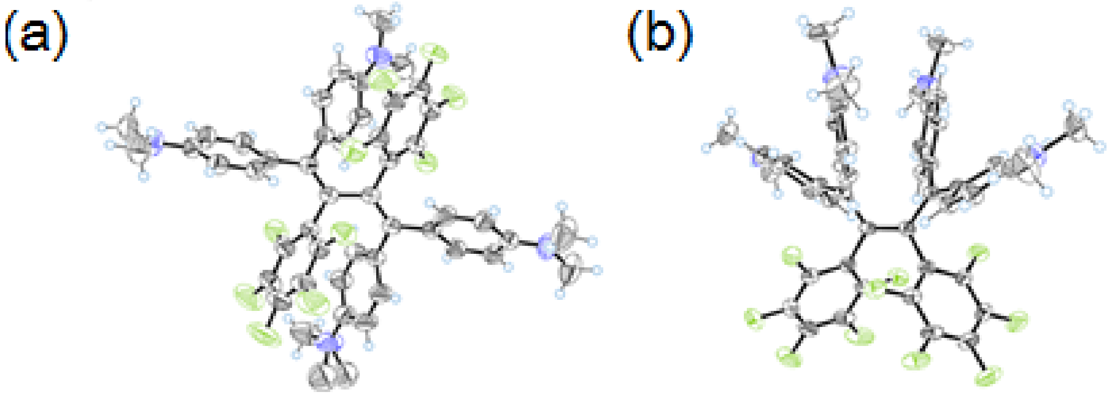

Supplementary Figure 53. ORTEP drawings. (**a**) The ORTEP drawing of **F2**·0.25(MeOH) at 150 K. (**b**) The ORTEP drawing of **F1**$^{2+}$·$(BF_4^-)_2$·MeCN at 150 K. Solvent molecules and counterions are omitted for clarity. Thermal ellipsoids are shown at the 50% probability level.

Supplementary Methods

Materials. All chemicals were purchased from Sigma-Aldrich unless otherwise stated and used without further purification. 1,2-Distearoyl-sn-glycero-3-phosphoethanolamine-N-[amino(polyethylene glycol)-2000] (ammonium salt) ($DSPE-PEG_{2000}-NH_2$) and 1,2-distearoyl-sn-glycero-3-phosphoethanolamine-N-[methoxy (polyethylene glycol)-2000] ($DSPE-PEG_{2000}$) were purchased from Avanti (Alabaster, AL, USA). (Poly[2-methoxy-5-92-ethylhexyloxy)-1,4-penylene-vinylene] (MEH-PPV) was obtained from Xi'an Polymer Light Technology Corp. Singlet oxygen sensor green (SOSG) was obtained from Invitrogen (Carlsbad, CA, USA). 3-(4,5-dimethylthiazol-2-yl)-2,5-diphenyltetrazolium bromide (MTT) kit were obtained from KeyGen Biotech. Co. Ltd. (Nanjing, China).

Preparation of 1,1-bis[4-(dimethylamino)phenyl]-2-(2,3,4,5,6-pentafluorophenyl)ethene (3). To a solution of dimethyl bis(4-dimethylaminophenyl)methyl phosphonate (2.00 g, 5.52 mmol) in dry THF (80 mL) was added *n*BuLi (1.60 M solution in hexane, 3.60 mL, 5.76 mmol) dropwise over 5 min at -78 °C, and the mixture was stirred for 2 h. To the solution was added pentafluorobenzaldehyde (1.92 g, 9.81 mmol) at -78 °C. The mixture was stirred for 2 h at -78 °C, gradually warmed up to 24 °C, and stirred for 18 h. After being diluted with water, the whole mixture was extracted with CH_2Cl_2 three times. The combined organic layers were washed with water and brine, and dried over anhydrous Na_2SO_4. After filtration, the solvent was concentrated under reduced pressure. The residue was purified by column chromatography on silica gel (hexane/EtOAc = 20) to give **3** (1.70 g) as a yellow solid in 93% yield.

3: Mp 162-165 °C; ^{1}H NMR ($CDCl_3$): δ/ppm 7.26 (2H, d, *J*= 8.8 Hz), 6.95 (2H, d, *J*= 8.8 Hz), 6.67 (2H, d, *J*= 8.8 Hz), 6.56 (2H, d, *J*= 8.8 Hz), 6.25 (1H, s), 2.99 (6H, s), 2.96 (6H, s) (Supplementary Fig. 47); ^{13}C NMR ($CDCl_3$): δ/ppm 151.15, 150.75, 150.11, 144.25 (C, dddd, $J_{C\text{-}F}$ = 3.9 Hz, 7.5 Hz, 12 Hz, 251 Hz), 139.49 (C, dddd, $J_{C\text{-}F}$ = 4.8 Hz, 8.8 Hz, 14 Hz, 252 Hz), 137.46 (C, dddd, $J_{C\text{-}F}$ = 5.1 Hz, 12 Hz, 25 Hz, 249 Hz), 130.64, 130.41, 129.59, 127.55, 114.60 (C, dt, $J_{C\text{-}F}$ = 4.0 Hz, 18 Hz), 111.64 (C, d, $J_{C\text{-}F}$ = 14 Hz), 105.42, 105.40, 40.38, 40.25 (Supplementary Fig. 48); IR (KBr): ν/cm^{-1} 3039, 2890, 2856, 2806, 1610, 1593, 1547, 1519, 1490, 1444, 1421, 1360, 1306, 1229, 1208, 1195, 1168, 1156, 1121, 1063, 1012, 998, 973, 949, 839, 818; LR-MS(FD) m/z (%): 434.18 (4), 433.18 (28), 432.17 (M^+, bp); Anal. Calcd. (%) for

$C_{24}H_{21}F_5N_2$: C 66.66, H 4.89, N 6.48; found: C 66.49, H 4.86, N 6.40; UV-vis (CH_2Cl_2): λ_{max}/nm (ε/$M^{-1}cm^{-1}$) 329 (26300), 262 (17600), 242 (19500).

Preparation of 1,1,4,4-tetrakis[4-(dimethylamino)phenyl]-2,3-bis(2,3,4,5,6-pentafluorophenyl)buta-1,3-diene (F2). To a solution of **3** (988 mg, 2.28 mmol) in dry CH_2Cl_2 (30 mL) was added tris(4-bromophenyl)aminium hexachloroantimonate (1.86 mg, 2.28 mmol) at 0 °C, and the mixture was stirred for 1 h. The solvent was concentrated under reduced pressure to give $\mathbf{4}^{2+}$·$(SbCl_6^{-})_2$ (1.75 g) as a dark blue powder. The crude product was used in the next reaction without further purification.

To a solution of $\mathbf{4}^{2+}$·$(SbCl_6^{-})_2$ (1.75 g) in MeCN (15 mL) was added a solution of Bu_4NF trihydrate (4.66 g, 14.8 mmol) in MeCN (10 mL) at -20 °C. After the solution was stirred at -20 °C for 30 min, activated Zn powder (746 mg, 11.4 mmol) was added to the purple solution. The reaction mixture was stirred at -20 °C for an additional 30 min. After the addition of saturated $NaHCO_3$ aq., the whole mixture was extracted with ether five times, and dried over anhydrous Na_2SO_4. After filtration, the solvent was concentrated under reduced pressure. The crude product was purified by column chromatography on aluminum oxide (hexane/EtOAc = 20) to give **F2** (404 mg) as a yellow solid in 41% yield.

F2: Mp 249-251 °C; ^{1}H NMR ($CDCl_3$): δ/ppm 6.85 (4H, br-d, *J*= 7.4 Hz), 6.70-6.10 (8H, br), 6.40 (4H, br-d, *J*= 7.6 Hz), 2.88 (24H, br-s) (Supplementary Fig. 49); ^{13}C NMR ($CDCl_3$): δ/ppm 150.24, 149.71, 144.54 (C, d, $J_{C\text{-}F}$ = 242 Hz), 138.74 (C, td, $J_{C\text{-}F}$ = 14 Hz, 250 Hz), 136.93 (C, d, $J_{C\text{-}F}$ = 247 Hz), 131.67, 131.58, 130.84, 130.55, 127.71, 121.73, 119.72, 111.33, 111.00, 40.38, 40.16 (Supplementary Fig.50); IR (KBr): ν/cm^{-1} 3036, 2917, 2850, 2803, 1606, 1558, 1519, 1487, 1444, 1356, 1304, 1226, 1204, 1190, 1163, 1124, 1060, 1017, 999, 987, 947, 826, 820, 808, 790; LR-MS(FD) m/z (%): 864.34 (17), 863.34 (59), 862.34 (M^+, bp); HR-MS(FD) Calcd. For $C_{48}H_{40}F_{10}N_4$: 862.3093. Found: 862.3099; UV-vis (CH_2Cl_2): λ_{max}/nm (ε/$M^{-1}cm^{-1}$) 428 (35200), 360 (28200), 294 (31700), 233 (34800).

Preparation of (*Z*)-1,1,4,4-tetrakis[4-(dimethylamino)phenyl]-2,3-bis(2,3,4,5,6-pentafluorophenyl)but-2-ene-1,4-diylium bis(tetrafluoroborate) [$\mathbf{F1}^{2+}$·$(BF_4^{-})_2$]. To a solution of **F2** (99.9 mg, 116 μmol) in dry CH_2Cl_2 (2 mL) was added tris(4-

bromophenyl)aminium tetrafluoroborate (131.9 mg, 232 μmol) at 0 °C, and the mixture was stirred at 25 °C for 1 h. The addition of dry ether led to precipitation of the dication salt. The precipitates were collected, washed with dry ether three times, and dried *in vacuo* to give **F1**$^{2+}$·$(BF_4^-)_2$ (113.5 mg) as a dark green powder in 95% yield.

F1^{2+}·$(BF_4^-)_2$: Mp 220-226 °C (decomp.); ^{1}H NMR (CD_3CN): δ/ppm 7.18 (8H, brd, J= 6.6 Hz), 6.80 (8H, brd, J= 8.7 Hz), 3.23 (12H, s), 3.18 (12H, s) (Supplementary Fig. 51); ^{13}C NMR (CD_3CN): δ/ppm 168.08, 157.83, 157.64, 147.15, 143.69 (C, d, $J_{C\text{-}F}$ = 219 Hz), 140.53, 140.20, 138.77 (C, td, $J_{C\text{-}F}$ = 12 Hz, 196 Hz), 131.15, 126.94, 117.32, 117.19, 116.03, 115.56, 41.68, 41.52 (Supplementary Fig. 52); IR (KBr): ν/cm^{-1} 3081, 2927, 2866, 2817, 1650, 1612, 1583, 1522, 1495, 1371, 1226, 1173, 1124, 1084, 1063, 990, 938, 906, 834, 788, 759, 736, 725, 643, 565, 522, 511, 484, 454, 412; LR-MS(FD) m/z (%): 864.25 (16), 863.24 (55), 862.24 ($[M^{2+}+e^-]^+$, bp), 431.11 (M^{2+}, 3); HR-MS(FD) Calcd. For $C_{48}H_{40}F_{10}N_4$ $[M^{2+}+e^-]^+$: 862.3114. Found: 862.3093; UV-vis (CH_2Cl_2): λ_{max}/nm (ε/M^{-1}cm^{-1}) 769 (34800), 558 (69700), 420 (11500), 382 (14300), 313 (24100), 244 (21300).

Method of crystal data. Data collection was conducted with a Rigaku Mercury 70 diffractometer (Mo-K radiation, = 0.71075 Å). The structure was solved by the direct method (SIR2004) and refined by the full-matrix least-squares method on F^2 with anisotropic temperature factors for non-hydrogen atoms. All the hydrogen atoms were located at the calculated positions and refined with riding.

Crystal data of F2·0.25(MeOH)**.** Crystals were obtained by recrystallization from THF/MeOH. MF: $C_{48.25}H_{41}F_{10}N_4O_{0.25}$, FW: 870.87, red block, 0.30 × 0.30 × 0.20 mm^3, monoclinic $P2_1/c$, a = 25.486(6) Å, b = 12.595(3) Å, c = 14.455(3) Å, β = 103.821(3) °, V = 4506(2) Å^3, ρ (Z = 4) = 1.284 g cm^{-1}, A total of 19021 reflections ($2\theta_{max}$ = 55°) were measured at T = 230 K. Numerical absorption correction was applied (μ = 1.048 cm^{-1}). The final $R1$ and $wR2$ values are 0.0694 ($I > 2\sigma I$) and 0.2232 (all data) for 7842 reflections and 570 parameters. Estimated standard deviations are 0.003-0.010 Å for bond lengths and 0.19-0.6 ° for bond angles (Supplementary Fig. 53a). CCDC 1909735

Crystal data of F1^{2+}·$(BF_4^-)_2$·MeCN**.** Crystals were obtained by recrystallization from MeCN/ether. MF: $C_{50}H_{43}B_2F_{18}N_5$, FW: 1077.51, purple rod, 0.50 × 0.20 × 0.20 mm^3, orthorhombic $Pca2_1$, a = 14.913(3) Å, b = 15.558(3) Å, c = 21.037(4) Å, V = 4881(2) Å^3, ρ (Z

= 4) = 1.466 g cm^{-1}, A total of 35898 reflections ($2\theta_{max}$ = 55°) were measured at T = 150 K. Numerical absorption correction was applied (μ = 1.343 cm^{-1}). The final $R1$ and $wR2$ values are 0.0540 ($I > 2\sigma I$) and 0.1512 (all data) for 8516 reflections and 704 parameters. Estimated standard deviations are 0.004-0.014 Å for bond lengths and 0.3-1.3 ° for bond angles (Supplementary Fig. 53b). CCDC 1909737

Fluorescence lifetime measurements. The fluorescence lifetime of the NIR775 was measured on a FLS 920 spectrophotometer (Edinburgh instruments, U.K.) with excitation wavelength at 740 nm from super-continuum pulsed laser source. The instrument is based on time-correlated single photon counting (TCSPC). Photons are counted in a time window which sweeps across the full time range following each excitation pulse, creating a histogram of counts versus time. The data quality of the resulting histogram is improved by adding the data of repeated sweeps.

The fluorescence lifetime of the MEH-PPV was measured on a FL-3 spectrophotometer (HORIBA) based on time-correlated single photon counting (TCSPC). The excitation source was a picosecond pulsed diode laser with excitation wavelength at 405 nm.

Investigation of the reaction between $\mathbf{F1^{2+}(BF_4^-)_2}$ and $\mathbf{H_2S}$. To investigate the reaction between **F1**$^{2+}(BF_4^-)_2$ and H_2S, **F1**$^{2+}(BF_4^-)_2$ was dissolved in DMSO to prepare the stock solution (10 mM), which was then diluted with PBS buffer (1.0 mL, 5% DMSO) to prepare 9 μg mL^{-1} **F1**$^{2+}(BF_4^-)_2$ for the measurement. Briefly, **F1**$^{2+}(BF_4^-)_2$ (9 μg mL^{-1}) in 1.0 mL PBS buffer (pH 7.4, 5% DMSO) was incubated with 90 μM NaHS at 37 ºC for 1 min. The reaction was then analyzed by HPLC and the UV-vis absorption spectra before and after incubation with NaHS was acquired.

Measurement of the reaction kinetics between $\mathbf{F1^{2+}(BF_4^-)_2}$ and $\mathbf{H_2S}$. To measure the reaction kinetics between **F1**$^{2+}(BF_4^-)_2$ and H_2S, **F1**$^{2+}(BF_4^-)_2$ (9 μg mL^{-1} in 0.2 mL PBS buffer containing 5% DMSO, pH 7.4) in a 96-well transparent plate was incubated with varying concentrations (0, 40, 50, 60 and 90 μM) of NaHS at r.t.. The UV-vis absorbance of each reaction solution at 550 nm was monitored every 5 s on the Tcan microplate reader, and last for 270 s. The UV-vis absorbance at each time point was normalized to that of **F1**$^{2+}(BF_4^-)_2$ (9 μg mL^{-1}) alone. The pseudo-first-order rate (k_{obs}) of **F1**$^{2+}(BF_4^-)_2$ incubated with indicated concentration of NaHS was

determined by fitting the normalized absorption intensity with single exponential function of:

$y = y_0 + A \times \exp(R_0 \times t)$, where $k_{\mathrm{obs}} = -R_0$.

Therefore, plot of k_{obs} versus NaHS concentration (40-90 μM) affords the second-order reaction rate, k_2.

Measurement of the reaction kinetics between F1^{2+}-ANP and H_2S. To measure the reaction kinetics between **F1**$^{2+}$-ANP and H_2S, **F1**$^{2+}$-ANP (9/4.5/0.35 μg mL^{-1} **F1**$^{2+}$$(BF_4^-)_2$/MEH-PPV/NIR775 in 0.2 mL PBS buffer, pH 7.4) in a 96-well transparent plate was incubated with varying concentrations (0, 40, 50, 60 and 90 μM) of NaHS at r.t.. The fluorescence intensity of each reaction solution at 780 nm (λ_{ex} = 740 nm) was monitored every 5 s on the Tecan microplate reader, and last for 270 s. The fluorescence intensity at each time point was normalized to the maximum fluorescence intensity achieved. The pseudo-first-order rate (k_{obs}) of **F1**$^{2+}$-ANP incubated with indicated concentration of NaHS was determined by fitting the normalized absorption intensity with single exponential function of:

$y = y_0 + A \times \exp(R_0 \times t)$, where $k_{\mathrm{obs}} = -R_0$.

Therefore, plot of k_{obs} versus NaHS concentration (40-90 μM) affords the value of second-order reaction rate, k_2.

Measurement of the sensitivity of F1^{2+}-ANP for H_2S. To test the sensitivity of **F1**$^{2+}$-ANP for H_2S, **F1**$^{2+}$-ANP (2.2 μg NIR775, 28 μg MEH-PPV, 58 μg **F1**$^{2+}$$(BF_4^-)_2$) was incubated with varying concentrations of NaHS (0, 1.5, 3, 5, 10, 15, 20, 25, 30, 40, 50, 80, 125, 200, 250 μM) in 1.0 mL PBS buffer (1×, pH 7.4) at 37 °C for 1 min. The incubation solutions were then irradiated with the 808 nm laser (1 W cm^{-2}, 1 min). After removal of the laser, the afterglow luminescence images were immediately acquired on an IVIS Spectrum imaging system equipped with a 790 nm emission filter, with an acquisition time of 60 s. The afterglow luminescence intensity (I) in each image was quantified by applying ROI over the image, using

the Living Image Software (4.5.2, PerkinElmer, MA, U.S.A), and divided by the intensity of **F1**$^{2+}$-ANP in the absence of NaHS (I_0). Plot of I/I_0 versus the concentration of NaHS afford a linear regression between 0–50 μM NaHS. The detection limit were calculated by using the 3δ/ *k* method. The standard deviations (δ) and slope (*k*) were obtained from the blank measurements of 11 times and the linear plot.

Investigation of the selectivity of F1^{2+}-ANP toward H_2S. To examine the specificity of **F1**$^{2+}$-ANP toward H_2S, **F1**$^{2+}$-ANP (2.2 μg NIR775, 28 μg MEH-PPV, 58 μg **F1**$^{2+}$(BF_4^-)$_2$) in 1 mL PBS (pH 7.4) were treated with different biologically relevant reductant or ROS (200 μM NaHS, 1.25 mM L-Cys,10 mM GSH, 1 mM Hcy, 1.25 mM VC, 1.25 mM DTT, 100 μM BME, 1 mM H_2O_2, 1 mM ClO^-; $ONOO^-$ (1 mM $NaNO_2$+1 mM H_2O_2), $O_2^{\cdot-}$ (100 μM xanthine + 22 mU XO)) at 37 ºC for 10 min. The fluorescence spectra from 500 to 900 nm with excitation from 400 to 800 nm were recorded by fluorescence synchronous scanning, with offset of 100 nm. The fluorescence images were acquired on an IVIS Spectrum imaging system with $\lambda_{ex}/\lambda_{em}$ = 740/790 nm. For afterglow imaging, the incubation solutions were irradiated by the 808 nm laser (1 W cm^{-2}, 1 min). After removal of the laser, the afterglow luminescence images were immediately acquired on an IVIS Spectrum imaging system equipped with a 790 nm emission filter, with an acquisition time of 60 s. The afterglow luminescence intensities at 790 nm for **F1**$^{2+}$-ANP were quantified by applying ROI over the image, using the Living Image Software (4.5.2, PerkinElmer, MA, U.S.A).

Investigation of the effect of pH on the activation of F1^{2+}-ANP by H_2S. To examine the effect of pH on H_2S-mediated activation of **F1**$^{2+}$-ANP, **F1**$^{2+}$-ANP (2.2 μg NIR775, 28 μg MEH-PPV, 58 μg **F1**$^{2+}$(BF_4^-)$_2$) in PBS buffer (1.0 mL) under different pH values (5.0, 6.0, 7.4 and 8.0) were incubated with 200 μM NaHS at 37 ºC for 1 min. The fluorescence spectra were recorded from with excitation from using excitation from 400 to 800 nm and emission of 500 to 900 nm by fluorescence synchronous scanning mode on a HORIBA Jobin Yvon Fluoromax-4 fluorometer, with offset of 100 nm. For afterglow imaging, the incubation solutions with different pH values were illuminated with the 808 nm laser (1 W cm^{-2}, 1 min). After removal of the laser, the afterglow luminescence images were immediately acquired on an IVIS Spectrum imaging system equipped with an open filter, with an acquisition time of 60 s. The

afterglow luminescence intensities for **F1**$^{2+}$-ANP were quantified by applying ROI over the image, using the Living Image Software (4.5.2, PerkinElmer, MA, U.S.A).

Measurement of the H_2S Concentration in Mouse Blood. The concentration of H_2S in mouse blood was measured by an internal standard method. Brieftly, freshly collected mouse whole blood was diluted with PBS buffer (1×, pH 7.4) at 1:1 ratio. **F1**$^{2+}$-ANP (2.2/28/58 μg mL^{-1} NIR775/MEH-PPV/**F1**$^{2+}$(BF_4^-)$_2$) was then added into the two-fold diluted mouse blood (200 μL) containing 1 μL of $ZnCl_2$ (200 mM, H_2S scavenger) (0 μM HS^-), D.I. H_2O (x μM HS^-), or varying concentrations of NaHS (1 mM: x + 5 μM HS^-, 2 mM: x + 10 μM HS^-, 3 mM: x + 15 μM HS^-, 4 mM: x + 20 μM HS^-). The solutions were incubated at 37 ºC for 1 min, and then irradiated under the 808 nm laser (1 W cm^{-2}) for another 1 min. After removal of the laser, the afterglow luminescence images were immediately acquired on an IVIS Spectrum imaging system equipped with a 790 nm emission filter, with an acquisition time of 60 s. The afterglow luminescence intensities at 790 nm for **F1**$^{2+}$-ANP were quantified by applying ROI over the image, using the Living Image Software (4.5.2, PerkinElmer, MA, U.S.A). Linear plot of the afterglow intensity against the concetnration of added NaHS (5, 10, 15, 20 μM) affords the slope, which could be used to calculate the concentration of H_2S (x μM HS^-) in the two-fold diluted mouse blood with the quantified afterglow intensities in the solution. Therefore, the H_2S concentration in the mouse whole blood could be further calcualted.

Detection of $^1O_2$ Generation *in vitro*. The $^1O_2$ production capacity of **F1**$^{2+}$-ANP under 808 nm laser irradiation *in vitro* was evaluated using SOSG as a fluorescent $^1O_2$ indicator. Briefly, **F1**$^{2+}$-ANP (2.2/28/58 μg mL^{-1} NIR775/MEH-PPV/**F1**$^{2+}$(BF_4^-)$_2$) in PBS buffer was incubated with or without NaHS (200 μM) at 37 ºC for 1 min. Then, the **F1**$^{2+}$-ANP solution was mixed with SOSG (20 μM), and irradiated by the 808 nm laser at a power density of 1 W cm^{-2} for 0, 30, 60, 120 and 180 s. The fluorescence spectra of SOSG were acquired with excitation at 488 nm. The fluorescence intensities of SOSG at 528 nm were quantified, and plotted to the irradiation time to evaluate the $^1O_2$ generation efficiency.

Cytotoxicity assay (MTT). The HepG2 cells were seeded in 96-well plates (5000 cells/ well) and cultured at 37 ºC for 24 h. **F1**$^{2+}$-ANP-Gal (0, 2, 4, 6, 10, 13, 19, 25, 45 and 63 μg mL^{-1}

based on the mass concentration of MEH-PPV) in DMEM medium were then added. The cells were incubated for another 24 h. 3-(4,5-Dimethylthiazol-2-yl)-2,5-diphenyltetrazolium bromide (MTT) in PBS buffer (50 μL, 1 mg mL^{-1}) was added into each well. After incubated at 37 °C for 4 h. and the solution in each well was then discarded carefully. Subsequently, DMSO (100 μL) was added into each well to dissolve the resulting purple formazan crystals. The absorbance (OD) at 490 nm in each well was acquired on a microplate reader (Tcan). Each experiment was repeated for three times.

Colocalization Analysis. To examine the intracellular location of **F1**$^{2+}$-ANP-Gal, HepG2 cells were incubated with **F1**$^{2+}$-ANP-Gal (2.2/28/58 μg mL^{-1} NIR775/MEH-PPV/F1^{2+}(BF_4^-)$_2$) for 3 h, and then costained with 2.0 μM Hoechst 33342, 200 nM Mitro-tracker (MitroDeepRed) and 1.0 μM LysoTracker®GreenDND-26 for another 20 min. The cells were washed three times with cold PBS (1×, pH 7.4). The fluorescence images were acquired using the IX73 optical microscope (Olympus, Japan) equipped with TRITC, DAPI, Cy5.5, and FITC filters.

Investigation of Blood Circulation. **F1**$^{2+}$-ANP-Gal (211/100/8 μg **F1**$^{2+}$(BF_4^-)$_2$/MEH-PPV/NIR775) in 200 μL saline was i.v. injected into heathy male mice (6-8 weeks old). After injection at 0.25, 1, 2, 4, 6, 8, 12, 18, 24, 36 and 48 h, around 1.0 mL blood from every three mice at each time point were collected by cardiac puncture in heparinized capillary tubes. The blood samples were then four-fold diluted with PBS buffer (1×, pH 7.4), and incubated with NaHS (1 mM, 6 h) to completely turn on the NIR fluorescence of NIR775 within **F1**$^{2+}$-ANP-Gal. The blood from healthy mice without injection of **F1**$^{2+}$-ANP-Gal was collected as background. With a standard curve of the fluorescence of NIR775 of H_2S-activatable **F1**$^{2+}$-ANP-Gal in the four-fold diluted blank blood versus NIR775 concentration, the concentrations of **F1**$^{2+}$-ANP-Gal remained in the blood in each time point were obtained by measuring the NIR775 fluorescence by a Spark™ 10_M Multimode Microplate Reader with $\lambda_{ex}/\lambda_{em}$ = 740/790 nm.

Imaging of Main Organs Ex vivo. Control nude mice or mice with orthotropic HepG2/Luc tumors were i.v. injected with **F1**$^{2+}$-ANP-Gal (211/100/8 μg **F1**$^{2+}$(BF_4^-)$_2$/MEH-PPV/NIR775, in 200 μL saline). Mice were sacrificed at 12 h. Main organs, including tumor, liver, kidneys,

intestines, heart, brain, lung, stomach and spleen were resected. The fluorescence and afterglow images of these organs were acquired with the IVIS Lumina XR III imaging system. The fluorescence images were acquired with a 740 nm excitation filter and 790 nm emission filter. For afterglow images, the organs were irradiated by the 808 nm laser (1 W cm^{-2}) for 1 min. After cessation of laser, the afterglow images were immediately acquired for 60 s with an open filter. Each experiment was conducted in three mice.

Fluorescence Imaging of Tissue Slices. For fluorescence imaging of tumor tissue slices, HepG2 tumor-bearing mice were sacrificed and the tumor were resected at 12 h post i.v. injection of **F1**$^{2+}$-ANP-Gal (211/100/8 μg **F1**$^{2+}$$(BF_4^-)_2$/MEH-PPV/NIR775, in 200 μL saline); For fluorescence imaging of HCC tissues, HCC specimens were infiltrated with **F1**$^{2+}$-ANP-Gal (58/28/2.2 μg mL^{-1} **F1**$^{2+}$$(BF_4^-)_2$/MEH-PPV/NIR775) in PBS buffer at 37 °C for 3 h. The isolated tumors and HCC tissues were then cut using a vibrating-blade microtome to obtain 10 μm-thickness slices. After staining with DAPI, the images were acquired with the IX73 fluorescent inverted microscope equipped with DAPI and TRITC filters.

Supplementary Notes

Supplementary Note 1. The energy transfer efficiency (ET) from donor to acceptor could be estimated using the following equation:

$$\mathrm{ET} = 1 - \frac{\int_x^y F_{\mathrm{ET}}(\lambda)d\lambda}{\int_x^y F_D(\lambda)d\lambda} \qquad (1)$$

where the $\int_x^y F_{\mathrm{ET}}(\lambda)d\lambda$ is the integral area of the donor's emission spectra in the presence of an acceptor in proximity, and the $\int_x^y F_D(\lambda)d\lambda$ is the integral area of the donor's emission spectra in the absence of the acceptor.

Based on the emission spectra in **Supplementary Figure 7a**, the integral area of the MEH-PPV's emission between 500 and 700 nm in **F1**$^{2+}$-ANP and H_2S-activated **F1**$^{2+}$-ANP was found to be 87259 and 3018826, respectively. Therefore, the ET efficiency between MEH-PPV and **F1**$^{2+}$ within **F1**$^{2+}$-ANP could be estimated to be ~97.1%.

Similarly, the integral area of NIR775's emission between 740 and 810 nm in **F1**$^{2+}$-ANP and H_2S-activated **F1**$^{2+}$-ANP was found to be 1988 and 2685884, respectively. The ET efficiency between NIR775 and **F1**$^{2+}$ within **F1**$^{2+}$-ANP was calculated to be 99.9 %.

As shown in **Supplementary Figure 7b**, the integral area of the MEH-PPV's emission between 500 and 700 nm in the H_2S-activated **F1**$^{2+}$-ANP (2.2 μg mL^{-1} NIR775) and H_2S-activated nanoparticles containing **F1**$^{2+}$$(BF_4^-)_2$/MEH-PPV (58/28 μg mL^{-1}) but without NIR775 (0 μg mL^{-1} NIR775) was found to be 3162337 and 6679437, respectively. The ET efficiency between MEH-PPV and NIR775 within the H_2S-activated **F1**$^{2+}$-ANP was calculated to be ~52.6 %, which was close to the value of 53.8 % calculated from the fluorescence lifetime method (see **Supplementary Figure 12.**).

Supplementary Note 2. The energy transfer efficiency (ET) from MEH-PPV to NIR775 could be estimated using the following equation:

$$\mathrm{ET} = 1 - \frac{\tau}{\tau_0} \qquad (2)$$

where τ_0 is the fluorescence lifetime of MEH-PPV in the H_2S-activated nanoparticles containing **F1**$^{2+}$$(BF_4^-)_2$/MEH-PPV (58/28 μg mL^{-1}) but without NIR775 (0 μg mL^{-1} NIR775), and τ is the fluorescence lifetime of MEH-PPV in the H_2S-activated **F1**$^{2+}$-ANP.

As shown in **Supplementary Figure 12c**, the τ_0 and τ was measured to be ~106 ps and ~49 ps, respectively. Accordingly, the ET from MEH-PPV to NIR775 could be calculated to be 53.8 %, which was close to the value of 52.6% calculated from the fluorescence intensity method (see **Supplementary Figure 7**).
