# Supplementary material for: H2S-activatable near-infrared afterglow luminescent probes for sensitive molecular imaging in vivo
Source: Nat Commun. 2020 Jan 23;11:446. doi: 10.1038/s41467-020-14307-y (PMC6978336; doi:10.1038/s41467-020-14307-y)
Supplement: Supplementary file 2 — Description of Additional Supplementary Files [file 41467_2020_14307_MOESM2_ESM.pdf]

Description of Additional Supplementary Files

File name: Supplementary Dataset 1

Description: Summary of reported optical imaging probes for H_2S detection *in vivo*.
