# Supplementary material for: H2S-activatable near-infrared afterglow luminescent probes for sensitive molecular imaging in vivo
Source: Nat Commun. 2020 Jan 23;11:446. doi: 10.1038/s41467-020-14307-y (PMC6978336; doi:10.1038/s41467-020-14307-y)
Supplement: Supplementary file 3 — Supplementary Data 1 [file 41467_2020_14307_MOESM3_ESM.pdf]

Supplementary Dataset 1. Summary of reported optical imaging probes for H_2S detection in vivo.

**Probe** [a]	**Emission [nm]**	**Turn-on ratio (fold)**	**LOD** [b]	**Reaction Kinetics** [c]	**Detection of H_2S in mice** [d]			**Refs.**
					Model	Administration method	Endogenous	
NIR-II@Si	900	N.D. [f]	37 nM	5 min (10 equiv. of H_2S in PBS)	Mouse tumor	Intratumoral injection (i.t.)	Yes	1
NIR-H_2S	830	58	270 nM	60 min (100 equiv. H_2S in PBS)	Mouse tumor	i.t.	Yes	2
BSOHS@Si300	717/534 [e]	15	53 nM	3 min (10 equiv. H_2S in PBS)	Mouse tumor	i.t.	Yes	3
Probe **1**	796	87	39.6 nM	25 min (60 equiv. H_2S in PBS containing 10% DMSO); $k_2 = 14.9\ M^{-1}\ s^{-1}$	Mouse tumor	i.t.	Yes	4
NIR-Az	720	200	0.26 µM	30 min (20 equiv. H_2S in PBS);	Mouse intraperitoneal cavity	Intraperitoneal injection (i.p.)	No	5
NIR-HS	723	50	38 nM	30 min (10 equiv. H_2S in PBS containing 5% CH_3CN)	Mouse intraperitoneal cavity	i.p.	No	6
Probe **1**	670	65	3.05 µM	10 min (50 equiv. H_2S in PBS containing 50% DMSO)	Mouse back	Skin-popping injection (s.p.)	No	7
Azidoluciferin	Bioluminescence	8	0.1 µM	60 min (10 equiv. H_2S in PBS)	Mouse tumor	Intravenous injection (i.v.)	Yes	8
Probe **1**	Bioluminescence	8	N.D. [f]	20 min (250 equiv. H_2S in Tris-HCl buffer)	Mouse body	i.p.	No	9
NanoPT	950	N.D. [f]	106 nM	20 min (5 equiv. H_2S in PBS)	Mouse tumor	i.t.	Yes	10
Probe **1**	542	N.D. [f]	1 µM	15 min (7 equiv. H_2S in PBS containing 1% acetone)	Mouse intraperitoneal cavity	N.D. [f]	Yes	11
Mito-HS	540	21	N.D. [f]	60 min (25 equiv. H_2S in PBS with 1% DMSO)	Mouse tumor	s.p.	Yes	12
Ru-MDB	612	86	45 nM	50 min (10 equiv. H_2S in PBS)	Mouse legs	s.p.	Yes	13
**1**-H_2S	635	7	47 µM	15 min (50 equiv. H_2S in PBS with 20% DMSO)	Mouse intraperitoneal cavity	i.p.	No	14

CHC1-UCNPs	541	16.2	0.13 μM	120 s (0.05 mg/mL probe with 60 μM H_2S in HEPES buffer)	Resected mouse livers	i.v.	Yes	15
CHS-3	Chemiluminescence	12	N.D. [f]	10 min (5 equiv. H_2S in PBS containing 20% Emerald II Enhancer)	Mouse intraperitoneal cavity	i.p.	No	16
Probe **1**	500	N.D. [f]	0.52 μM	3 min (150 equiv. H_2S in PBS containing 50% DMSO)	Mouse intraperitoneal cavity	i.p.	No	17
TMSDNPOB	592	30	1.27 μM	20 min (10 equiv. H_2S in HEPES buffer containing 50% DMSO)	Mice liver slices	N.D. [f]	Yes	18
Cy-PBA	725	N.D. [f]	21 nM	2 min (2 equiv. H_2S in PBS buffer containing 50% DMSO)	intraperitoneal cavity	i.p.	No	19
**1**$^{2+}$-SNP830-FA	830	15	0.70 μM	10 min (6.25 equiv. H_2S in PBS buffer); k_2 = ~91.6 M^{-1} s^{-1}	Mouse liver<br>Mouse tumor	i.v.	Yes	20
**F1**$^{2+}$-ANP	Afterglow	122	0.1 μM	1 min (3.5 equiv. H_2S in PBS buffer); k_2 = 1563 ± 141 M^{-1} s^{-1}	Mouse tumor	i.v.	Yes	This work

[a] The name of each probe is the one shown in each ref. [b] LOD: limitation of detection. [c] Second-order reaction rate. If there is no value reported, we just list the time to achieve the maximum activation. We also list the buffer for the reaction. [d] The probe was used to detect H_2S in mice. [e] Ratiometric fluorescent probe. [f] N.D.: Not Determined.

Supplementary References

1. Xu, G. et al. Imaging of colorectal cancers using activatable nanoprobes with second near-infrared window emission. *Angew. Chem. Int. Ed*. **57**, 3626-3630 (2018).
2. Xiong, J., Xia, L., Huang, Q., Huang, J., Gu, Y. & Wang, P. Cyanine-based NIR fluorescent probe for monitoring H_2S and imaging in living cells and in vivo. *Talanta* **184**, 109-114 (2018).
3. Wang, F. et al. Realizing highly chemoselective detection of H_2S in vitro and in vivo with fluorescent probes inside core-shell silica nanoparticles. *Biomaterials* **159**, 82-90 (2018).
4. Zhang, K. et al. A new H_2S-specific near-infrared fluorescence-enhanced probe that can visualize the H_2S level in colorectal cancer cells in mice. Chem. Sci. **8**, 2776-2781 (2017).
5. Park, C. S. et al. A near-infrared "turn-on" fluorescent probe with a self-immolative linker for the in vivo quantitative detection and imaging of hydrogen sulfide. *Biosens. Bioelectron.* **89**, 919-926 (2017).

6. Zhang, L. et al. A highly selective and sensitive near-infrared fluorescent probe for imaging of hydrogen sulphide in living cells and mice. *Sci. Rep*. **6**, 18868 (2016).
7. Sun, W. et al. A two-photon fluorescent probe with near-infrared emission for hydrogen sulfide imaging in biosystems. *Chem. Commun*. **49**, 3890-3892 (2013).
8. Tian, X., Li, Z., Lau, C. & Lu, J. Visualization of in vivo hydrogen sulfide production by a bioluminescence probe in cancer cells and nude mice. *Anal. Chem.* **87**, 11325-11331 (2015).
9. Ke, B. et al. Bioluminescence probe for detecting hydrogen sulfide in vivo. *Anal. Chem.* **88**, 592-595 (2016).
10. Shi, B. et al. Hydrogen sulfide-activatable second near-infrared fluorescent nanoassemblies for targeted photothermal cancer therapy. *Nano. Lett.* **18**, 6411-6416 (2018).
11. Jin, X. et al. Novel fluorescein-based fluorescent probe for detecting H_2S and its real applications in blood plasma and biological imaging. *Anal. Chem.* **88**, 11253-11260 (2016).
12. Wu, Z., Liang, D. & Tang, X. Visualizing hydrogen sulfide in mitochondria and lysosome of living cells and in tumors of living mice with positively charged fluorescent chemosensors. *Anal. Chem.* **88**, 9213-9218 (2016).
13. Du, Z. et al. Quantitative monitoring and visualization of hydrogen sulfide in vivo using a luminescent probe based on a ruthenium(II) complex. *Angew. Chem., Int. Ed.* **57**, 3999-4004 (2018).
14. Liu, K., Liu, C., Shang, H., Ren, M. & Lin, W. A novel red light emissive two-photon fluorescent probe for hydrogen sulfide (H_2S) in nucleolus region and its application for H_2S detection in zebrafish and live mice. *Sens. Actuator B-Chem.* **256**, 342–350 (2018).
15. Zhou, Y. et al. Inorganic-organic hybrid nanoprobe for NIR-excited imaging of hydrogen sulfide in cell Cultures and inflammation in a mouse model. *Small* **10**, 4874-4885 (2014).
16. Cao, J. et al. Chemiluminescent probes for imaging H_2S in living animals. *Chem. Sci.* **6**, 1979-1985 (2015).
17. Xie, X., Yin, C., Yue, Y., Chao, J. & Huo, F. Fluorescent probe detect distinguishly sulfite/hydrogen sulfide and thiol via two emission channels in vivo. *Sens. Actuator B-Chem.* **277**, 647–653 (2018).
18. Ji, Y., Xia, L. J., Chen, L., Guo, X. F., Wang, H. & Zhang, H. J. A novel bodipy-based fluorescent probe for selective detection of hydrogen sulfide in living cells and tissues. *Talanta* **181**, 104-111 (2018).
19. Zhang, X. et al. A highly sensitive near-infrared fluorescent probe for the detection of hydrogen sulfide and its application in living cells and mice. *New J. Chem.* **42**, 19795-19800 (2018).

20. Wu, L. et al. Engineering of Electrochromic Materials as Activatable Probes for Molecular Imaging and Photodynamic Therapy. *J. Am. Chem. Soc.* **140**, 16340-16352 (2018).
