# Supplementary material for: H2S-activatable near-infrared afterglow luminescent probes for sensitive molecular imaging in vivo
Source: Nat Commun. 2020 Jan 23;11:446. doi: 10.1038/s41467-020-14307-y (PMC6978336; doi:10.1038/s41467-020-14307-y)
Supplement: Supplementary file 4 — Reporting Summary [file 41467_2020_14307_MOESM4_ESM.pdf]

nature research

Corresponding author(s): Deju Ye

Last updated by author(s): Dec 11, 2019

Reporting Summary

Nature Research wishes to improve the reproducibility of the work that we publish. This form provides structure for consistency and transparency in reporting. For further information on Nature Research policies, see Authors & Referees and the Editorial Policy Checklist .

Statistics

For all statistical analyses, confirm that the following items are present in the figure legend, table legend, main text, or Methods section.

n/a	Confirmed	
☐	☒	The exact sample size (*n*) for each experimental group/condition, given as a discrete number and unit of measurement
☐	☒	A statement on whether measurements were taken from distinct samples or whether the same sample was measured repeatedly
☐	☒	The statistical test(s) used AND whether they are one- or two-sided<br>*Only common tests should be described solely by name; describe more complex techniques in the Methods section.*
☐	☒	A description of all covariates tested
☐	☒	A description of any assumptions or corrections, such as tests of normality and adjustment for multiple comparisons
☐	☒	A full description of the statistical parameters including central tendency (e.g. means) or other basic estimates (e.g. regression coefficient) AND variation (e.g. standard deviation) or associated estimates of uncertainty (e.g. confidence intervals)
☒	☐	For null hypothesis testing, the test statistic (e.g. *F*, *t*, *r*) with confidence intervals, effect sizes, degrees of freedom and *P* value noted<br>*Give P values as exact values whenever suitable.*
☒	☐	For Bayesian analysis, information on the choice of priors and Markov chain Monte Carlo settings
☒	☐	For hierarchical and complex designs, identification of the appropriate level for tests and full reporting of outcomes
☐	☒	Estimates of effect sizes (e.g. Cohen's *d*, Pearson's *r*), indicating how they were calculated

Our web collection on statistics for biologists contains articles on many of the points above.

Software and code

Policy information about availability of computer code

Data collection	No software was used.
Data analysis	All software used for data analysis are fully described in the materials and methods of the manuscript. Fluorescence and afterglow images were analyzed using the Living Image Software (4.5.2, PerkinElmer, MA, U.S.A). Image-Pro Plus was used to analyse the western plot data. Statistical calculations were performed using GraphPad Prism 6 (GraphPad Software Inc., CA, USA).

For manuscripts utilizing custom algorithms or software that are central to the research but not yet described in published literature, software must be made available to editors/reviewers. We strongly encourage code deposition in a community repository (e.g. GitHub). See the Nature Research guidelines for submitting code & software for further information.

Data

Policy information about availability of data

All manuscripts must include a data availability statement. This statement should provide the following information, where applicable:

- Accession codes, unique identifiers, or web links for publicly available datasets
- A list of figures that have associated raw data
- A description of any restrictions on data availability

The authors declare that all related to this study are available in the article/and or its supplementary information files.

Field-specific reporting

Please select the one below that is the best fit for your research. If you are not sure, read the appropriate sections before making your selection.

☒

Life sciences study design

All studies must disclose on these points even when the disclosure is negative.

Sample size	No sample size calculations were performed. The sample size (n) of each experiment is provided in the corresponding figure captions in the paper and supplementary information. Sample sizes were chosen to support meaningful conclusions.
Data exclusions	No data was excluded from this study.
Replication	Experiments were repeated at least three independent experiments with similar results. All experiments were reproduced to reliably support conclusions stated in the manuscript.
Randomization	The mice were randomly selected and then divided into experimental groups for further treatment.
Blinding	Investigators were not blinded to group allocation during data collection and analysis, due to limited personnel.

Reporting for specific materials, systems and methods

We require information from authors about some types of materials, experimental systems and methods used in many studies. Here, indicate whether each material, system or method listed is relevant to your study. If you are not sure if a list item applies to your research, read the appropriate section before selecting a response.

Materials & experimental systems

n/a	Involved in the study	
☐	☒	Antibodies
☐	☒	Eukaryotic cell lines
☒	☐	Palaeontology
☐	☒	Animals and other organisms
☐	☒	Human research participants
☒	☐	Clinical data

Methods

n/a	Involved in the study	
☒	☐	ChIP-seq
☒	☐	Flow cytometry
☒	☐	MRI-based neuroimaging

Antibodies

Antibodies used	Rabbit Anti-CBS antibody (Catalog # ab135626) and Rabbit Anti-Cystathionase/CTH antibody (Catalog # ab151769) were purchased from Abcam (Cambridge,UK).
Validation	Validation details of the primary antibodies are available on the manufacturers' websites:<br>https://www.abcam.cn/cbs-antibody-ab135626.html#description_images_1<br>https://www.abcam.cn/cystathionasecth-antibody-ab151769.html

Eukaryotic cell lines

Policy information about cell lines

Cell line source(s)	Human hepatic cancer HepG2 cells and Human lung cancer A549 cancer cells were purchased from from Stem Cell Bank, Chinese Academy of Sciences (Shanghai, China).
Authentication	All cell lines were authenticated by the supplier using Short Tandem Repeat test.
Mycoplasma contamination	All cell lines tested were negative for mycoplasma contamination.
Commonly misidentified lines (See ICLAC register)	No commonly misidentified cell lines were used.

Animals and other organisms

Policy information about studies involving animals; ARRIVE guidelines recommended for reporting animal research

Laboratory animals	Animal experiments were carried out under the guidelines of the Institutional Animal Care and Use Committee (IACUC) of Nanjing University. BALB/c mice (~5-6 weeks old) were purchased from the Model Animal Research Center (MARC) of Nanjing University in China and used for all the in vivo studies.
Wild animals	The study did not involve wild animals.

Field-collected samples	Irrelevant to experiments.
Ethics oversight	The Institutional Animal Care and Use Committee (IACUC) of Nanjing University approved and provided guidance on the study protocol.

Note that full information on the approval of the study protocol must also be provided in the manuscript.

Human research participants

Policy information about studies involving human research participants

Population characteristics	Patients with human hepatocellular carcinoma (HCC):<br>Age: 71, 56, 52, 58, 51, 58, 53, 56, 70, 67; Gender: male, male, male, male, male, male, male, male, male, female;<br>Patients with colorectal cancer (CRC):<br>Age: 54, 50, 77, 69, 72, 77, 64, 64, 69, 57; Gender: female, female, male, male, male, male, male, female, male, female;<br>Healthy participants:<br>Age: 67, 51, 66, 53, 76, 44, 50, 56, 54, 69; Gender: male, male, male, male, male, male, female, male, female, male;<br>Patients with HCC for acquiring specimens:<br>Age: 58, 58, 56, 67; Gender: male, male, male, female;
Recruitment	Adult healthy and patients donors were recruited by Affiliated Drum Tower Hospital of Nanjing University.<br>Patients volunteers inclusion criteria:<br>1. Aged between 18 and 80.<br>2. Tissue biopsy is diagnosed as a patient with liver cancer or colon cancer.<br>3. The patient has not received any form of treatment prior to blood sample collection.<br>4. The patient is scheduled to undergo surgery in Affiliated Drum Tower Hospital of Nanjing University.<br>Healthy participants inclusion criteria:<br>1. Aged between 18 and 80.<br>2. No history of liver, intestinal or metabolic diseases.<br>3. No drinking history.<br>4. No drug history during the evaluation period.<br>5. Normal liver biochemical examination and normal colonoscopy.<br>Patients or healthy participants that did not meet the above requirements would be excluded.
Ethics oversight	The study was approved by institutional review board (IRB) of Affiliated Drum Tower Hospital of Nanjing University, and all subjects provided written informed consent under institutional review board prior to sample collection.

Note that full information on the approval of the study protocol must also be provided in the manuscript.
